# The impacts of donor transitions on health systems in middle-income countries: a scoping review

**DOI:** 10.1093/heapol/czac063

**Published:** 2022-07-29

**Authors:** Hanna E Huffstetler, Shashika Bandara, Ipchita Bharali, Kaci Kennedy Mcdade, Wenhui Mao, Felicia Guo, Jiaqi Zhang, Judy Riviere, Liza Becker, Mina Mohamadi, Rebecca L Rice, Zoe King, Zoha Waqar Farooqi, Xinqi Zhang, Gavin Yamey, Osondu Ogbuoji

**Affiliations:** Gillings School of Global Public Health, University of North Carolina at Chapel Hill, 135 Dauer Drive, Chapel Hill, NC 27599, USA; Center for Policy Impact in Global Health, Duke Global Health Institute, Duke University, 310 Trent Drive, Durham, NC 27710, USA; Center for Policy Impact in Global Health, Duke Global Health Institute, Duke University, 310 Trent Drive, Durham, NC 27710, USA; Faculty of Medicine and Health Sciences, McGill University, 3605 Rue de la Montagne, Montreal, QC H3G 2M1, Canada; Center for Policy Impact in Global Health, Duke Global Health Institute, Duke University, 310 Trent Drive, Durham, NC 27710, USA; Center for Policy Impact in Global Health, Duke Global Health Institute, Duke University, 310 Trent Drive, Durham, NC 27710, USA; Center for Policy Impact in Global Health, Duke Global Health Institute, Duke University, 310 Trent Drive, Durham, NC 27710, USA; Center for Policy Impact in Global Health, Duke Global Health Institute, Duke University, 310 Trent Drive, Durham, NC 27710, USA; Center for Policy Impact in Global Health, Duke Global Health Institute, Duke University, 310 Trent Drive, Durham, NC 27710, USA; Center for Policy Impact in Global Health, Duke Global Health Institute, Duke University, 310 Trent Drive, Durham, NC 27710, USA; Center for Policy Impact in Global Health, Duke Global Health Institute, Duke University, 310 Trent Drive, Durham, NC 27710, USA; Department of Industrial and Systems Engineering, North Carolina State University, 915 Partners Way, Raleigh, NC 27606, USA; Gillings School of Global Public Health, University of North Carolina at Chapel Hill, 135 Dauer Drive, Chapel Hill, NC 27599, USA; Center for Policy Impact in Global Health, Duke Global Health Institute, Duke University, 310 Trent Drive, Durham, NC 27710, USA; Center for Policy Impact in Global Health, Duke Global Health Institute, Duke University, 310 Trent Drive, Durham, NC 27710, USA; Center for Policy Impact in Global Health, Duke Global Health Institute, Duke University, 310 Trent Drive, Durham, NC 27710, USA; Center for Policy Impact in Global Health, Duke Global Health Institute, Duke University, 310 Trent Drive, Durham, NC 27710, USA; Center for Policy Impact in Global Health, Duke Global Health Institute, Duke University, 310 Trent Drive, Durham, NC 27710, USA

**Keywords:** Donor transitions, middle-income countries, health systems

## Abstract

As countries graduate from low-income to middle-income status, many face losses in development assistance for health and must ‘transition’ to greater domestic funding of their health response. If improperly managed, donor transitions in middle-income countries (MICs) could present significant challenges to global health progress. No prior knowledge synthesis has comprehensively surveyed how donor transitions can affect health systems in MICs. We conducted a scoping review using a structured search strategy across five academic databases and 37 global health donor and think tank websites for literature published between January 1990 and October 2018. We used the World Health Organization health system ‘building blocks’ framework to thematically synthesize and structure the analysis. Following independent screening, 89 publications out of 11 236 were included for data extraction and synthesis. Most of this evidence examines transitions related to human immunodeficiency virus/Acquired Immune Deficiency Syndrome (AIDS; *n* = 45, 50%) and immunization programmes (*n* = 14, 16%), with a focus on donors such as the Global Fund to Fight AIDS, Tuberculosis and Malaria (*n* = 26, 29%) and Gavi, the Vaccine Alliance (*n* = 15, 17%). Donor transitions are influenced by the actions of both donors and country governments, with impacts on every component of the health system. Successful transition experiences show that leadership, planning, and pre-transition investments in a country’s financial, technical, and logistical capacity are vital to ensuring smooth transition. In the absence of such measures, shortages in financial resources, medical product and supply stock-outs, service disruptions, and shortages in human resources were common, with resulting implications not only for programme continuation, but also for population health. Donor transitions can affect different components of the health system in varying and interconnected ways. More rigorous evaluation of how donor transitions can affect health systems in MICs will create an improved understanding of the risks and opportunities posed by donor exits.

**Key messages**

- Donor transitions are influenced by the actions of both donors and country governments, with impacts on every component of the health system.
- Successful transition experiences show that leadership, planning, and pre-transition investments in a country’s financial, technical, and logistical capacity are vital to ensuring smooth transition.
- Poor transitions can result in shortages in financial resources, medical product and supply stock-outs, service disruptions, and shortages in human resources, with resulting implications not only for programme continuation, but also for population health.

## Introduction

Recent decades have witnessed tremendous progress in global health—from the dramatic scale-up of the global human immunodeficiency virus (HIV) response to impressive declines in the child mortality rate. Contributing to these gains has been the significant overall growth in development assistance for health (DAH) over the past 30 years (Bendavid *et al.*, 2017; Watkins *et al.*, 2018). Evidence suggests that DAH is associated with positive health outcomes, including longer life expectancy and reduced under-5 mortality (Bendavid and Bhattacharya, 2014). However, there is an active shift in the health financing landscape where donors are increasingly re-evaluating the provision of DAH to countries viewed as capable of self-financing. This has contributed to DAH making up an increasingly smaller share of total health financing in both low-income countries and middle-income countries (MICs; Schäferhoff *et al.*, 2019). Notably, annual increases in DAH have generally slowed over the past decade, with contributions plateauing at an annualized growth rate of 1.2% since 2012 (Global Burden of Disease Health Financing Collaborator Network, 2021). While the emergence and spread of SARS-CoV-2 (COVID-19) in early 2020 prompted significant contributions to the DAH pool globally, the long-term impact of the pandemic on DAH remains uncertain (Ogbuoji *et al.*, 2021).

Transitions away from DAH are inflection points when specific donors begin to withdraw concessional technical and financial assistance for health programming from a recipient country, and that country begins to draw from other sources, such as public spending or commercial loans, to replace the loss of DAH. The nature of a given transition can vary, depending on the capacity of the recipient country and the donor-specific processes used to facilitate the withdrawal of assistance (e.g. co-financing). There are no standard transition criteria, and the policies and thresholds for triggering transition vary widely according to the donor (McDade *et al.*, 2020). To determine a country’s ability to self-finance, some donors examine health data alongside economic indicators. For example, the Global Fund to Fight Acquired Immune Deficiency Syndrome (AIDS), Tuberculosis and Malaria (Global Fund) uses a country’s gross national income per capita alongside indicators of disease burden to determine eligibility for aid (Schaferhoff *et al.*, 2019). Other donors, like the USA’ President’s Emergency Plan for AIDS Relief (PEPFAR), lack a clear transition policy and instead focus more on building country capacity for self-financing (McDade *et al.*, 2020).

Despite perceived country readiness, evidence suggests that poorly planned transitions away from DAH could present significant challenges to sustaining progress in global health, since transitioning countries may struggle to maintain necessary investments in domestic health programming (Resch and Hecht, 2018). Three-quarters of the world’s population and 62% of the world’s poor now live in MICs that are on the edge of many DAH eligibility thresholds (World Bank, 2022). If donor exits within these countries are not well managed, poor and marginalized populations in MICs may bear the brunt of downstream disruptions to health programming, including programmes for immunization, family planning, and HIV/AIDS (Sumner, 2011). Such transition-related disruptions can have negative implications for population health, threatening to curb progress previously achieved through DAH-supported programming (Flanagan *et al.*, 2018). One analysis found that while over a dozen MICs will graduate from multilateral development assistance in the coming years, some of these countries are highly vulnerable to health and economic ‘shocks’ when they transition due to large pockets of poverty, high child and maternal mortality, and limited domestic resource mobilization capacity (Yamey *et al.*, 2018). Economic growth is not synonymous with the capacity of a country to manage transitions, and a premature transition from DAH or a transition without sufficient support from the exiting donor leading up to or following a transition could prove counterproductive. For example, of the 37 countries that have graduated from International Development Association (IDA) since 1960, twelve have needed to re-enter IDA support following adverse developments occurring after graduation (IDA, 2022).

The management of donor transitions is complex, and a wide range of factors—from political will to human resource capacity—could impede successful transition processes (Gotsadze *et al.*, 2019). These vulnerabilities, combined with the risks presented by poorly managed transitions, warrant a closer look at what we know about the impacts of donor transitions on health systems in MICs. A growing body of scholarship seeks to understand and analyse the experience of country transitions away from DAH. This body of scholarship is vast in scope, covering dozens of health areas, donors, geographies and points in the transition process. To the best of our knowledge, there has been no previously published synthesis that characterizes the nature and scope of evidence on how transitions can influence health systems in MICs or the downstream effects on health outcomes. We conducted a scoping review to characterize the literature on the impacts of donor transitions on the health system and population health outcomes in MICs that are currently transitioning or have previously transitioned from DAH.

## Methods

Sharing many of the same processes as systematic reviews, scoping reviews use rigorous and transparent methods to comprehensively identify and analyse relevant literature addressing a specific research question (Pham *et al.*, 2014). Given that transition is a relatively recent phenomenon and that scholarship in this area is mostly in the grey literature where methods are not consistently reported, it is difficult to assess the quality of studies or risk of bias. Therefore, a scoping review allowed us to synthesize the available evidence without the requirement to assess quality as would have been the case with a systematic review. We used the step-by-step approach for performing scoping reviews outlined by Levac, Colquhoun, and O’Brien, which builds upon the methodological framework developed by Arksey and O’Malley (Arksey, 2003; Levac *et al.*, 2010). We specifically followed these five stages: (1) identifying a research question, (2) searching for studies, (3) selecting the studies, (4) charting the data and (5) collating, summarizing and reporting the results.

### Identifying the research question

The process of defining our aims for this review was iterative, a key characteristic of scoping reviews (Grant and Booth, 2009). Our original research question sought to characterize the nature of available research and evidence on donor transitions in the health sector. This initial research question was progressively refined through team discussion following an initial review of available evidence. Through discussion, we narrowed the focus of our research to specifically examine evidence of the impacts of donor transitions on the health system and population health outcomes in MICs that are currently transitioning or have previously transitioned from DAH. In this review, donor transitions—also referred to as donor exits, phase-out, or graduation—are defined as processes that involve decreases in the volume of donor-provided resources or support, such as financial backing or technical expertise.

### Conceptual framework

As our review aimed to assess the impacts of donor transitions on health systems, we used the World Health Organization (WHO) health systems framework to structure our methodology, including the data extraction, analysis and organization of results. The WHO health systems framework conceptualizes the health system as being made up of six ‘building blocks’. These blocks are (1) leadership and governance, (2) health financing, (3) health workforce, (4) information and research, (5) medical products and technology and (6) service delivery (WHO, 2007). In addition to these building blocks, we also examined the impacts of donor transitions on health outcomes, which are referenced as a goal in the WHO health systems framework (WHO, 2007). In the context of this review, influences on population health are defined as any factor contributing to a noted change in or maintenance of a given health outcome within a defined population, specifically resulting from consequences incurred during and/or following a donor transition. Since the population health impact differs by the health or disease focus of the programme, we limited our analysis of population health impacts to the specific indicators reported for each programme.

### Search strategy

Our research team developed a comprehensive search strategy with the help of experts at the libraries of the authors’ institute. Keywords were used to search five electronic databases that include literature from multiple disciplines relevant to health-related donor transitions: Scopus, Global Health, Embase, Political Science Complete and the Public Affairs Information Service (PAIS) Index. Our search strategy used a series of key terms clustered around five key areas: change, funding, support, health and impact. The team identified these key areas and sought to ensure the principal components of our research question were addressed. Across these areas, 82 different key words were chosen based on literature reviews and team input since a variety of phrases can be used to describe donor transitions. When translated into a search string, terms are connected across conceptual areas using Boolean operators, such that all terms within a given area are connected by an ‘OR’ operator, and areas are connected to one another with an ‘AND’ operator. Operationally there is no hierarchy between the conceptual areas or the search terms grouped within. Supplementary Appendix 1 outlines the search terms used, grouped by conceptual area, and also documents the search strings used in each database. All database searches were limited to English. All searches were run in October 2018 and included any results with a publication date between January 1990 and October 2018.

Our initial, informal literature search suggested that there have been few empirical studies examining the impacts of donor transitions. To complement the results from our database searches of the peer-reviewed literature, we conducted a comprehensive search of the grey literature using a relevant subset of 30 key terms to run systematic, website-specific searches across nearly 40 websites of prominent global health service providers, donors and think tanks. Supplementary Appendix 2 provides an overview of the key terms search strategy and a list of the websites searched. Like the database searches, search strings were developed by using Boolean operators to connect terms within and between conceptual areas. Multiple types of grey literature publications—including reports, policy briefs as well as presentation materials—were considered. The reference lists of all included publications were hand-checked to identify additional publications that might not have been indexed in the database and website searches (Horsley *et al.*, 2011).

### Study selection

To identify the relevant publications from the peer-reviewed and grey literature, two authors independently reviewed all titles and abstracts generated from the searches. Publications were excluded on initial screening only if the reviewer could determine from the title and abstract that the study either (1) did not meet the inclusion criteria or (2) met any of the exclusion criteria (Table 1). Given the broad scope of this review and known characteristics of the body of literature being searched, the inclusion and exclusion criteria were intentionally broad to capture as many relevant studies as possible across donors, health areas and geographies. For example, we intentionally did not implement criteria around study design and methods, as we knew we would pull from the grey literature where methods are not systematically reported. We also did not limit our search by transition criteria, given that the thresholds utilized by donors can vary widely. This allowed us to capture different types of donors, including bilateral donors [e.g. United States Agency for International Development (USAID)], multilateral donors [United Nations Population Fund (UNFPA), the World Bank and WHO], major global health initiatives (e.g. the Global Fund and Gavi), and private donors (e.g. Bill and Melinda Gates Foundation). After initial screening, the results were further refined by two authors who independently evaluated the full text for inclusion. This screening process was completed between October 2018 and November 2018. Disagreements between the reviewers were resolved by discussion with a third author. Uncertainties related to study selection were discussed among co-authors to ensure consistency in applying the inclusion and exclusion criteria.

**Table 1.** Inclusion and exclusion criteria

Inclusion criteria	Exclusion criteria
Focuses on health-related donor transitions Explicitly reports on the impacts of and responses to donor transition related to at least one of the six ‘building blocks’ in the WHO health systems framework Focuses on countries that were either transitioning at the time of the study or were in the post-transition phase during the study period	Focuses on donor transitions outside the health or health-related sectors Does not explicitly report on the impacts of and country responses to donor transition related to any of the six ‘building blocks’ in the WHO health systems framework Focuses on countries that were in the pre-transition stage during the study period Source is not written in English Op-eds, books and dissertations

### Data extraction

A standardized data extraction form was developed and refined among the research team. Team members extracted descriptive information from individual publications related to the nature and scope of the study (e.g. citation information, health and donor foci) as well as any impacts on the health system in the context of transition. Data extraction was completed between December 2018 and May 2019. Prior to starting data collection, the research team piloted the data extraction form on two publications to ensure consistency across reviewers. The research team met several times throughout the data extraction period and iteratively updated the data extraction form as needed. If publications presented experiences of both (1) countries that have not yet transitioned away from DAH and (2) countries that are actively transitioning or have previously transitioned away from DAH, only those in the latter category were considered for data extraction.

### Summarizing and reporting results

Our analytical approach was to combine basic descriptive statistics (i.e. distribution of frequencies) and qualitative content analysis. Content analysis was guided by the WHO health systems framework; the team identified, discussed and defined themes that emerged within each of the WHO health system ‘building blocks’ (WHO, 2007). The research team collaboratively synthesized the information and discussed the policy implications of the findings. The analysis and synthesis processes took place between May 2019 and December 2019.

## Results

**Figure 1.**
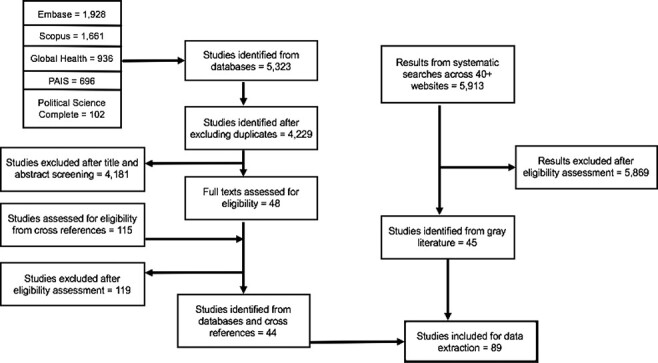
Flow diagram of publication selection

The search strategy generated a total of 11 236 publications from both academic databases and internet searches that were assessed for eligibility using the inclusion criteria. A total of 10 169 publications were excluded from the analysis: 1094 of those excluded were duplicate publications while 4181 peer-reviewed publications and 5869 publications from the grey literature were deemed ineligible based on review of their titles, abstracts and full text. Ineligible publications included those that did not discuss the health systems’ impacts of transitions away from donor development for health or those that examined countries that had not yet started to transition away from DAH. Following the exclusion process, 89 publications from both the peer-reviewed studies and grey literature were ultimately included for data extraction (Figure 1).

**Figure 2.**
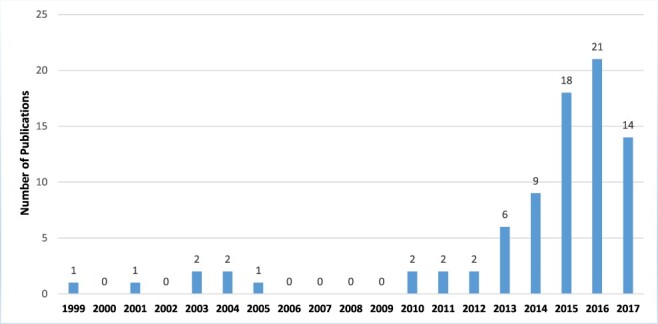
Number of publications by year^a^

^a^Five publications included in this review do not have an associated publication year

Nearly two-thirds of the included publications (*n* = 66, 74%) came from the grey literature, while the remaining third (*n* = 23, 26%) were from peer-reviewed journals. Scholarly interest in the impacts of donor transitions appears to have increased since 2013; among the publications included in this review, we found an average of one publication per year between 1999 and 2012 and an average of 13.6 per year between 2013 and 2017 (Figure 2). In terms of geographic region, nearly one-fifth of publications examined transition experiences from the WHO region of Europe and Central Asia (*n* = 16, 18%). Other major WHO regions examined included Latin America and the Caribbean (*n* = 13, 15%), Sub-Saharan Africa (*n* = 11, 12%), South Asia (*n* = 8, 9%), East Asia and the Pacific (*n* = 4, 5%) and the Middle East and North Africa (n = 2, 2%). The remaining publications (*n* = 35, 39%) looked at country experiences in more than one region. All included publications addressed transition experiences within countries that had achieved middle-income status.

More than half of the publications included in this review (*n* = 46, 52%) focused solely on transition experiences related to HIV/AIDS programmes. Others focused on transitions in donor funding of immunization (*n* = 15, 17%), family planning (*n* = 9, 10%) and nutrition (*n* = 3, 3%) programmes. The remaining publications (*n* = 16, 19%) addressed donor transitions related to other or multiple health issues, including, but not limited to, transitions related to tuberculosis (TB) and malaria programming. Among the papers included, the Global Fund (*n* = 24, 27%) was the primary donor examined, followed by Gavi (*n* = 17, 19%), the USAID (*n* = 14, 16%), PEPFAR (*n* = 10, 11%) and the Bill and Melinda Gates Foundation (BMGF) (*n* = 6, 7%). All other publications (20%) discussed findings related to transitions from other donors, including the United Nations Development Programme (UNDP) and the World Bank.

Among the six WHO health systems ‘building blocks’, most publications reported on the relationship between donor transitions and leadership and governance (*n* = 77, 87%) or health financing (*n* = 71, 80%). Impacts related to service delivery (*n* = 53, 60%) and medical products and technology (*n* = 42, 47%) were also discussed frequently among the included publications. Just over one-third of publications addressed healthcare workforce (*n* = 31, 35%) and close to one-third addressed information systems and research (*n* = 28, 31%). Impacts on health outcomes (*n* = 6, 7%) were not frequently addressed in the context of donor transition.

All descriptive statistics are summarized in Table 2. Key findings from individual publications were synthesized by each WHO building block and disease focus in Supplementary Appendix 3 (Supplementary Appendix Tables A1–A7).

**Table 2.** Publication characteristics (*N* = 89)

Characteristic	*n* (%)
Source	
Peer-reviewed sources	23 (26%)
Grey literature sources	66 (74%)
Health topic	
HIV/AIDS	46 (52%)
Immunization	15 (17%)
Family planning	9 (10%)
Nutrition	3 (3%)
Others^a^	4 (5%)
Multiple health topics	12 (14%)
Donor	
Global Fund	24 (27%)
Gavi	17 (19%)
USAID	14 (16%)
PEPFAR	10 (11%)
BMGF	6 (7%)
Other^b^	9 (10%)
Multiple donors	9 (10%)
Geographic region	
Europe and Central Asia	16 (18%)
Latin America and the Caribbean	13 (15%)
Sub-Saharan Africa	11 (12%)
South Asia	8 (9%)
East Asia and the Pacific	4 (5%)
Middle East and North Africa	2 (2%)
Multiple regions	35 (39%)
Health system component^c^	
Leadership and governance	77 (87%)
Healthcare financing	71 (80%)
Service delivery	53 (60%)
Medical products and technology	42 (47%)
Healthcare workforce	31 (35%)
Information systems and research	28 (31%)
Health outcomes	6 (7%)

a Includes tuberculosis, malaria, eye care and syringe programming.

b Includes UNDP, World Bank, Swiss Red Cross and World Food Program.

c Reflects the frequency with which publications presented data that could be thematically categorized under each health system component. Nearly all publications addressed more than one health system component.

### Leadership and governance

Leadership and governance involve the provision of oversight and guidance to the entire country’s health system—through policy, regulation, system design and accountability—with the end goal of protecting public health (WHO, 2007). Leadership from either donors or governments during or following donor exit thus has important cascading effects that can be felt throughout the health system—from the continuation of programming through sustained investment to the assurance of equity in the delivery of services (Avila *et al.*, 2012; Curatio International Foundation, 2015; Bennett *et al.*, 2015a; Eurasian Harm Reduction Network, 2016a).

Across publications included in this review, shortcomings in leadership from both the recipient country and existing donor were reported to result in delays in programme continuation, gaps in health financing and technical capacity, as well as changes or disruptions in health service delivery in the post-transition period (Alkenbrack and Shepherd, 2005; Curatio International Foundation, 2015; Eurasian Harm Reduction Network, 2015,2016b; The World Food Programme, 2003; Vogus and Graff, 2015). Notably, lack of clear planning and communication from the donor side was shown to negatively influence the sustainability of programming. This impact can be seen in the PEPFAR transition in South Africa where lack of clear planning and a short timeframe provided for the transition created resentment among country stakeholders and caused disruptions to treatment regimens (Vogus and Graff, 2015). Another instance of shortcomings in leadership followed the Global Fund’s departure from Serbia in 2014: lack of leadership and planning on the part of both the donor and country government resulted in over 50 non-governmental organizations (NGOs) losing around 90% of their funding, forcing them to close and stop providing harm reduction services to a significant proportion of key populations (Eurasian Harm Reduction Network, 2016c). In Botswana, despite financial preparation in the pre-transition period from the World Food Program, there were still unfilled gaps in technical capacity post-transition, leading to drops in programme efficiency and programme management (The World Food Programme, 2003). Further, changes in political leadership during or following transition can also affect the sustainability of health programming as political willingness to invest in and/or prioritize health programming is not static (Global Fund, 2018).

While lack of leadership can lead to poorly managed transitions, a range of governance principles were reported to facilitate the sustainability of health programming beyond the departure of a donor. These include political commitment, domestic ownership of health programming, legislative and policy support for transitioning programmes and coordination between donor, government and civil society stakeholders (Alkenbrack and Shepherd, 2005; Avila *et al.*, 2012; Sgaier *et al.*, 2013; UNAIDS, 2013; Bennett *et al.*, 2015a; UNDP, 2016). For example, the government of Swaziland used the Clinton Health Access Initiative’s ceiling price list as a benchmarking tool to support transition. The government used the tool not only to develop robust best practices for long-term procurement of essential medicines, but also to institutionalize these best practices within the Ministry of Health to sustain procurement processes beyond the transition period (UNAIDS, 2013). In India, the alignment of donor and government priorities using evidence-based planning, as well as iterative learning from initial phases of transition, helped to ensure the smooth transfer of a national HIV programme from BMGF to the Government of India (Sgaier *et al.*, 2013; Bennett *et al.*, 2015a). In another example, as the Global Fund’s support to Kazakhstan decreased, the government of Kazakhstan, in coordination with both donor and civil society stakeholders, used the state health programme to engage NGOs and provide funding for capacity building for NGOs (UNAIDS, 2013).

### Healthcare financing

Health financing has three key functions: the mobilization of financing, pooling of financing, and purchasing of services. Sufficient levels of health financing ensure that the health system has enough funding to equitably cover the individual and collective needs of the population and safeguards the accessibility of effective, high-quality health services for all (WHO, 2007). Donor transitions have a direct influence on health financing within a country and affect not only the budgets for health programming but also financial management capacity, subsequent service delivery and human resources (Crye, 2011; Kallenberg and Cornejo, 2015; Kallenberg *et al.*, 2016; Health Policy Project, 2016a; Teixeira, 2017). Reductions in donor funding were also seen to affect the funding allocated to programming by civil society organizations (CSOs), which is often aimed at serving vulnerable populations that otherwise experience difficulty accessing necessary health services (STOPAIDS, 2016; TB Europe Coalition, 2016).

Faced with the responsibility to finance health programming with domestic or other funds, countries use different methods to ensure financial sustainability of programming during and after donor transitions. Among the publications included in this review, one commonly reported mechanism is earmarking or adopting dedicated budget lines to ensure adequate funding for specific programmes and organizations following donor exits (Sgaier *et al.*, 2013; UNAIDS, 2013; Bennett *et al.*, 2015b). For example, the Albanian government ensured financial sustainability for vaccines following its transition from Gavi by assigning a separate budget line item for vaccines (Curatio International Foundation, 2015). In Macedonia, to strengthen HIV prevention efforts for key populations after the departure of the Global Fund, the Ministry of Health took steps to register NGOs to make them eligible to receive funding for HIV prevention from the government, thereby creating a social contracting mechanism between the government and NGOs that had not previously existed (Open Society Foundations, 2015). Beyond earmarking and registering organizations, governments also introduced innovative methods of mobilizing domestic resources to fill the funding gap left by donor exits. For example, Kenya proposed a levy on airline tickets and passed a bill in 2012 to allocate 1% of tax revenue to help fill the domestic financing gap for HIV/AIDS programming caused by reduction in overall donor funding (Katz *et al.*, 2014).

There was a reported positive relationship between efforts to build financial and project management capacity within governments and the effective management of healthcare financing following donor exits (UNDP, 2016). For example, in preparation for its transition, UNDP who acted as the principal recipient and managed the Global Fund programs in El Salvador, successfully built capacity within the government to manage funds from the Global Fund and implement programmes for HIV and TB after transitioning management from UNDP (UNDP, 2016). However, lack of preparatory efforts and commitment can lead to critical financing gaps, ultimately resulting in sustainability challenges (Eurasian Harm Reduction Network, 2015; Kallenberg and Cornejo, 2015; Kazadi, 2015; Burrows *et al.*, 2016; STOPAIDS, 2016; TB Europe Coalition, 2016). For example, in Romania, Serbia and Albania, combined lack of funding and political will led to the collapse of HIV prevention programmes for key populations following the departure of the Global Fund, with subsequent impacts on HIV-related health outcomes (Eurasian Harm Reduction Network, 2016a; 2016b; 2016c). Even when there is political will, governments may still struggle to close the funding gap left by donor exits due to an insufficient capacity for domestic resource mobilization (Global Fund, 2018). Such challenges are compounded by lack of capacity in financial management, which may affect a government’s ability to develop effective financial management and resource mobilization strategies following donor exits (Cernuschi *et al.*, 2018). Despite this recognition, building domestic technical capacity in this area is not necessarily prioritized within donors’ investments in supporting country transitions (Cernuschi *et al.*, 2018).

### Healthcare workforce

Behind every well-functioning health system is a strong healthcare workforce composed of health service providers, health management workers and support workers. These ‘human resources for health’ are crucial to the effective functioning of the health system (WHO, 2007). Donor transitions are reported to impact the healthcare workforce by affecting the number of staff available as well as their capacity to provide services once donor-provided technical and resources are gone. While some impacts can be positive—such as improving and maintaining technical capacity during and following the transition away from an exiting donor—all too frequently the impacts identified in this review were negative.

In some countries, technical capacity and human resources for health were built up over the period of a donor’s investment. However, during and following the transition period, domestic financial resources in these countries were not necessarily allocated to sustaining or expanding human resources for health, which led to gaps in staffing and technical capacity following donor transitions (Alkenbrack and Shepherd, 2005; Levin *et al.*, 2010; Torpey *et al.*, 2010; Cairney and Kapilashrami, 2014; Curatio International Foundation, 2014; Kavanagh, 2014; Kazadi, 2015; Republic of Guyana, 2015; Vogus and Graff, 2015; Bennett *et al.*, 2015a; 2015b; Bliss and Peck, 2016; UNAIDS, 2016; Health Policy Project, 2016b; Eurasian Harm Reduction Network, 2016d; Open Society Foundations, 2017; TB Europe Coalition, 2017). Loss in donor funding has led to health worker salary cuts (Cairney and Kapilashrami, 2014; Open Society Foundations, 2015; Bennett *et al.*, 2015a; Bliss and Peck, 2016; TB Europe Coalition, 2017), reduced education and training (Curatio International Foundation, 2014; Open Society Foundations, 2015; Bennett *et al.*, 2015b), and decreased staff motivation and incentives (Blanchet and James, 2014; amFAR, 2015; Curatio International Foundation, 2015; Kazadi, 2015; Biradavolu *et al.*, 2017; Wang *et al.*, 2017). For example, in Botswana, PEPFAR previously supported the salaries of nearly 150 planning and strategic information staff within the Ministry of Health. However, when PEPFAR decreased its funding to Botswana following a shift towards a more country-owned response, the government faced challenges in filling these positions due to turnover and significant gaps in technical planning and management capacity (Vogus and Graff, 2015).

In several countries, human resources for health were limited during and following transition due to a mismatch between the employment structures of the country government and the parallel structures developed by donor agencies to deliver healthcare services. Despite these mismatches, some country governments have adapted their post-transition plans to retain workers previously funded via donor funding. For example, as part of Brazil’s transition from the Global Fund, the government created municipal positions that were formerly funded by the donor, resulting in expanded institutional capacity for the domestic response to malaria (Lewis *et al.*, 2015). However, other countries struggled to retain the human resources that were previously funded via donor funding. For example, the Namibian government had a much more difficult time funding the health worker positions previously funded by either Global Fund or PEPFAR (Cairney and Kapilashrami, 2014). These human resource challenges do not occur in a vacuum and often have resultant impacts on health services provision. In some countries, the impact of donor exits on workforce capacity and quantity led to lower-quality services and a reduction in services provided (Curatio International Foundation, 2014; Katz *et al.*, 2014; Kavanagh, 2014; amFAR, 2015; UNAIDS, 2016; Eurasian Harm Reduction Network, 2016d).

While the examples mentioned above are largely negative, for several countries, capacity building and human resources strengthening were embedded into the graduation strategy or the transition process. For example, among USAID transitions from family planning support, graduation strategies emphasized strengthening the human and institutional capacity to implement and manage activities and included a variety of approaches to transfer knowledge and skills to the host country. These activities led to successful efforts within countries such as Nicaragua, Honduras, Indonesia and Peru to enhance NGO capacity to continue operating and to achieve financial sustainability long after USAID’s departure (Chaudry *et al.*, 2012).

### Medical products and technology

Alongside a strong healthcare workforce, strong health systems require equitable access to high-quality and cost-effective essential medicines, vaccines and technologies (WHO, 2007). Among the publications included in this study, the impacts of transition on medical products and technology largely focus on procurement and supply chain practices in the post-transition period. Stock-outs of medicines and equipment were commonly reported as a consequence of the withdrawal of donor support across different health programming areas (Curatio International Foundation, 2015; Eurasian Harm Reduction Network, 2015; Vogus and Graff, 2015; Bennett *et al.*, 2015a; Open Society Foundations, 2017; WHO, 2017; Cernuschi *et al.*, 2018). Such stock-outs could be attributed to difficulties procuring medicines and vaccines at affordable prices following donor transitions (Alkenbrack and Shepherd, 2005; UNAIDS, 2013; Saxenian *et al.*, 2015; Eurasian Harm Reduction Network, 2016a; Cernuschi *et al.*, 2018; Global Fund, 2018). For example, several countries transitioning from Gavi support reported facing challenges with paying the price of directly procuring vaccines themselves vs procuring through Gavi. For some, the cost of direct procurement was nearly twice as high as it was previously, with annual price fluctuations (Saxenian *et al.*, 2015; Cernuschi *et al.*, 2018).

In addition to pricing challenges, some countries have not been able to integrate or sustain donor procurement and supply chain practices during or following donor exits due to inadequate infrastructure and logistics (Alkenbrack and Shepherd, 2005; Curatio International Foundation, 2014; Bliss and Peck, 2016; Eurasian Harm Reduction Network, 2016a; Health Policy Project, 2016a). For example, both Nicaragua and Honduras faced challenges in maintaining and sustaining the quality of their cold chain systems as they proceeded through the Gavi transition process. In both countries, while there was sufficient storage capacity for the newer vaccines at the national-level warehouse, the regional facilities did not have adequate storage capacity for the supplies needed. Lack of storage in sub-national facilities resulted in requests for deliveries two to three times a month, potentially affecting the quality of vaccines (Bliss and Peck, 2016). In addition, some countries experienced supply chain challenges connected to losses in technical capacity for procurement following donor exit. For instance, in Bangladesh, country stakeholders stressed that the government was not prepared to take on the role of procuring anti-retroviral medications following the departure of the Global Fund. Although the Global-Fund-funded Modhumita project—which was focused on integrating HIV services with other essential health programming—was able to create a ‘seamless’ supply chain during the lifespan of the project, as the government took over, lack of technical capacity led to delays in medication procurement and delivery, which affected individuals’ access to medicines (Health Policy Project, 2016a).

To address the challenge of maintaining technical capacity post-transition, some donors have offered technical support or capacity building to such countries throughout the transition process as a means of ensuring a smooth transfer of procurement responsibilities (Coury and Lafebre, 2001; Bertrand, 2011; Kallenberg and Cornejo, 2015; Vogus and Graff, 2015; UNDP, 2017). Where safeguards such as technical capacity and government buy-in were present, countries were able to effectively sustain procurement practices following transition. For example, following its transition from support from Gavi, Albania fully self-financed its domestic vaccination programming, in part due to strong government commitment and budget safeguards for vaccine financing. As a result, the country did not experience any vaccine stock-outs or shortages of injection supplies and even continued to introduce new vaccines such as inactivated poliovirus vaccine and the Rota vaccine without donor support (Curatio International Foundation, 2015). Other countries that had positive experiences during and after donor transitions integrated system-wide changes, such as new policies or regulations, into the transition process (Chaudry *et al.*, 2012; Eurasian Harm Reduction Network, 2016e; 2016f). For example, as a part of its graduation strategy for family planning programmes in Latin America, USAID supported efforts to ensure that national budgets among graduating countries included protected line items for family planning. These line items helped to ensure funding for contraceptives, surgical equipment and other medical and consumable supplies (Shen *et al.*, 2015).

### Information and research

Reliable information plays a significant role in the decision-making process across all health system components. Thus, timely collection, compilation, analysis of information relevant to health systems and research on health and health systems are vital (WHO, 2007). Maintaining and improving information systems relevant to health leading up to the exit of a donor was reported to help countries such as Mexico and Nicaragua with decision-making and needs assessment both during and following the donor exit (Alkenbrack and Shepherd, 2005; Avila *et al.*, 2012). However, other challenges related to funding and human resources can affect the quality of data management and research (Avila *et al.*, 2012; Kazadi, 2015). Lack of proper monitoring systems was seen to negatively affect the assessment of programmes, ultimately leading to a decline in evidence-based decision-making during and after transition due to lack of available data (Curatio International Foundation, 2014; Vogus and Graff, 2015; Dadzie *et al.*, 2017; Cernuschi *et al.*, 2018). For example, inadequate data on immunization in countries such as Bosnia and Herzegovina and Ghana posed challenges in planning and service provision during and after transition (Curatio International Foundation, 2014; Dadzie *et al.*, 2017). In Albania, lack of funding has resulted in a weak post-transition monitoring system for HIV/AIDS programming (Kazadi, 2015).

Where effective monitoring systems are in place, donor and government stakeholders monitor progress and outline targets relevant to programmatic sustainability. For example, when BMGF transitioned its Avahan HIV/AIDS prevention program in India, timely sharing of data and lessons learned between the government and the Avahan program contributed to a smooth transition (Sgaier *et al.*, 2013). In Swaziland, the government took ownership of anti-retroviral supply and used a more reliable quantification methodology, which led to better assessment of needs for anti-retroviral medications thus reducing the cost for the government (UNAIDS, 2013). At the inter-country level, graduating countries have benefited from inter-country exchanges and knowledge-sharing workshops, helping governments to plan and implement vaccine management programmes (Saxenian *et al.*, 2015).

### Service delivery

The reliable delivery of health services and interventions, the final of the six building blocks, is influenced by the other health system components. As an immediate output of all other inputs into the health system (financing, human resources, etc.), service delivery can be characterized by its accessibility, coverage and quality and the efficacy of services provided to the population (WHO, 2007). The publications in this review illustrated that donor transitions can affect service delivery in various ways. Some countries experienced sustained service delivery coverage or even expanded following donor exit (Alkenbrack and Shepherd, 2005; Sgaier *et al.*, 2013; Blanchet and James, 2014; Curatio International Foundation, 2015; Rogers and Coates, 2015; Shen *et al.*, 2015; Vogus and Graff, 2015; Bennett *et al.*, 2015b; Ozawa *et al.*, 2016; TB Europe Coalition, 2016). In Uganda, following the phase-out of PEPFAR support for HIV care via AIDSRelief, each local implementing partner was able to expand its coverage in terms of the number of patients served, geographical reach and services offered (Kazadi, 2015). Ten years after the end of the last Global Fund project in Croatia, the country had sustained the gains of the national HIV response originally achieved with the Global Fund support and expanded many of its components such as testing and counselling. For example, the average number of people tested for HIV almost doubled in the period following the Global Fund’s exit, indicating a scaling up of services after the transition to domestic funding (TB Europe Coalition, 2016). In Ecuador, following USAID’s exit, several family planning NGOs began diversifying the types of services that they offered as a way of generating income and maintaining financial stability. In addition to family planning, these NGOs started to provide other reproductive health services, paediatric care and dental services. These services generated the funds needed to subsidize other services that are not as self-sufficient, such as providing contraceptives for those who live in poverty in rural areas of the country (Coury and Lafebre, 2001).

However, not all country experiences during or following transition were positive. More than a dozen publications in this review described reductions or disruptions in services during or after transition (Blanchet and James, 2014; Cairney and Kapilashrami, 2014; Katz *et al.*, 2014; Kavanagh, 2014; Eurasian Harm Reduction Network, 2015; 2016b; 2016d; 2016f; Republic of Guyana, 2015; Rogers and Coates, 2015; Vogus and Graff, 2015; UNAIDS, 2016; Health Policy Project, 2016a; 2016b; 2016c; 2016d; Biradavolu *et al.*, 2017; Marten, 2017; WHO, 2017; Global Fund, 2018). As NGOs are often used for the delivery of donor-funded services—frequently to vulnerable populations who have difficulty accessing government-sponsored services—they tend to be disproportionately impacted by donor exits. Notably, many NGOs across the globe—including NGOs in China, Thailand, Eastern Europe and Mexico—have reported experiencing challenges sustaining HIV prevention programmes following donor exit (Eurasian Harm Reduction Network, 2015; 2016e; 2016d; 2016b; Health Policy Project, 2016b; Open Society Foundations, 2017; STOPAIDS, 2016; Via Libre, 2017). Where NGOs could no longer provide all services in the absence of donor or government-sponsored support, certain services were prioritized. For example, following its transition from the Swiss Red Cross for eye care interventions, hospitals in a particular district of Ghana maintained certain activities such as outpatient consultations, while discontinuing other activities, such as school outreach (Blanchet and James, 2014). Such reductions or disruptions were often related to insufficient health financing or lack of political will. For example, immunization coverage in Angola and Congo decreased as they were transitioning from Gavi due to stock-outs resulting from inadequate financing (WHO, 2017).

Additional challenges experienced by countries during and after transition included barriers to service access and coverage (Sgaier *et al.*, 2013; Rodríguez *et al.*, 2015; 2017; Rogers and Coates, 2015; TB Europe Coalition, 2016; UNAIDS, 2016; Eurasian Harm Reduction Network, 2016a; 2016b; 2016d; Health Policy Project, 2016c; 2016d; Marten, 2017), a decline in the quality of services provided (Rodríguez *et al.*, 2017) and patient dissatisfaction (Kavanagh, 2014; Rodríguez *et al.*, 2015; Bennett *et al.*, 2015b; Marten, 2017). In Tanzania, reduced PEPFAR support has led to the scale-down of interventions such as food support and transportation reimbursements for patients, which are typically used to increase the uptake of anti-retroviral therapy (ART) and ART adherence (Marten, 2017). A similar experience was documented in Zambia following the withdrawal of support from the World Bank’s Multi-country AIDS Program. Many community-based organizations cited limited funds for transportation and food as their ‘biggest obstacle’ in providing HIV services for orphaned and vulnerable children (Walsh *et al.*, 2012). Both examples highlight how donor exits can have diverse effects on the range of programme components, such as outreach and support services, that are separate from but intimately connected to the successful delivery of health services, particularly to vulnerable populations.

In addition to the impacts on the continuation or disruption of services, donor transitions also led to shifts in the modality of service delivery—either shifting from a centralized system of service delivery to a decentralized system or vice versa—with implications for continuity of care and the continuation of services. For example, as PEPFAR was transitioning out of South Africa it began closing many of its HIV treatment centres and started to move patients into government-run community-based healthcare centres. However, many of these public sector clinics did not or could not hire more staff to deal with the influx of patients. These clinics were poorly prepared to take on this new group of patients. As a result, many patients faced long wait times and shortages of medicine, which contributed to high numbers of patients being lost to follow-up (Katz *et al.*, 2013; Kavanagh, 2014).

### Health outcomes

Although not a building block, one of the best measures of a health system’s performance is health outcomes (WHO, 2007). Information related to health outcomes was rarely reported in the literature on donor transitions within the health sector. When reported, outcomes included those related to HIV and nutrition (Eurasian Harm Reduction Network, 2016a; Open Society Foundations, 2017). For HIV, the reported health outcomes highlight the ways key populations are more vulnerable than other groups during transition (Eurasian Harm Reduction Network, 2016b; Open Society Foundations, 2017). For example, organizations in India, Macedonia and South Africa were able to maintain key HIV performance indicators following transitions away from donor assistance in the immediate period following exit (Kazadi, 2015; Bennett *et al.*, 2015b; Eurasian Harm Reduction Network, 2016f). By contrast, following transitions away from the Global Fund’s assistance for HIV programming, organizations in Romania, Montenegro and Serbia saw increases in HIV infection, resulting from poor political will and critical resource gaps that led to the collapse of service delivery for HIV prevention (Eurasian Harm Reduction Network, 2016b; 2016c; 2016d). Following USAID’s phase-out from nutritional programming in Kenya, Honduras, Bolivia and India, there were mixed results in terms of the impact on nutrition outcomes. Across almost all regions of the four countries, prevalence of child stunting, an indicator of child malnutrition, was sustained or reduced following USAID exit (Rogers and Coates, 2015). However, one region of India and several regions in Kenya saw increases in the prevalence of stunting (Rogers and Coates, 2015). Other impact indicators related to nutrition, such as mothers who continue to feed children during illness, declined sharply following USAID’s exit across all four countries (Rogers and Coates, 2015).

The influence of donor transitions on health outcomes is complex, and there is an important difference between outcomes that are only observed temporarily and outcomes that are sustained over time. A study on USAID’s exit from nutrition programmes found that there was a positive impact on select indicators, such as childhood stunting, where post-transition change was maintained or even improved with time. However, other impact indicators such as feeding the child during illness which looked promising at the time of donor exit—still declined—sometimes significantly—within several years of follow-up (Rogers and Coates, 2015). The mixed results of temporary and sustained outcomes can also be observed related to HIV prevalence and infection rates; whereas countries such as India, Macedonia and South Africa sustained the outcomes improved via donor programmes, other countries such as Romania, Montenegro and Serbia were not able to do so.

## Discussion

Transitions away from DAH can affect the functioning and performance of health systems in unique and varied ways. Through our review, we identified three central observations. First, within the WHO health systems framework, the building blocks of leadership and governance and healthcare financing play a large role in mitigating the potential negative impacts of donor transition, and careful preparation and planning commonly foreshadowed more successful outcomes. Second, most of the publications located through our review are frequently authored by donors themselves and are frequently published as grey literature without a formal review process (e.g. peer review). Third, the literature captured by our review predominantly focuses on transitions in the context of HIV/AIDS and immunization programming, with the majority of the literature discussing transitions from Global Fund, Gavi, USAID and PEPFAR support.

### The importance of leadership, governance and healthcare financing

Based on the literature, leadership and governance and healthcare financing are central to the functionality of health systems, with pressing implications for the success of a given donor transition (Campos and Reich, 2019). Risk factors for poor transition outcomes include lack of clear planning, communication failures, erosion of political will and inadequate legislative and policy infrastructure. Lack of clear planning predominantly impacts key areas such as ensuring sustainable government funding, knowledge transfer and maintaining health workforce stability. As seen in the example of Avahan program transition in India, focusing on strengthening country capacity prior to donor exit led to better policy outcomes. The key success factors stressed during Avahan transition—including early and transparent planning, ongoing communication between donors and governments, and responses to identified challenges—are echoed by other global efforts in graduating from aid (Sgaier *et al.*, 2013). Communication failures include donor–government disconnect and lack of communication with NGOs/CSOs. Additionally, lack of communication between donors in the same country can also contribute to negative results during and after transition. The impact of lack of communication between donors is especially significant during simultaneous donor transitions (i.e. when more than one donor is exiting a country at one time). Therefore, aligning transition plans with country health system strategies remains an important step in donor transitions (Resch and Hecht, 2018).

Lack of political will can arise from the lack of state’s capacity to fund or manage a programme following donor exit. As seen in the Global Fund’s withdrawal from countries such as Romania, this can also be influenced by underlying discriminatory norms that may especially impact programming for marginalized populations (Eurasian Harm Reduction Network 2016b). Prior to and throughout the transition process, donors should evaluate existing political will for sustaining programming following the end of support and assess the impact of withdrawal on specific marginalized populations. To effectively assess political will and potential impact, donors must consult and collaborate with all stakeholders affected by the transition process, including stakeholders from government, NGOs, CSOs and marginalized communities. Inviting this diverse set of groups to provide input on the transition and facilitating intentional engagement and partnerships can be crucial for an effective transition (Resch and Hecht, 2018).

Specific to healthcare financing, major risk factors for negative transition experiences include the inability to secure new sources of funding and/or the mismanagement of funds following donor exit. While the responsibility for managing these risk factors applies to both governments and donors, the majority of the publications in this review focused on financing failures on the part of country governments, rather than financing failures on the part of donors. From a policy perspective, financial sustainability needs to be a key planning item that both governments and donors evaluate and address throughout the stages of a transition. Several transition experiences show that this concerns not just the allocation of funds but also building financial management capacities within the transitioning country (Vogus and Graff, 2015; Calleja and Prizzon, 2019).

While the impact of donor transitions typically commences with effects on leadership, governance and healthcare financing, there are downstream effects that impact all other dimensions of the health system. Notably, we observed that impacts on health financing directly and significantly impact human resources, medicines and technology, and service delivery. Additionally, lack of political will to fill a particular funding gap or communication failures with NGOs/CSOs for service delivery can lead to procurement, service delivery and outreach challenges for health programming. As seen in numerous cases described above, financial gaps in the wake of transition can lead to subsequent cuts in technical capacity, perhaps resulting in commodity stock-outs, which ultimately influences service delivery and health outcomes. As a result, responses from both countries and donors need to consider integrated approaches to ensure the timely identification of interrelated challenges, beginning with leadership, governance and financing considerations. Communication about these challenges must not only be between donor and the government but also with local CSOs, NGOs and other donors. Some organizations have developed transition readiness tools, which seek to assess the sustainability of donor-funded programming following a donor’s departure. While these tools have some limitations, they may still prove useful for transition planning and preparedness efforts (Calleja and Prizzon, 2019; Mao *et al.*, 2021).

### Boundaries and gaps defining current literature on transitions

Considering the literature and reporting process, more attention has been given to donor transitions in the grey literature and little attention has been given to the topic in formal peer-reviewed publications. Most publications included in this review were from sources that are not formally peer-reviewed, including many that were conducted by the donor organizations themselves. While we took the results reported by publications as accurate accounts, the observed bias in both the types of evidence available (e.g. focused on specific health topics) and the sources for that evidence does create concern about what is not being captured within existing scholarship. In addition, we know that the donor landscape is vast, and donor support for programming spans a diverse array of health issues, such as nutrition, polio and eye care programming. And yet, much of the published literature captured in this review examines impacts related to HIV/AIDS and immunization programming, with an overwhelming focus on transitions from the Global Fund and Gavi. This again creates concern about what types of transition experiences are being reported, and whether there is variation in impact based on programme or donor type. Among the publications included in this review, the health system components most frequently discussed were leadership and governance, followed by health financing and service delivery. Less attention has been given to the impact(s) of donor transitions on other components of the health system, such as healthcare workforce and information and research. Health outcomes were rarely discussed in the literature in relation to the influence of donor transitions and warrant additional study, especially considering evidence suggesting that poorly managed donor exits can stall or even reverse the public health gains achieved through DAH-supported services and programming. Additionally, it is also important to note that challenges related to leadership and governance and health financing were most commonly reported due to the high visibility and impact they have on the health system.

### Future directions

Further research should examine which parts of the health system are most vulnerable to transition (i.e. what are the ‘weakest links’) and how impacts on these components affect other components of the health system. Sometimes the evidence on transition is mixed and appears to be context dependent on factors such as the timing of transition initiation, the programme or disease area and whether there was stakeholder buy-in. Given the significant heterogeneity in donor and recipient country priorities, preferences and capacities, there is no ‘one size fits all’ approach to donor transitions. Future research should critically examine whether there are best practices for optimizing outcomes across countries, possibly in relation to a specific donor or specific type of programming. Relatedly, the downstream impact of donor transitions on population health outcomes remains understudied, and future research should empirically examine the impacts donor transitions have on population health. While previous scholars have examined the impacts of donor transitions on specific groups, such as key populations (Health Policy Project, 2016c; Flanagan *et al.*, 2018), future studies may further examine the ways in which donor transitions might disproportionately affect other vulnerable groups, such as the poor, ethnic minorities or populations living in rural settings. While this review excluded the experiences of countries that were in the pre-transition phase, we noted that planning and inclusive communication were framed as necessary elements for facilitating a successful transition. Additional research may seek to examine the links between planning activities occurring prior to donor exit and the subsequent impacts of donor exit on overall health system performance and the implications for health outcomes, particularly in the context of concurrent epidemiological and demographic shifts. To answer these questions, researchers may look to integrate the findings of this review with those of complementary scholarship focused on equitable health systems strengthening, such as existing systematic reviews focused on equity in healthcare financing in MICs (Rostampour and Nosratnejad 2020; Anselmi et al. 2015; Asante et al. 2016), financing strategies and structures for achieving universal health coverage (Darrudi et al. 2022; Ifeagwu et al. 2021; Rizvi et al. 2020; Sanogo et al. 2019) and health systems financing fragmentation, which remains a barrier to achieving health systems goals in MICs (Siqueira et al. 2021; Ahangar et al. 2019).

Throughout the course of this review, we noted several insights that may inform future reviews related to donor transitions. Donor transitions in the health sector are sometimes difficult to define and therefore difficult to systematically review. Consistent with other analyses, we found that different donors take diverse approaches to the withdrawal of financial and technical support, and there is no ‘one size fits all’ approach (McDade *et al.*, 2020). Some donors shift the nature of their aid over time (e.g. from financial to technical support) to facilitate greater country ownership of health programming but do not explicitly label this as a ‘transition’. Since the transition process is understood and defined in different ways across the DAH community including the terminology used across different donors, reporting and disseminating information about the broad impacts of donor transitions can be difficult (McDade *et al.*, 2021). Currently, most of the literature focuses on donor perspectives, limiting our understanding of the impact of donor transitions on MICs. Future analyses focused on country perspectives of donor transition will add value to this research space.

## Strengths and limitations

To the best of our knowledge, this is the first review to examine the extent, range and nature of research activity on the impacts of country transitions away from DAH in MICs. We present findings that have great value to policymakers. We anticipate that our results will be of interest to a wide range of actors involved in the transition process, including donors and policymakers of recipient countries, as well as researchers who study transitions and the impacts of donor transitions. Of note, the specific contexts, health issues and donors varied widely between studies included in our review, and caution should be exercised in generalizing our findings. While our results focused on the impacts of health-related donor transitions, transitions in non-health sectors such as education may still have an impact on domestic health systems and outcomes. Our searches were completed in October 2018, and we were not able to include papers after this time frame in our review. A large volume of evidence came from the grey literature, where methodological considerations were either omitted or vaguely reported. This further prevented us from assessing the potential risk of bias among the publications included in this review. Our results section reflects experiences as they were documented by a given publication, and our results might not be reflective of all perspectives on or dimensions of a given transition experience. Future research may complement findings with key informant interviews to validate the results of a given study. Additionally, our exclusion of non-English publications will have omitted studies published by authors whose primary languages are not English.

## Conclusion

This scoping review sought to identify and describe the literature on the impacts of donor transitions on health systems. Our results show how transitions can affect different components of the health system and population health in both positive and negative ways based on the approaches taken by donors and country governments. In undertaking this review, we also delineated operational definitions of donor transitions, including core conceptual areas and related terms. Given the variation in how transitions are defined both conceptually and practically between stakeholders, our search strategy can provide a valuable framework for future work on donor transitions. Future research priorities include clarifying the relationship between transition planning and the impacts of transitions, as well as empirically examining how the impacts of donor transitions might be inequitably experienced. More rigorous evaluation of the impacts of donor transitions as they relate to health systems performance and outcomes will allow for an improved understanding of the risks and opportunities posed. Thus, optimizing donor transitions in a time that is characterized by concurrent disease and demographic shifts will be imperative to pushing the global health agenda forward.

## Supplementary Material

czac063_Supp

## Reflexivity statement

This project was borne out of a broader body of research led by the Center for Policy Impact in Global Health focused on global health financing. The Center is based out of the Duke Global Health Institute at Duke University in the USA, to which all authors are currently or have previously been affiliated. The paper was developed by a diverse group of authors in terms of citizenship, seniority and gender identity. The team includes persons who are citizens of and have lived experiences in Sri Lanka, the USA, India, China, Iran, Pakistan, Nigeria and the UK. Most authors are early career researchers, and seniority within team ranges from undergraduate to graduate to post-graduate education levels. Most authors identify as women, with the remaining authors identifying as men. During the development of this manuscript, some of the authors were supported by the Bill and Melinda Gates Foundation through a grant that was awarded to the Center for Policy Impact (OPP1199624).
