# Supplementary material for: The impacts of donor transitions on health systems in middle-income countries: a scoping review
Source: Health Policy Plan. 2022 Jul 29;37(9):1188–202. doi: 10.1093/heapol/czac063 (PMC9558870; doi:10.1093/heapol/czac063)
Supplement: czac063_Supp [file czac063_supp.zip › Scoping.appendix.I.30July2021.docx]

Appendix I.

**Table 1. Key term search strategy for academic databases**

| Change | Source | Support | Health | Impact |
| --- | --- | --- | --- | --- |
| Transition  Transitioning  Graduated  Graduating  Decline  Decrease  Sustainability  Withdraw  Departure  Loss  Losing  Phaseout  “Phase out”  “Pull out”  “Exit strategy”  “Country ownership” | Donor  Program  International  External  Foreign  United States  Japan  United Kingdom  PEPFAR  USAID  Gavi  IDA  “International Development Association”  “Gavi the Vaccine Alliance”  “Global Fund”  “World Bank” | Assistance  Aid  Funding  Loan  Finance  Financing  DAH  ODA  Support  “Development assistance for health”  “Official development assistance” | Health  HIV  AIDS  HIV/AIDS  Malaria  TB  Tuberculosis  Vaccine  Vaccination  Immunization  “Family planning”  “Communicable disease”  “Infectious disease” | *Leadership and governance*  Accountability  Governance  Policy  Oversight  Strategy  “Action plan”  *Healthcare financing*  Financing  Expenditure  Budget  “Out-of-pocket”  *Healthcare workforce*  Workforce  Staffing  “Health worker”  “Human resources”  “Technical capacity”  *Medical products/technology*  Procurement  “Supply chain”  “Medical technology”  “Medical products”  “Essential medicines”    *Information and research*  “Information system”  “Health metric”  “Resource tracking”  “Performance indicator”  *Service delivery*  Access  Availability  Coverage  Readiness  Utilization  “Service delivery” |

**Search string: SCOPUS**

TITLE-ABS-KEY ( ( transition  OR  transitioning  OR  graduated  OR  graduating  OR  decline  OR  decrease  OR  sustainability  OR  withdraw  OR  departure  OR  loss OR  OR  losing  OR  phaseout  OR  "Phase out"  OR  "Pull out"  OR  "Exit strategy"  OR  "Country ownership" )  AND  ( donor  OR  program  OR  international  OR  external  OR  foreign  OR  "United States"  OR  japan  OR  "United Kingdom"  OR  pepfar  OR  usaid  OR  gavi  OR  ida  OR  "International Development Association"  OR  "Gavi the Vaccine Alliance"  OR  "Global Fund"  OR  "World Bank" )  AND  ( assistance  OR  aid  OR  funding  OR  loan  OR  finance  OR  financing  OR  dah  OR  oda  OR  support  OR  "Development assistance for health"  OR  "Official development assistance" )  AND  ( health  OR  hiv  OR  aids  OR  hiv/aids  OR  malaria  OR  tb  OR  tuberculosis  OR  vaccine  OR  vaccination  OR  immunization  OR  "Family planning"  OR  "Communicable disease"  OR  "Infectious disease" )  AND  ( accountability  OR  governance  OR  policy  OR  oversight  OR  strategy  OR  "Action plan"  OR  financing  OR  expenditure  OR  budget  OR  "Out-of-pocket"  OR  workforce  OR  staffing  OR  "Health worker"  OR  "Human resources"  OR  "Technical capacity"  OR  procurement  OR  "Supply chain"  OR  "Medical technology"  OR  "Medical products"  OR  "Essential medicines"  OR  "Information system"  OR  "Health metric"  OR  "Resource tracking"  OR  "Performance indicator"  OR  access  OR  availability  OR  coverage  OR  readiness  OR  utilization  OR  "Service delivery" ) )  AND  ( LIMIT-TO ( SRCTYPE ,  "j" ) )  AND  ( LIMIT-TO ( DOCTYPE ,  "ar" ) )  AND  ( LIMIT-TO ( LANGUAGE ,  "English" ) )  AND  ( LIMIT-TO ( PUBYEAR ,  2018 )  OR  LIMIT-TO ( PUBYEAR ,  2017 )  OR  LIMIT-TO ( PUBYEAR ,  2016 )  OR  LIMIT-TO ( PUBYEAR ,  2015 )  OR  LIMIT-TO ( PUBYEAR ,  2014 )  OR  LIMIT-TO ( PUBYEAR ,  2013 )  OR  LIMIT-TO ( PUBYEAR ,  2012 )  OR  LIMIT-TO ( PUBYEAR ,  2011 )  OR  LIMIT-TO ( PUBYEAR ,  2010 )  OR  LIMIT-TO ( PUBYEAR ,  2009 )  OR  LIMIT-TO ( PUBYEAR ,  2008 )  OR  LIMIT-TO ( PUBYEAR ,  2007 )  OR  LIMIT-TO ( PUBYEAR ,  2006 )  OR  LIMIT-TO ( PUBYEAR ,  2005 )  OR  LIMIT-TO ( PUBYEAR ,  2004 )  OR  LIMIT-TO ( PUBYEAR ,  2003 )  OR  LIMIT-TO ( PUBYEAR ,  2002 )  OR  LIMIT-TO ( PUBYEAR ,  2001 )  OR  LIMIT-TO ( PUBYEAR ,  2000 )  OR  LIMIT-TO ( PUBYEAR ,  1999 )  OR  LIMIT-TO ( PUBYEAR ,  1998 )  OR  LIMIT-TO ( PUBYEAR ,  1997 )  OR  LIMIT-TO ( PUBYEAR ,  1996 )  OR  LIMIT-TO ( PUBYEAR ,  1995 )  OR  LIMIT-TO ( PUBYEAR ,  1994 )  OR  LIMIT-TO ( PUBYEAR ,  1993 )  OR  LIMIT-TO ( PUBYEAR ,  1992 )  OR  LIMIT-TO ( PUBYEAR ,  1991 )  OR  LIMIT-TO ( PUBYEAR ,  1990 ) )

**Search string: GLOBAL HEALTH**

( AB ( Transition OR Transitioning OR Graduated OR Graduating OR Decline OR Decrease OR Sustainability OR Withdraw OR Departure OR Loss OR Losing OR Phaseout OR ‘Phase out’ OR ‘Pull out’ OR ‘Exit strategy’ OR ‘Country ownership’ ) AND AB ( Donor OR Program OR International OR External OR Foreign OR ‘United States’ OR Japan OR ‘United Kingdom’ OR PEPFAR OR USAID OR GAVI OR IDA OR ‘International Development Association’ OR ‘Gavi the Vaccine Alliance’ OR ‘Global Fund’ OR ‘World Bank’ ) AND AB ( Assistance OR Aid OR Funding OR Loan OR Finance OR Financing OR DAH OR ODA OR Support OR ‘Development assistance for health’ OR ‘Official development assistance’ ) AND AB ( Health OR HIV OR AIDS OR ‘HIV/AIDS’ OR Malaria OR TB OR Tuberculosis OR Vaccine OR Vaccination OR Immunization OR ‘Family planning’ OR ‘Communicable disease’ OR ‘Infectious disease’ ) AND AB ( Accountability OR Governance OR Policy OR Oversight OR Strategy OR 'Action plan' OR Financing OR Expenditure OR Budget OR 'Out-of-pocket' OR Workforce OR Staffing OR 'Health worker' OR 'Human resources' OR 'Technical capacity' OR Procurement OR 'Supply chain' OR 'Medical technology' OR 'Medical products' OR 'Essential medicines' OR 'Information system' OR 'Health metric' OR 'Resource tracking' OR 'Performance indicator' OR Access OR Availability OR Coverage OR Readiness OR Utilization OR 'Service delivery' ) ) OR ( TI ( Transition OR Transitioning OR Graduated OR Graduating OR Decline OR Decrease OR Sustainability OR Withdraw OR Departure OR Loss OR Losing OR Phaseout OR ‘Phase out’ OR ‘Pull out’ OR ‘Exit strategy’ OR ‘Country ownership’ ) AND TI ( Donor OR Program OR International OR External OR Foreign OR ‘United States’ OR Japan OR ‘United Kingdom’ OR PEPFAR OR USAID OR GAVI OR IDA OR ‘International Development Association’ OR ‘Gavi the Vaccine Alliance’ OR ‘Global Fund’ OR ‘World Bank’ ) AND TI ( Assistance OR Aid OR Funding OR Loan OR Finance OR Financing OR DAH OR ODA OR Support OR ‘Development assistance for health’ OR ‘Official development assistance’ ) AND TI ( Health OR HIV OR AIDS OR ‘HIV/AIDS’ OR Malaria OR TB OR Tuberculosis OR Vaccine OR Vaccination OR Immunization OR ‘Family planning’ OR ‘Communicable disease’ OR ‘Infectious disease’ ) AND TI ( Accountability OR Governance OR Policy OR Oversight OR Strategy OR 'Action plan' OR Financing OR Expenditure OR Budget OR 'Out-of-pocket' OR Workforce OR Staffing OR 'Health worker' OR 'Human resources' OR 'Technical capacity' OR Procurement OR 'Supply chain' OR 'Medical technology' OR 'Medical products' OR 'Essential medicines' OR 'Information system' OR 'Health metric' OR 'Resource tracking' OR 'Performance indicator' OR Access OR Availability OR Coverage OR Readiness OR Utilization OR 'Service delivery' ) )

**Search string: EMBASE**

| #1 | 'transition':ti,ab,kw OR 'transitioning':ti,ab,kw OR 'graduated':ti,ab,kw OR 'graduating':ti,ab,kw OR 'decline':ti,ab,kw OR 'decrease':ti,ab,kw OR 'sustainability':ti,ab,kw OR 'withdraw':ti,ab,kw OR 'departure':ti,ab,kw OR 'loss':ti,ab,kw OR 'losing':ti,ab,kw OR 'phaseout':ti,ab,kw OR 'phase out':ti,ab,kw OR 'pull out':ti,ab,kw OR 'exit strategy':ti,ab,kw OR 'country ownership':ti,ab,kw |
| --- | --- |
| #2 | 'donor':ti,ab,kw OR 'program':ti,ab,kw OR 'international':ti,ab,kw OR 'external':ti,ab,kw OR 'foreign':ti,ab,kw OR 'united states':ti,ab,kw OR 'japan':ti,ab,kw OR 'united kingdom':ti,ab,kw OR 'pepfar':ti,ab,kw OR 'usaid':ti,ab,kw OR 'gavi':ti,ab,kw OR 'ida':ti,ab,kw OR 'international development association':ti,ab,kw OR 'gavi the vaccine alliance':ti,ab,kw OR 'global fund':ti,ab,kw OR 'world bank':ti,ab,kw |
| #3 | 'assistance':ti,ab,kw OR 'aid':ti,ab,kw OR 'funding':ti,ab,kw OR 'loan':ti,ab,kw OR 'finance':ti,ab,kw OR 'financing':ti,ab,kw OR 'dah':ti,ab,kw OR 'oda':ti,ab,kw OR 'support':ti,ab,kw OR 'development assistance for health':ti,ab,kw OR 'official development assistance':ti,ab,kw |
| #4 | 'health':ti,ab,kw OR 'hiv':ti,ab,kw OR 'aids':ti,ab,kw OR 'hiv/aids':ti,ab,kw OR 'malaria':ti,ab,kw OR 'tb':ti,ab,kw OR 'tuberculosis':ti,ab,kw OR 'vaccine':ti,ab,kw OR 'vaccination':ti,ab,kw OR 'immunization':ti,ab,kw OR 'family planning':ti,ab,kw OR 'communicable disease':ti,ab,kw OR 'infectious disease':ti,ab,kw |
| #5 | 'accountability':ti,ab,kw OR 'governance':ti,ab,kw OR 'policy':ti,ab,kw OR 'oversight':ti,ab,kw OR 'strategy':ti,ab,kw OR 'action plan':ti,ab,kw OR 'financing':ti,ab,kw OR 'expenditure':ti,ab,kw OR 'budget':ti,ab,kw OR 'out-of-pocket':ti,ab,kw OR 'workforce':ti,ab,kw OR 'staffing':ti,ab,kw OR 'health worker':ti,ab,kw OR 'human resources':ti,ab,kw OR 'technical capacity':ti,ab,kw OR 'procurement':ti,ab,kw OR 'supply chain':ti,ab,kw OR 'medical technology':ti,ab,kw OR 'medical products':ti,ab,kw OR 'essential medicines':ti,ab,kw OR 'information system':ti,ab,kw OR 'health metric':ti,ab,kw OR 'resource tracking':ti,ab,kw OR 'performance indicator':ti,ab,kw OR 'access':ti,ab,kw OR 'availability':ti,ab,kw OR 'coverage':ti,ab,kw OR 'readiness':ti,ab,kw OR 'utilization':ti,ab,kw OR 'service delivery':ti,ab,kw |
| #6 | #1 AND #2 AND #3 AND #4 AND #5 AND [article]/lim AND [english]/lim AND [1990-2018]/py |

**Search string: PAIS**

noft(Transition OR Transitioning OR Graduated OR Graduating OR Decline OR Decrease OR Sustainability OR Withdraw OR Departure OR Loss OR Losing OR Phaseout OR ‘Phase out’ OR ‘Pull out’ OR ‘Exit strategy’ OR ‘Country ownership’) AND noft(Assistance OR Aid OR Funding OR Loan OR Finance OR Financing OR DAH OR ODA OR Support OR ‘Development assistance for health’ OR ‘Official development assistance’) AND noft(Donor OR Program OR International OR External OR Foreign OR ‘United States’ OR Japan OR ‘United Kingdom’ OR PEPFAR OR USAID OR GAVI OR IDA OR ‘International Development Association’ OR ‘Gavi the Vaccine Alliance’ OR ‘Global Fund’ OR ‘World Bank’) AND noft(Health OR HIV OR AIDS OR ‘HIV/AIDS’ OR Malaria OR TB OR Tuberculosis OR Vaccine OR Vaccination OR Immunization OR ‘Family planning’ OR ‘Communicable disease’ OR ‘Infectious disease’) AND noft(Accountability OR Governance OR Policy OR Oversight OR Strategy OR 'Action plan' OR Financing OR Expenditure OR Budget OR 'Out-of-pocket' OR Workforce OR Staffing OR 'Health worker' OR 'Human resources' OR 'Technical capacity' OR Procurement OR 'Supply chain' OR 'Medical technology' OR 'Medical products' OR 'Essential medicines' OR 'Information system' OR 'Health metric' OR 'Resource tracking' OR 'Performance indicator' OR Access OR Availability OR Coverage OR Readiness OR Utilization OR 'Service delivery')

**Search string: POLITICAL SCIENCE COMPLETE**

( AB ( Transition OR Transitioning OR Graduated OR Graduating OR Decline OR Decrease OR Sustainability OR Withdraw OR Departure OR Loss OR Losing OR Phaseout OR ‘Phase out’ OR ‘Pull out’ OR ‘Exit strategy’ OR ‘Country ownership’ ) AND AB ( Donor OR Program OR International OR External OR Foreign OR ‘United States’ OR Japan OR ‘United Kingdom’ OR PEPFAR OR USAID OR GAVI OR IDA OR ‘International Development Association’ OR ‘Gavi the Vaccine Alliance’ OR ‘Global Fund’ OR ‘World Bank’ ) AND AB ( Assistance OR Aid OR Funding OR Loan OR Finance OR Financing OR DAH OR ODA OR Support OR ‘Development assistance for health’ OR ‘Official development assistance’ ) AND AB ( Health OR HIV OR AIDS OR ‘HIV/AIDS’ OR Malaria OR TB OR Tuberculosis OR Vaccine OR Vaccination OR Immunization OR ‘Family planning’ OR ‘Communicable disease’ OR ‘Infectious disease’ ) AND AB ( Accountability OR Governance OR Policy OR Oversight OR Strategy OR 'Action plan' OR Financing OR Expenditure OR Budget OR 'Out-of-pocket' OR Workforce OR Staffing OR 'Health worker' OR 'Human resources' OR 'Technical capacity' OR Procurement OR 'Supply chain' OR 'Medical technology' OR 'Medical products' OR 'Essential medicines' OR 'Information system' OR 'Health metric' OR 'Resource tracking' OR 'Performance indicator' OR Access OR Availability OR Coverage OR Readiness OR Utilization OR 'Service delivery' ) ) OR ( TI ( Transition OR Transitioning OR Graduated OR Graduating OR Decline OR Decrease OR Sustainability OR Withdraw OR Departure OR Loss OR Losing OR Phaseout OR ‘Phase out’ OR ‘Pull out’ OR ‘Exit strategy’ OR ‘Country ownership’ ) AND TI ( Donor OR Program OR International OR External OR Foreign OR ‘United States’ OR Japan OR ‘United Kingdom’ OR PEPFAR OR USAID OR GAVI OR IDA OR ‘International Development Association’ OR ‘Gavi the Vaccine Alliance’ OR ‘Global Fund’ OR ‘World Bank’ ) AND TI ( Assistance OR Aid OR Funding OR Loan OR Finance OR Financing OR DAH OR ODA OR Support OR ‘Development assistance for health’ OR ‘Official development assistance’ ) AND TI ( Health OR HIV OR AIDS OR ‘HIV/AIDS’ OR Malaria OR TB OR Tuberculosis OR Vaccine OR Vaccination OR Immunization OR ‘Family planning’ OR ‘Communicable disease’ OR ‘Infectious disease’ ) AND TI ( Accountability OR Governance OR Policy OR Oversight OR Strategy OR 'Action plan' OR Financing OR Expenditure OR Budget OR 'Out-of-pocket' OR Workforce OR Staffing OR 'Health worker' OR 'Human resources' OR 'Technical capacity' OR Procurement OR 'Supply chain' OR 'Medical technology' OR 'Medical products' OR 'Essential medicines' OR 'Information system' OR 'Health metric' OR 'Resource tracking' OR 'Performance indicator' OR Access OR Availability OR Coverage OR Readiness OR Utilization OR 'Service delivery' ) OR (KW ( Transition OR Transitioning OR Graduated OR Graduating OR Decline OR Decrease OR Sustainability OR Withdraw OR Departure OR Loss OR Losing OR Phaseout OR ‘Phase out’ OR ‘Pull out’ OR ‘Exit strategy’ OR ‘Country ownership’ ) AND KW ( Donor OR Program OR International OR External OR Foreign OR ‘United States’ OR Japan OR ‘United Kingdom’ OR PEPFAR OR USAID OR GAVI OR IDA OR ‘International Development Association’ OR ‘Gavi the Vaccine Alliance’ OR ‘Global Fund’ OR ‘World Bank’ ) AND KW ( Assistance OR Aid OR Funding OR Loan OR Finance OR Financing OR DAH OR ODA OR Support OR ‘Development assistance for health’ OR ‘Official development assistance’ ) AND KW ( Health OR HIV OR AIDS OR ‘HIV/AIDS’ OR Malaria OR TB OR Tuberculosis OR Vaccine OR Vaccination OR Immunization OR ‘Family planning’ OR ‘Communicable disease’ OR ‘Infectious disease’ ) AND KW ( Accountability OR Governance OR Policy OR Oversight OR Strategy OR 'Action plan' OR Financing OR Expenditure OR Budget OR 'Out-of-pocket' OR Workforce OR Staffing OR 'Health worker' OR 'Human resources' OR 'Technical capacity' OR Procurement OR 'Supply chain' OR 'Medical technology' OR 'Medical products' OR 'Essential medicines' OR 'Information system' OR 'Health metric' OR 'Resource tracking' OR 'Performance indicator' OR Access OR Availability OR Coverage OR Readiness OR Utilization OR 'Service delivery' ) )
