# Supplementary material for: The impacts of donor transitions on health systems in middle-income countries: a scoping review
Source: Health Policy Plan. 2022 Jul 29;37(9):1188–202. doi: 10.1093/heapol/czac063 (PMC9558870; doi:10.1093/heapol/czac063)
Supplement: czac063_Supp [file czac063_supp.zip › Scoping.appendix.II.30July2021.docx]

Appendix II.

**Table 1. Key term search strategy for gray literature**

| **Change** | **Source** | **Support** | **Health** | **Impact** |
| --- | --- | --- | --- | --- |
| 1. Transition 2. Graduat* 3. Decline 4. Sustainability 5. Withdraw 6. Phaseout 7. Loss 8. Exit 9. “Country ownership” | 1. Donor 2. Program 3. International 4. External 5. Foreign | 1. Assistance 2. Aid 3. Funding 4. Loan 5. Financ* 6. Support | 1. Health 2. HIV 3. Malaria 4. Tuberculosis 5. Vaccin* 6. “Family planning” 7. Disease | 1. Effect 2. Impact 3. Experience |

**Table 2. List of websites searched for gray literature**

| Websites searched | | | | |
| --- | --- | --- | --- | --- |
| Acesco Global | DFID | MFAN | R4D | Inst. for Dev’t Studies |
| ACTION | Gavi | MSF | THET |  |
| Aidspan | GPEI | OSF | Thinkwell |  |
| BMGF | Global Fund | ODI | UNAIDS |  |
| CARE | HPP | Pharos | UNDP |  |
| CGD | ICASO | PAHO | UNFPA |  |
| CSIS | IDA | PANCAP | USAID |  |
| CFS | Independent Commission for Aid Impact | PEPFAR | WHO |  |
| Curatio | Learning Network for Countries in Transition | Results UK | World Bank |  |
