# Supplementary material for: The impacts of donor transitions on health systems in middle-income countries: a scoping review
Source: Health Policy Plan. 2022 Jul 29;37(9):1188–202. doi: 10.1093/heapol/czac063 (PMC9558870; doi:10.1093/heapol/czac063)
Supplement: czac063_Supp [file czac063_supp.zip › Scoping.appendix.III.Clean.27July2022.docx]

Appendix III.

**Table 1. Impact of transitions away from DAH on leadership and governance systems in recipient countries**

| **Health issue** | **Donors** | **Findings** |
| --- | --- | --- |
| Family planning | USAID | *Coury and Lafebre (2001) – Ecuador*   - In preparation for transition, the Ministry of Health in Ecuador, together with public and private stakeholders, formed an inter-institutional committee on contraceptive security. In addition, local service delivery organizations were restructured to become more market-oriented to enable them face transition effectively.   *USAID and the Ministry of Health of Kingdom of Morocco (2003) – Morocco*   - There was strong political commitment and consistent donor support, resulting in robust support for key maternal and child health programs through critical periods of program growth. This facilitated government takeover of contraceptive purchasing in Morocco. - Advocacy and national scale-up workshops were held in Morocco to secure government buy-in to take over the financial obligations for the purchase of contraceptives.   *Sine et al. (2004) – Turkey*   - Stakeholders had to manage the transition process in a unique political and institutional context. - The Ministry of Health in Turkey overcame challenges to obtain new resources to replace donated contraceptive commodities, assume new technical responsibilities.   *Alkenbrack and Shepherd (2005) – Mexico*   - Stakeholders created coordinating bodies and facilitated agreements between government and non-governmental institutions to coordinate phase-out of USAID funds. This included the development of a five-year transition strategy for non-profits and the government. - Intersectoral collaboration during transition was weak creating missed opportunities for government and non-government organizations to work together in important areas like procurement etc. - Weak communication and collaboration between levels of government (national and state level) resulted in avoidable implementation delays. - Transition was complicated by mixed signals from USAID about timing of phase-out, and by lack of clarity of the role of non-governmental organizations in the transition strategy.   *Bertrand (2011) – Multiple countries (Paraguay, Honduras, Nicaragua, and Peru)*   - Paraguay, Honduras, and Nicaragua all developed plans to take ownership of their family planning programs following the withdrawal of aid, but achieved differing levels of success. - At the beginning of the process, all three countries were initially hesitant to believe that USAID would actually withdraw aid.   *USAID (2013) – Paraguay*   - Paraguay’s experience establishing and strengthening a national contraceptive security committee serves as a success story for improving reproductive health indicators in a context of increased country ownership. Paraguay, via its consecutive strategic plans, created a supportive policy environment for family planning, leading to increased financing and political commitment that has made contraceptive supplies more easily available.   *Shen et al. (2015) – Multiple countries (Dominican Republic, Egypt, El Salvador, Honduras, Jamaica, Kazakhstan, Nicaragua, Paraguay, Peru, Ukraine, and Uzbekistan)*   - Contraceptive Security Committees, initiated by USAID, aimed to address long term product needs, reduce duplication, and improve programmatic efficiency within transitioning countries. Some countries such as Dominican Republic, Nicaragua have officially recognized the Contraceptive Security Committee following graduation from USAID support. - USAID’s efforts led countries such as Nicaragua to pass laws to codify policy on family planning including budget lines and to provide family planning essentials within a country’s public health services. - Across countries, the Contraceptive Security Committees raised awareness and helped implement family planning graduation plans by designing national strategies and annual work plans aimed at achieving contraceptive security and making contraceptive security a priority.   *Avila et al. (2016) - Nicaragua*   - There was strong political commitment from the government to develop a phased-approach (health system strengthening and sustainability development phases) which resulted in capacity building and a successful implementation of the graduation strategy.   *Chaudry et al. (2012) – Multiple countries (South Korea, Panama, Sri Lanka, Tunisia, Botswana, Thailand, Costa Rica, Swaziland, Colombia, Mexico, Brazil. Ecuador, Morocco, Turkey, Indonesia, Romania, Jamaica, Dominican Republic, South Africa, El Salvador, Paraguay, Nicaragua, Honduras, Peru, Albania, Ukraine, Egypt, Bangladesh, Zimbabwe (Specific examples from Dominican Republic, Ecuador, El Salvador, Honduras, Indonesia, Jamaica, Mexico, Nicaragua, Paraguay, and Peru)*   - Government leadership and commitment to program sustainability was a prerequisite for all successful graduations examined (e.g. Mexico and Paraguay). - In countries where graduations occurred in the context of broad decentralization, graduation strategies tended to focus more on technical assistance to regional and district-level governance structures to increase their capacity (e.g. such as in Honduras, Indonesia, Paraguay, and Peru). - USAID pushed for major policy reforms by providing financial and other incentives (e.g. El Salvador, Nicaragua, Paraguay, and the Dominican Republic, Ecuador). This led to the formation contraceptive security committees. - Engagement with various ministries (i.e., health, education, social security, youth, AIDS programming, protection services, etc.) via the creation of national committees and working groups ensured that comprehensive policies were in place to maintain quality and access to services (e.g. Dominican Republic, Jamaica, and Nicaragua). - Health sector commodity hand-over strategies were most clear and successful in contexts in which the national government provided specific line-item funding and/or there was a broad mix of non-governmental and civil society organizations contributing to financing the commodity supply, distribution, and legal and policy frameworks over a sustained period of time. - In the case of Ecuador, under the “no project assistance relationship” in 1991, USAID provided large sums for financial support in exchange for major national policy reforms, which helped to create an enabling regulatory environment. - Other key success factors among countries examined include: endowments for institutions with proven capacity, rational planning to phase out programs, institutional strengthening, and identifying target champions among government, civil society, non-governmental organizations for handover. |
| Immunization | Gavi, the Vaccine Alliance | *Levin et al. (2010) – Multiple countries (Armenia, Azerbaijan, Afghanistan, Burundi, Bhutan, Albania, Cambodia, Burkina Faso, Angola, Djibouti, Cameroon, Bangladesh, Ethiopia, Central African Republic, Bolivia, Gambia, Comoros, Chad, Georgia, Democratic Republic of Congo, China, Democratic Republic of Korea, Ghana, Congo, Lao People's Democratic Republic, Indonesia, Eritrea, Senegal, Kenya, Guinea, Sierra Leone, Lesotho, Haiti, Sudan, Mali, Honduras, Uganda, Mozambique, Republic of Kyrgyz, Yemen, Myanmar, Mauritania, Zambia, Nepal, Niger, Pakistan, Sri Lanka, Rwanda, Tajikistan, Sao Tome, Turkmenistan, Somalia, Ukraine, Tanzania, Vietnam, Togo, Zimbabwe, and Uzbekistan)*   - As a result of strong political commitment by countries since the start of the program in 2002, 25 of 58 Gavi’s injection-safety support recipient countries have transitioned to purchasing their own syringes and safety boxes with government funding.   *Gavi (2013) – Mongolia*   - As a result of transition, the government of Mongolia committed to fully funding routine immunization and contributed more than the required amount for co-financing pentavalent vaccine procurement.   *Gavi (2014) – Bosnia and Herzegovina*   - Stakeholders on Bosnia and Herzegovina created a post-transition coordination function for the immunization program. This post-transition coordination function was however not effective. As a result, countrywide planning and budgeting became crippled in post-Gavi transition period.   *Gavi (2015) – Albania*   - In the pre- and post-transition periods, strong political support and national immunization program coordination in Albania contributed to the continued success of the national immunization program.   *Kallenburg and Cornejo (2015) – Countries transitioning out of Gavi support after 2015*   - Lessons from several countries suggest that lack of clarity on graduation eligibility and transition timelines affect planning at the country level. Civil society organizations were found to be vital contributors successful transition at the community level.   *Saxenian et al. (2015) – Multiple countries (Bhutan, Republic of Congo, Georgia, Moldova, and Mongolia)*   - Some countries established National Immunization Technical Advisory Groups (NITAG) to advise the government on evidence-based policies and program options (e.g. Mongolia).   *Kallenburg et al. (2016) – Multiple countries (Angola, Armenia, Azerbaijan, Bhutan, Bolivia, Republic of the*  *Congo, Cuba, Georgia, Guyana, Honduras, Indonesia, Kiribati, Moldova, Mongolia, Sri Lanka, Timor-Leste, Ukraine, Uzbekistan, and Vietnam)*   - Although many countries in accelerated transition status built strong immunization programs and mobilized domestic resources, some countries struggled to sustain immunization programs via domestic funding.   *Bliss and Peck (2016) – Multiple countries (Honduras and Nicaragua)*   - In Honduras, Gavi co-financing requirements led to close coordination between the health and finance secretariats. - In Nicaragua, the government signed an agreement with Russia to strengthen domestic regulation and production of biological products including vaccines.   *Kerr (2017) – Multiple countries (Nepal, Nigeria, Sri Lanka, and Uganda)*   - The government took ownership during transition, prioritizing immunization policy, immunization financing, and within-government collaborations (e.g., Sri Lanka, Uganda, and Nepal). - In some cases (e.g. Nigeria) new agencies were established to increase government commitment and funding targeted at transition.   *Almatov et al. (2017) – Uzbekistan*   - Government showed commitment to transition via funding, new construction projects, and participating in procurement and distribution. - Government introduced new standard operating procedures for the use of vaccine formulations and refrigeration equipment in storage warehouses.   *Dadzie et al. (2017) – Ghana*   - Ghana aimed to have a functional National Immunization Technical Advisory Group (NITAG) and in its absence Inter Agency Co-ordinating Council (ICC) advised ministry of health on policy and programmatic issues related to immunization.   *Results for Development (2017) – Multiple countries (Armenia, the Republic of the Congo, Georgia, Ghana, Indonesia, the Lao People’s Democratic Republic, Moldova, Sri Lanka, Sudan, Timor-Leste, Uzbekistan, and Vietnam)*   - Countries ensured government commitment by holding key meetings to ensure funding, justifying from an economic perspective, showing high performance and benefits of the immunization program, showing reduction in hospitalization for vaccine preventable diseases.   *Results for Development (2017) – Multiple countries (Armenia, Azerbaijan, Bhutan, Costa Rica, Ghana, Indonesia, Kenya, and Sri Lanka)*   - Government funding commitment (e.g. Bhutan), and strong political commitment and information sharing between finance and health ministries (e.g. Armenia) was beneficial during transition.   *Teixeira et al. (2017) –* *Multiple countries (Côte d’Ivoire, Nigeria, and Vietnam)*   - Nigeria showed little evidence of concrete actions to increase technical capacity and funding once the Global Polio Eradication Initiative (GPEI) and Gavi support ends. - The National Primary Health Care Development Agency and the National Immunization Financing Task Team conducted analysis and advocacy activities to address the lack of action by the government.   *Cernuschi et al. (2018) –* *Multiple countries (Angola, Armenia, Azerbaijan, Bhutan, Bolivia, Congo Republic, Georgia, Ghana, Guyana, Honduras, Moldova, Mongolia, Papua New Guinea, Sri Lanka, and Uzbekistan)*   - Despite most transitioning countries progressing toward establishing National Immunization Technical Advisory Groups (NITAGs) in line with Global Vaccine Action Plan 2011-2020 targets, 60% of the countries studied lacked a functional NITAG. - African countries were more likely to not have a functioning NITAG. |
| HIV/AIDS and tuberculosis | Global Fund to fight AIDS Tuberculosis and Malaria (Global Fund)  Bill and Melinda Gates Foundation (BMGF)  Presidents Emergency Plan for AIDS Relief (PEPFAR) | *Torpey et al. (2010) – Zambia*   - Technical assistance was replaced by management plans developed with provincial and district health offices to maintain programmatic and technical sustainability.   *Walsh et al. (2012) – Zambia*   - Lessons from Zambia National Response to HIV/AIDS (ZANARA) project, which was to run between 2003 and 2008: One component of ZANARA was the Community Response to AIDS (CRAIDS). CRAIDS funded projects (e.g. effectiveness of community health workers) were incorporated into Zambia’s National Community Health Worker Strategy.   *Sgaier et al. (2013) – India*   - Transition was facilitated through alignment of priorities of the Bill and Melinda Gates Foundation and the Government of India. - Use of evidence-based planning helped increase political commitment to fight HIV/AIDS.   *UNAIDS (2013) – Multiple countries (Swaziland, Namibia, and Kazakhstan)*   - In Swaziland, the government worked with partners to develop and implement procedures to strengthen the tender process, achieve low prices such as price benchmarking, evaluating supplier performance, improving the reliability of estimates and increasing the efficiency of the process. The government also worked to ensure that robust tender practices were institutionalized within the Ministry of Health as part of its long-term procurement strategy for antiretrovirals and other essential medicines. - In Namibia, the National Strategic Framework (2010/11 -2015/16 ) was aligned with the outcomes of the UN 2011 political declaration on HIV/AIDS with targets costed and domestic funding contribution identified for each target. In August 2010, the President directed the Ministry of Health and Social Services to consult Ministry of Finance and other stakeholders to develop proposals to achieve sustainable financing for HIV in the country. - In Kazkhastan the government through its state health program “Salamatty Kazakhastan” aims to engage non-governmental organizations (NGOs) and businesses in health promotion from 2011-2015, setting up different funds to build capacity of NGOs working in prison, AIDS-service NGOs etc.   *Global Fund (2013) –* *Multiple countries (Algeria, Argentina, China, Croatia, Equatorial Guinea, Estonia, Jamaica, Romania, Russian Federation, South Africa, Thailand, and Ukraine)*   - Coordination and implementation structures used to support transition and sustainability differ by country. Technical task teams in South Africa, National AIDS Control Council in Kenya, and the National Health Program in Jamaica. - Lessons from successful planning to sustain Global Fund in transition settings in Jamaica and Estonia show that the transition process requires participation of all stakeholders to identify strategies.   *Amaya et al. (2014) – Peru*   - Decision making was concentrated at the national level with less input from regional leaders and other ministries outside the Ministry of Health. - There was a lack of a multisectoral response from the government. - Rapid decentralization without proper preparation affected the capacity of actors to respond to transition.   *Katz et al. (2014) – Kenya*   - At the end of 2012 Kenyan Cabinet approved a bill “Additional and Sustainable Financing for HIV/AIDS and Non-Communicable Diseases in Kenya” proposing 1% of tax revenue allocated to a Trust Fund to sustain post-transition financing.   *Kavanagh (2014) – South Africa*   - Despite agreeing in the Partnership Framework, PEPFAR failed to ensure continued anti-retroviral therapy (ART) and pre-ART care during transition due lack of systemic plan or PEPFAR funded capacity to track patient care.   *UNDP (2014) –El Salvador*   - In 2007, in El Salvador a gradual process of transitioning responsibility for Global Fund grant management and implementation to national entities began, with the Ministry of Health becoming co-Principal Recipients of both HIV and TB grants, along with UNDP. However, there were weaknesses in the procurement system allowing only a partial transfer of procurement responsibilities from UNDP. - From 2007-2011 capacity building by the UNDP led to establishing a Global Fund Management Unit in the Ministry of Health of El Salvador   *Vogus and Graff 2015 – Countries graduated from PEPFAR by 2013 in Latin America, Caribbean and countries under transition from PEPFAR in 2013*   - Lack of a written plan and clear communication about PEPFAR transition created resentment and frustration among South African officials and skepticism among civil society organizations. - Considering political and economic factors remain crucial in transition. For example, Mexico’s decentralization changed funding needs, but the transition plan was not flexible enough to accommodate this change. - Policies are needed to protect the rights of individuals to access essential services as learnt through USAID family planning graduation program experience.   *Bennet et al. (2015) – India*   - The transition of programming from non-governmental organizations to the government of India created initial management issues (during first phase of transition) that were used to inform subsequent phases of the transition process. - There was political support and political will to support and address gaps identified through the transition process to ensure programmatic success.   *Bennet et al. (2015b) – India*   - Avahan transition addressed issues identified in first round of transition. These included improving communication, commitment, better role clarity and joint leadership between stakeholders with better results in latter rounds.   *Eurasian Harm Reduction Network (2015) – Bulgaria*   - Harm reduction was not considered a top political priority and that negatively affected harm reduction HIV prevention in Bulgaria post-transition.   *Eurasian Harm Reduction Network (2015) – Serbia*   - The Serbian Government articulated support for HIV/AIDS but without a financial commitment. - Poor coordination with other donors after Global Fund’s withdrawal negatively impacted the ability to finance prevention program for key populations. - Serbia lacked an institutional framework to lead and coordinate the HIV response post-transition; HIV prevention programs among key populations in the national strategy remained “on paper” due to lack of allocated funding.   *Kazadi (2015) – Multiple countries (Uganda, Kenya, Tanzania and South Africa)*   - Planning for transition from the beginning of donor funded programs with local partners resulted in successful transitions and local ownership with specific safeguards for service delivery (e.g. Uganda, Kenya, Tanzania, and South Africa) - Clear communication, commitment to transition and role clarity between donor, AIDS relief consortium members and partners led to better transition experiences.   *Rodriguez et al. (2015) – India*   - Although in initial rounds key population and front-line staff were not informed of transition, in later rounds staff and other stakeholders were informed of the transition. - In Tamil Nadu, India, the State lead partner played a vital role in ensuring transition plans were communicated in advance to all stakeholders.   *UNAIDS Program Coordinating Board Meeting (2015) – Multiple countries (Kazakhstan, Namibia, India, and Thailand)*   - Kazakhstan’s AIDS response adopted an integrated approach, prioritizing scale-up of HIV treatment, prevention programs for key populations and partnerships with civil society. - In Namibia, as international development partners reduced their support for the AIDS response, the government took steps to increase domestic financing for AIDS. As part of its effort to develop a clear national plan for financial sustainability of the response, Namibia conducted a financial analysis, fiscal space and cost effectiveness study of health and AIDS programs, with a plan to use results to inform an investment framework later in 2015. - In India, the AIDS response has undergone a paradigm shift, transitioning from a donor-driven response to one that is owned and largely financed by India itself. The prevailing national program in India aimed to reduce new HIV infections and provide comprehensive treatment, care and support for people living with HIV, with the overarching goal of having a balanced response that maintains an important emphasis on primary prevention. - Thailand, anticipating the end of Global Fund support, civil society organizations formed an alliance and activated mechanisms to mobilize resources from the public and private sectors. The alliance was collectively financed and managed as a partnership, with technical support provided by UNAIDS.   *UNAIDS Program Coordinating Board Meeting (2016) – For countries receiving funding from Global Fund countries*   - As Global Fund was leaving eastern Europe and central Asia, most countries in transition or post-transition did not have proper mechanisms to replace Global Fund resources with domestic funding, especially for HIV prevention programs by communities. Most of the domestic funding was allocated to treatment provision, rather than prevention efforts. - Simultaneous transitions were a problem as other donors also reduced their budgets for the same activities/programs at the same time as Global Fund.   *Rivalan (2016) – Serbia*   - Following transition since 2015, Serbia’s high HIV prevalence indicates a failed transition process and could make the country eligible for Global Fund support. - Serbia’s experience demonstrates that halting international funding without genuine and sustained support during the transition period can destroy results gained through prior investment and affect progress in HIV prevention and care.   *Burrows et al. (2016) – Multiple countries (specific example from Bulgaria)*   - In Bulgaria the sudden departure of Global Fund threatened the sustainability of HIV/AIDS response. For example, a Bulgaria 2016-20 strategic plan was not drafted.   *Eurasian Harm Reduction Network (2016) – Montenegro*   - When Global Fund support ended in 2015, the government of Montenegro was not ready to lead and fund its domestic HIV response. As a result, by the end of 2015, the government had passed an amendment to fund non-governmental organizations to carry out domestic HIV programming, but the long-term sustainability of this funding is unclear.   *Eurasian Harm Reduction Network (2016c) – Romania*   - Lack of political will resulted in barriers for service delivery for harm reduction in post-transition settings such as lack of strategic HIV framework since 2007, lack of political will to publicly address increase of HIV among men who have sex with men, lack of targeting by the national HIV program on prevention measures for most affected key populations. - Existing governance structures were not effective after the donor departed due to a failure to institutionalize previous structures. - Lack of planning both from the Romanian government and Global Fund resulted in poor transition of HIV programs, especially programs implementing harm reduction. - In contrast Romania’s TB program’s costed transition plan resulted in sustained programming post-transition, thereby emphasizing the importance of political will and donor support.   *Eurasian Harm Reduction Network (2016) – Macedonia*   - In Macedonia, the transition plan was only endorsed at the level of ministry of health. It was not endorsed by the government or the ministry of finance. This affected transition efforts.   *Eurasian Harm Reduction Network (2016) – Albania*   - There was no planning to institutionalize the Country Coordinating Mechanism as a national governance body with decision making power for HIV programming by the government or other stakeholders. - Lack of political will led to degradation of services in the post transition period.   *Health Policy Project (2016) – Bangladesh*   - Main challenges are lack of government ownership and capacity to take over existing programs, lack of support for civil society organizations, poor communication by donors on transition ending, and lack of planning for post-transition absorption of responsibilities. All these negatively impacted the success of transition efforts.   *Health Policy Project (2016) – China*   - Civil society organizations (CSOs) are required to register with a government management partner in China which exerts significant control over programming and finances. CSOs were generally reluctant to apply to social service outsourcing financing mechanism citing threat to their autonomy.   *TB Europe Coalition (2016) – Multiple countries (Azerbaijan, Croatia, Serbia, Kyrgyzstan, Romania, and Ukraine)*   - Croatia had a well-institutionalized national HIV response mechanism with established governance structures and strong civil society involvement, which occurred much earlier than transition dates. - Azerbaijan assumed provision of first-line TB drugs after the departure of Global Fund in 2011, and in 2015 100% of second line drugs were procured by the government budget. - Serbian government, despite pledges to continue harm reduction services only supported a small proportion of programs.   *UNAIDS (2016) – Argentina*   - Prior to transition, the Argentinian Ministry of Health’s directorate for HIV and sexually transmitted infections began funding community organizations and networks directly, opening doors for collaboration with municipal and regional governments.   *TB Europe Coalition (2017) – Bulgaria*   - Following transition in Bulgaria, continuing to contract non-governmental organizations through the government faced challenges due to legal hurdles and low domestic resource allocation.   *Marten (2017) – Tanzania*   - There were concerns about accountability, responsibility and coordination among donors and governments. Stakeholders indicated transitioning program responsibilities from international organizations to local organizations would be an interim phase that ideally would result in incorporation to the government system.   *Open Society Foundation (2017) – Multiple countries (Macedonia, Montenegro, and Serbia)*   - In Macedonia:   - Most of the non-governmental programming previously supported by the Global Fund was supposed to be included in the Ministry of Health budget during the no-cost extension period, but remained unfunded (since July 2017) due to the lack of a functioning mechanism for the government to contract with non-governmental organizations (NGOs).   - The National HIV Strategy for 2017-2021 was built around maintaining service levels in the absence of external support. Under the latest draft, up to 54% of the budget for the Annual Preventive Program for HIV for 2017 was budgeted for civil society-led and key population-focused services.   - The transition plan was drafted and adopted by the Country Coordinating Mechanism (CCM) in December 2016. Both documents propose the development of an effective social contracting mechanism to ensure that funds are disbursed to civil society implementers.   - Political commitment to developing a national system for financing NGO-delivered services remained strong. By late October 2017, the Ministry of Health had signed contracts with 13 NGOs to cover services for the last quarter of 2017. - In Serbia: - Serbia’s country coordinating mechanism has been non-operational since 2014 as per UNDP. - In September 2014, the Ministry of Health organized a consensus conference that affirmed the importance of continuing HIV services for key populations. Soon after, the transition process was blocked and all the efforts and gains were lost. - The National AIDS Strategy expired in 2015 and was not extended   *Rodriguez et al. (2017) – Multiple countries (Ukraine, Serbia, and Mexico)*   - As transition occurred, lack of political across all countries examined led to loss of the health gains made and worsening health outcomes in the post-transition period. Prevailing challenges include: the Ukrainian government’s reluctance to honor their funding commitment for HIV prevention; the Serbian government not funding prison-based services; and Mexico eventually eliminating family planning support for special populations.   *Wang et al (2017)* – *China*   - China is developing strategies and policies to address financial challenges such as piloting a model for sustainable financial and care packages for TB and multi-drug resistant (MDR) TB patients conducted in four prefectures. - Introducing additional policies to ensure the provision of an MDR-TB quality package of care and protection of patients from medical and associated non-medical costs including increased insurance reimbursement rates (up to 70%), social protection funds for the poorest patients and the continued use of the existing public health package for TB.   *Government of Nepal (2018) – Nepal*   - The government showed its commitment by increasing its funding contribution to maintain sustainability.   *STOPAIDS (n.d.)* *– Multiple countries (India, Estonia, Serbia, Thailand, Vietnam)*   - Communicating decisions related to funding and transition between funder and the country government is important. This is especially important when discussing challenges related to decision changes (e.g. Thailand). - Governments honoring the commitments they make prior to transition is vital for post-transition success. Actions to fulfill these commitments have included continued coordination and planning, continued funding, and supporting programs that reach marginalized groups (e.g. harm reduction programs) following donor departure.   *UNDP (n.d.) – Iran*   - Developing a transition plan with multi-stakeholder involvement leads to sustainable programs with the inclusion of domestic management bodies. - Integrating lessons learnt from the Global Fund program during transition into the national strategic plan for tuberculosis provided an investment case for national and provincial resources to be allocated to the national tuberculosis program.   *UNDP (n.d.) – Belarus*   - Identifying new principal recipients from the beginning is key for coordination, budget allocation and to prevent confusion post transition. For example, the budgets from the Belarus government and UNDP were not aligned during the transition of funding from UNDP to the government. - The Global Fund Country Coordinating Mechanism should be involved at all stages of transition to ensure coordination between the new and old principal recipients. - There is a need to ensure that all relevant entities (e.g. government ministries) are involved in the transition process. |
| Nutrition | USAID | *World Food Programme (2003) – Multiple countries (Cape Verde, Brazil, Botswana, El Salvador, Jamaica, Namibia, and Swaziland)*   - Although Botswana was economically prepared for transition, the government lacked critical skills to efficiently manage the nutrition program and manage oversight. This highlights the importance of developing institutional capacity prior to donor departure. - In Namibia, Brazil, and El Salvador, strong political commitment and creative approaches to manage transition in both funding and capacity building led to successful sustainable programs following donor exit. - In Cape Verde, the transition forced the government to start from scratch raising new funding and building the required human resources. |

**Table 2. Impact of transitions away from development assistance for health on recipient countries health finance systems.**

| **Health Issue** | **Donors** | **Findings** |
| --- | --- | --- |
| Family planning | USAID | *Coury and Lafebre (2001) – Ecuador*   - In preparation for transition, non-governmental organizations in Ecuador strategized to strengthen financial and administrative management systems by creating agreements with the private sector. - The government of Ecaudor faced challenges with financing contraceptive supplies in the post-transition period.   *USAID and the Ministry of Health of the Kingdom of Morocco (2003) – Morocco*   - The Ministry of Health of Morocco agreed to assume financial responsibility as USAID gradually withdrew financing for specific elements. - The transfer of contraceptive purchasing to the Ministry of Health was carried out as planned and even exceeded expectations. As of 2000, Ministry entirely covered the purchasing of contraceptives and financial provisions were in place till 2004, partially supported by a loan from the World Bank. - Public and private sector partnership and social marketing programs contributed to the sustainability of family planning and child health activities by diversifying sources funding.   *Sine et al. (2004) – Turkey*   - Obtaining annual budget allocations for contraceptives and targeting of free services to the poor were two central components of Turkey’s self-reliance strategy.   *Alkenbrack and Shepherd (2005) – Mexico*   - As USAID funding reduced, Mexico took two strategies to fill the funding gap: 1) looking for support from other international donors and 2) increased government funding - Unfortunately, due to lack of awareness at the state level, other priorities competed with family planning funding, and organizations did not allocate substantial funding to contraceptives. It also made financial allocations unpredictable from year to year resulting in difficulties with planning and budgeting. - On the other hand, non-governmental organizations achieved financial sustainability by generating internal funds, mobilizing funds from non-USAID donors, charging user fees and contraceptive sales, cross subsidization of programs, use of social marketing, creation of affiliated pharmacies and a revolving fund.   *Bertrand (2011) – Multiple countries (Paraguay, Honduras, Nicaragua, and Peru)*   - Paraguay demonstrated its ability and commitment to cover all contraceptive purchases for the public sector. - Honduras and Nicaragua were uncertain about their commitment and ability to have a fixed line item for contraceptives in the national budget. - Peru’s government was willing and able to provide financing for contraceptives. However, inconsistent government commitment in the past and the larger health sector reform process that the country was undergoing created challenges for the family planning program.   *Chaudry et al. (2012) –*  *Multiple countries (South Korea, Panama, Sri Lanka, Tunisia, Botswana, Thailand, Costa Rica, Swaziland, Colombia, Mexico, Brazil. Ecuador, Morocco, Turkey, Indonesia, Romania, Jamaica, Dominican Republic, South Africa, El Salvador, Paraguay, Nicaragua, Honduras, Peru, Albania, Ukraine, Egypt, Bangladesh, and Zimbabwe. Specific examples from Colombia, Ecuador, Egypt, Morocco, Dominican Republic, Paraguay, Peru, El Salvador, and Nicaragua.*   - When assistance was coming to an end, USAID actively collaborated with other donors and advocated for establishing a line item in the government budget for family planning commodities in many of the countries. Tapping local capital and partnering in new ways to unleash sustainable financing were keys to success. - Different countries used various strategies for financial sustainability. These included: establishing independent businesses (Colombia); creating an endowment fund that links performance to financing (Ecuador); having civil society organizations provide a significant portion of commodities, services etc. (Egypt, Ecuador, and Morocco); establishing insurance funds, especially focused on vulnerable populations (Dominican Republic); and establishing a community-based health insurance scheme and a logistics management information system (Paraguay). - There were specific concerns about the graduation strategies related to uncertainties in financing (especially regarding commodities) and national-level political commitment that could not be fully addressed within the context of the graduation, as was seen in Peru, El Salvador, and Nicaragua.   *Shen et al. (2015) – Multiple countries (Dominican Republic, Egypt, El Salvador, Honduras, Jamaica, Kazakhstan, Nicaragua, Paraguay, Peru, Ukraine and Uzbekistan)*   - Support for the inclusion of a budget line item for family planning programming before, during and after graduation at the national level ensured sustainability.   *Avila et al. (2016) – Nicaragua*   - The Ministry of Health in Nicaragua increased funding for contraceptive procurement starting in 2006, before the implementation of USAID’s Family Planning Graduation Strategy in 2007. Therefore, the overall implementation of the USAID Nicaragua graduation plan was a success. Public sector efforts targeting the poor, uneducated and inaccessible populations was successful resulting in better financial equity reducing out of pocket expenditure of the poorest population. |
| Immunization | Gavi, the Vaccine Alliance | *Levin et al. (2010) – Multiple countries (Armenia, Azerbaijan, Afghanistan, Burundi, Bhutan, Albania, Cambodia, Burkina Faso, Angola, Djibouti, Cameroon, Bangladesh, Ethiopia, Central African Republic, Bolivia, Gambia, Comoros, Chad, Georgia, Democratic Republic of Congo, China, Democratic Republic of Korea, Ghana, Congo, Lao People's Democratic Republic, Indonesia, Eritrea, Senegal, Kenya, Guinea, Sierra Leone, Lesotho, Haiti, Sudan, Mali, Honduras, Uganda, Mozambique, Republic of Kyrgyz, Yemen, Myanmar, Mauritania, Zambia, Nepal, Niger, Pakistan, Sri Lanka, Rwanda, Tajikistan, Sao Tome, Turkmenistan, Somalia, Ukraine, Tanzania, Vietnam, Togo, Zimbabwe, and Uzbekistan).*   - By mid-2008, 25 (54%) of all countries fully-funded their syringe and safety box purchase, 15% used a mixture of government and donor funding and 12% remained completely donor dependent.   *Gavi (2013) – Mongolia*   - The immunization fund set up by the government of Mongolia had multiple funding sources, including voluntary vaccination fees.   *Gavi (2014) – Bosnia and Herzegovina*   - Transition from Gavi support to domestic funding for vaccine procurement was smooth overall. However, stakeholders had to navigate substantial risks such as small markets, fragmented procurement systems, low competition, and high vaccine prices.   *Gavi (2015) – Albania*   - A separate budget line for vaccine procurement by Ministry of Health in Albania ensured financial sustainability for vaccines. The government midterm expenditure framework additionally ensures the provision of financial resources for vaccines and injection supplies. - However, there was no strategic approach and funding for cold chain maintenance.   *Kallenburg and Cornejo (2015) – Countries transitioning out of Gavi support after 2015*   - Limited budgets for immunization programs and competing domestic priorities affected post-transition funding.   *Saxenian et al. (2015) – Multiple countries (Bhutan, the Republic of Congo, Georgia, Mongolia, and Moldova)*   - In preparation for transition, countries benefited from creating detailed financial projections where none existed before.   *Bliss and Peck (2016) – Honduras*   - Honduras provided 20% more vaccine financing annually after passing the Gavi eligibility threshold.   *Kallenburg et al. (2016) – Multiple countries (Angola and the Republic of the Congo)*   - For countries in the accelerated transition process, required increase in domestic financing for vaccines can exceed the projected economic growth and health budget growth. It also requires reallocation of funds from other sectors or within the health budget.   *Kerr (2017) – Multiple countries (Sri Lanka and the Republic of the Congo)*   - Economic factors affecting the country during transition also influenced the financing and service delivery systems in the republic of the Congo. - Sri Lanka had to increase its co-financing of new vaccines by 197% between 2012-2018. This prioritization of financing by the government resulted in a smooth transition process.   *Almatov et al. (2017) – Uzbekistan*   - Government provided additional funds of USD 3 million to strengthen the material and technical base of district warehouses to store vaccines.   *Dadzie et al. (2017) – Ghana*   - Government of Ghana provided USD 16.3 million for immunization activities in 2017 yet delays in release of funds for vaccine remained a challenge.   *Results for Development (2017) – Multiple countries (Armenia, Indonesia, Sri Lanka, Vietnam, Georgia, Timor-Leste)*   - Many countries estimated budget needs based on the five-year strategic plans for immunizations (Armenia, Indonesia, Sri Lanka, Vietnam) with considerations of challenges such as increase in vaccine prices prior to procurement (Armenia and Georgia) or fluctuations in exchange rate (Georgia). - Limited fiscal space in the context of Gavi transition remained a challenge in countries.   *Results for Development (2017) – Multiple countries (Bhutan, Ghana, and Sri Lanka)*   - In Bhutan, Health Trust Fund invested in short-term deposits, fixed deposits and received annual contribution of USD 2.1 million from MOF since 2015. - In Ghana, the decreasing Ministry of Health budget made health facilities rely more on the payment of claims for services by National Health Insurance Scheme (NHIS) to cover the day to day operations and some immunization delivery costs in facilities. Therefore, curative services may be crowding out preventive services that are not included in the NHIS. On the other hand, the NHIS increased the diversity of funding base for Ghana’s immunization program, which could help ensure more stable funding in the future. - In Sri Lanka, The Advisory Committee on Communicable Diseases led rigorous decision-making processes that included steps to prepare Sri Lanka for a smooth financial transition.   *WHO (2017) – African Region*   - During the accelerated transition phase of Gavi, most countries face challenges in meeting the co-financing obligations (Angola, Congo, Ghana). - Some faced specific challenges such as economic slowdown alongside stock-outs in BCG, penta, measles and yellow fever vaccines due to delays in meeting co-financing obligations (Angola). Other challenges included securing increased investment for vaccines from the government amid economic crisis (Congo); crowding out of immunization financing due to wage bill and inadequate financing for immunization (Ghana); and finance management capacity (Nigeria)   *Cernuschi et al. (2018) – Multiple countries (Angola, Armenia, Azerbaijan, Bhutan, Bolivia, Congo Republic, Georgia, Ghana, Guyana, Honduras, Moldova, Mongolia, Papua New Guinea, Sri Lanka, and Uzbekistan)*   - 13 of 15 countries have a budget line-item for immunization and 62% finance a large portion of routine vaccines via government sources. However, nearly half of the countries sampled in this study, especially the countries in the African region, have defaulted at least once in Gavi co-financing requirements. 67% of sample countries report shortfalls in financing for immunization. In-country assessments revealed a lack of skills and processes to develop sound financing and resource mobilization strategies.   *Gavi n.d.b – Honduras*   - After receiving 50% financing for HPV vaccination by Gavi in the first year, the government of Honduras started complete funding in the second year. |
| HIV/AIDS and Tuberculosis | Global Fund to Fight AIDS Tuberculosis and Malaria;  Bill and Melinda Gates Foundation;  US Presidents Emergency Plan for AIDS Relief;  The World Bank | *Torpey et al. (2010) – Zambia*   - Graduation was focused on technical and programmatic sectors, whereas financial support continued after graduation.   *Walsh et al. (2012) – Zambia*   - Funding for community-based organizations (CBOs) in Mumbwa had decreased since the end of CRAIDS funding in 2008. Although national stakeholders indicated other funding sources as the ministry of community development, Mother and Child Health under guidance from National HIV/AIDS Council, and the ministry of education runs a public welfare assistance scheme, many CBOs were not aware of other funding opportunities. - Some CBOs voiced that the Zambia National AIDS Network (ZNAN), the civil society umbrella body funded by the Global Fund, made it difficult for communities to access funds.   *Katz et al. (2013) – South Africa*   - During transition, there was tension on how to continue the ART treatment and care at affordable prices. PEPFAR-funded sites also had to determine how to safely transfer patients to local clinics and follow-up care for patients without further donor funding.   *UNAIDS (2013) – Multiple countries (Swaziland, Kazakhstan, and Namibia)*   - The government of Swaziland committed to fund all HIV related medicines following transition. - Civil society of Namibia, affected the most due to transition, presented a position paper on financing sustainability for the AIDS response. - The government of Kazakhstan commissioned a new national AIDs program to gradually take over funding of HIV prevention programs by 2015. Global Fund was the principal supplier of anti-retroviral drugs but the government’s share grew from 7% in 2007 to 100% in 2011.   *Sgaier et al. (2013) – India*   - Indian government’s third national plan took into consideration cost absorption of Avahan program and ensured that program costs were aligned with government guidelines six months prior to transition. - The Government also increased its HIV/AIDS budget by 400%between the second and third phases of its national program.   *Katz et al. (2014) – Multiple countries (Benin, Guyana, Kenya, Lesotho, South Sudan, and Sierra Leone)*   - Kenya established a levy on airline tickets, increased government budget allocation for health to 15% and modestly raised National Hospital Insurance Fund to generate funds. - Across all countries, sustaining non-governmental organizations, as the primary providers of HIV prevention services to most-at-risk groups, was a challenge.   *Amaya et al. (2014) – Peru*   - HIV/AIDS programs were included strategically in a results-based budget to focus resources on key populations. The government investment in HIV/AIDS grew from 0.2% to 0.4% of the total budget from 2011 to 2012 but the poor execution of funding allocation and management of funds presented challenges.     *Cairney and Kapilashrami (2014) – Namibia*   - As per the government of Namibia, PEPFAR’s decision to cut 10% annual from the budget for human resources was problematic since the decision did not consider the input of the government and there was not enough time to develop a strategy to address the effects of the budget cuts.   *Eurasian Harm Reduction Network (2015) – Bulgaria*   - The Ministry of Health in Bulgaria planned to allocate USD 0.06 million annually to HIV prevention as opposed to Global Fund’s USD 3.2 million. Lack of funding for harm reduction remained a threat to sustainability.   *Eurasian Harm Reduction Network (2015) – Serbia*   - Lack of funding for non-governmental organizations left a gap in HIV prevention work as government did not step in or provide adequate funding.   *Bennet et al. (2015) – India*   - The government increased allocations towards programming and funding to non-governmental organizations (NGOs), but the budgets for NGOs were still insufficient for salaries, travel, or events.   *Bennett et al. (2015b) – India*   - Financial challenges faced in first round of transition were addressed in the second round with dedicated budgets and financial reserves. These actions were further formalized in the third round of transition.   *Rodriguez et al. (2015) – India*   - Due to budget decreases, staff salaries and travel allowances were reduced, which further led to job dissatisfaction. The reduction in budget also affected social community events and tangible incentives for key populations.   *Vogus and Graff (2015) – Countries graduated from PEPFAR by 2013 in Latin America, Caribbean and countries under transition from PEPFAR in 2013 (specific examples from South Africa)*   - Although PEPFAR funding was expected to reduce in South Africa, the pace of the reduction was unclear which affected funding of prevention programs and high impact interventions that were historically funded by donors.   *Burrows et al. (2016) – Multiple countries (Papua New Guinea, South Africa, and Thailand)*   - Funding for HIV key populations reduced during and after transition from Global Fund support across countries examined. - In Thailand, 100% of HIV prevention of drug users was funded through Global Fund. It is unclear if the Thai government will continue sustained funding for this population.   *Eurasian Harm Reduction Network (2016) – Montenegro*   - Despite expressing support for HIV/AIDS programming, the government did not have clear plans to fill the funding gaps after Global Fund’s departure.   *Eurasian Harm Reductio Network (2016) – Romania*   - Despite government’s financial commitment, domestic funding gap for harm reduction interventions existed such that only 7% of harm reduction spending for persons who inject drugs came from domestic sources in 2011. This resulted in increase in HIV incidence in key populations. - The ministry of public health lacked the will to develop funding procedures to contract services to non-governmental organizations. Additionally, no cost projections or need projections were conducted in Romania to inform the sustainable financing for HIV/AIDS and harm reduction interventions.   *Eurasian Harm Reduction Network (2016) – Macedonia*   - The transition plan did not include a clear financial commitment by the government of Macedonia.   *Eurasian Harm Reduction Network (2016) – Albania*   - No commitment from the government of Albania to fill funding gap, combined with a lack of transition planning, led to funding gaps following donor exit.   *Health Policy Project (2016) – Multiple countries (Bangladesh and China)*   - The reduction in PEPFAR funding concurrent with funding reductions from other donors affected the HIV/AIDS response, especially for key populations.   *Health Policy Project (2016) – Bangladesh*   - In the post-transition era, some donor activities, regardless of the prior successes they had achieved, were too costly to continue and many civil society organizations faced difficulties with sustaining funding.   *Health Policy Project (2016) – China*   - In 2014 the government financed almost all (99%) of its USD 987 million in HIV expenditures. The Chinese government developed and refined national- and provincial-level funding mechanisms called social service outsourcing (SSO), through which civil society organizations (CSOs) can apply for HIV program funding. - However, there was no immediate funding from SSO after the withdrawal of international donors in 2013. The national SSO mechanism only began accepting applications in July 2015, and funds were not available until in 2016. The funding from Chinese government focused on different services as supported by donors. There was short-term reduction in funding to CSOs and a focus on maintaining specific service quotas.   *International Council of Aids Service Organizations (ICASO) (2016) – Serbia*   - Serbia received more than USD 30 million since 2003, and the abrupt ending of funding in 2013 due to ineligibility resulted in 50 local HIV organizations losing 90% of their funding.   *Ozawa et al. (2016) – India*   - During post-transition, flexibility on budgets, allowing exceptions to operating norms, having on-time, adequate and uninterrupted flow of funds to the grassroots level were often not continued which deeply affected the normal operations of the Avahan HIV/AIDS prevention program.   *Rivalan (2016) – Serbia*   - In 2013, Serbia’s loss of funding from Global Fund resulted in the closure of risk reduction and prevention program for highly vulnerable groups. With only 3% allocated for prevention activities, the National HIV/AIDS budget did not include risk reduction programs for vulnerable groups.   *UNAIDS (2016) – Argentina*   - Funding to support community programming has increased each year since 2011. In 2011, a total of 971,000 Argentine pesos (USD 103,384) was granted to 15 organizations and in 2015 a total of 2.1 million Argentine pesos (USD 223,591) was allocated to 14 organizations working in several high-priority areas.   *Marten (2017) – Tanzania*   - Decreasing funding from PEPFAR affected service delivery by non-governmental organizations and confidence of patients who increasingly missed their appointments.   *Open Society Foundation (2017) – Multiple countries (Macedonia, Montenegro, and Serbia)*   - Macedonia sustained HIV services by approving a budget for the annual HIV program that included specific lines for each of the key populations in 2017. In 2018, the Ministry of Health allocated roughly USD 1.6 million to the National HIV Program to provide continuous anti-retroviral therapy and sustain HIV prevention programs among key affected populations. This allocation was a fourfold increase from the previous year’s budget commitment that maintain the similar levels of funding previously supported by the Global Fund. - Montenegro made progress on building domestic funding support for HIV programing. It passed legislation to allocate 100,000 euros for non-governmental organizations (NGOs) providing services for people living with HIV/AIDS and affected populations in 2016 and 2017. It also used the disbursements from lottery fund in 2016 to cover about a third of previous Global Fund contributions for prevention work with men who have sex with men (MSM), and a quarter of that for prison-based prevention programs. Programs for sex workers received lottery funds in 2017, but at only 13% of previous amounts. Due to increasing disease burden among MSM, Macedonia became eligible for limited Global Fund funding in 2017-2019 which had added safeguards to ensure that domestic ownership remains improving. - The Serbia government began funding ART, opioid substitution therapy and voluntary testing and counseling, which benefited the population even before Global Fund. In 2015, Serbia spent 8 million euros on ART and 1.2 million euros on opioid substitution therapy. Local and regional authorities provided an additional 60,700 euro for non-governmental projects in the HIV field. However, the transition still saw a steep decline in funding for NGOs which led to collapse in NGO-led services especially affecting key populations   *Government of Nepal (2018) – Nepal*   - In 2018-2019, the Government of Nepal planned to assume full financial responsibility for the elimination of vertical HIV transition (eVT) program, TB-HIV interventions, as well as prevention programs for migrants, those in prisons and other closed settings.   *UNAIDS Program Coordinating Board Meeting (2015) – Multiple countries (Kazakhstan, Namibian, India, Thailand)*   - While international HIV assistance to Kazakhstan declined by more than half from 2011 to 2015, domestic public sector expenditure rose by 73%. - The Namibian government increased its investment in the AIDS response from USD 111.1 million in 2012-2013 to USD 136.6 million in 2013-2014, covering 55% and 64% of total HIV related costs, respectively. By 2017, Namibia’s public sector was projected to cover 70% of all HIV related expenditure. - In 2015, India’s public sector covered 63% of HIV-related expenditure, with the Global Fund and the World Bank contributing 14% and 10%, respectively. Previous iteration of funding had international sources funding 80% of all HIV-related investments. - Thailand aimed to mobilize roughly USD 21 million per year to support civil society activities relating to the AIDS response. Existing and potential sources of public and private sector financing for civil society engagement were being reviewed and analyzed   *UNAIDS Program Coordinating Board Meeting (2016) – For countries receiving funding from Global Fund*   - Countries moving out of low-income and lower-middle income status to upper-middle income and high-income categories in Latin America, the Caribbean, Eastern Europe, Central Asia, and Asia Pacific reported a general trend in losing funding from international donors.   *TB Europe Coalition (2016) – Multiple countries (Azerbaijan, Croatia, Serbia, Kyrgyzstan, Romania, and Ukraine). Specific examples from Serbia and Croatia.*   - The Serbian government failed to fill the funding gap after the transition of the Global Fund. Their 2015 national budget did not include any funding for prevention in key populations and only 3% of HIV budget was allocated for prevention. - In Croatia, many aspects of the HIV programs were already domestically funded since 1998.   *TB Europe Coalition (2017) – Bulgaria*   - Although the Bulgarian government committed to funding new TB and HIV programs, the level of committed funds was insufficient for the full range of interventions previously funded by the Global Fund, especially those focusing on key populations.   *Wang et al. (2017) – China*   - For multi-drug resistant (MDR) TB, the concomitant completion of the Global Fund project and on-going health reforms posed significant challenges. In 2014, the Global Fund provided 26% of the total reported budget for MDR-TB, while domestic funding comprised 19%, leaving a 55% funding gap. - Case-based payment was planned as a measure to control hospital costs and ensure quality of delivery. Selected provinces have further legislated financial protective measures to avoid catastrophic expenditure and harmonize reimbursement rates across the different health system levels. - Earmarked funds are being replaced by innovative mixed-source public health funding pools, combining central and provincial level funds, health insurance schemes, and out-of-pocket patient expenditure.   *UNDP (n.d.) – Iran*   - Incorporating financial sustainability into planning and linking costs to the National Strategic Plan helped assess options and inform management decisions.   *STOPAIDS (n.d.) – Multiple countries (Thailand, Serbia, Estonia, and Vietnam)*   - Due to lack of prioritization by the government, funding for harm reduction and people who use drugs was negatively affected after transition in Thailand and Serbia. - Government bureaucracy affects the processes. In Estonia, this halted funding for service delivery during transition and required government leadership to make exceptions in funding allocations. - Loss of funding due to transition and lack of domestic funding leads to funding gaps in particular for health programs (e.g. HIV/AIDS funding gap in Vietnam) |
| Nutrition | World Food Program | *World Food Programme (2003) – Multiple countries (Botswana, El Salvador, Jamaica, Namibia, and Swaziland)*   - In Botswana, the staged transition process over five years provided the government time to take over the financial responsibility and establish a budget line resulting in a smooth transition. - In El Salvador, Jamaica and Namibia, the governments secured additional budget resources to contribute financially from the beginning and eventually take over all the financial responsibility. - School feeding programs that incorporated funding from parental or community contributions in either cash payment or in kind (through donated food or labor), tend to be the strongest programs post-transition (e.g., like those seen in Jamaica, Namibia, and Swaziland).   *Rogers and Macías (2004) – Multiple countries (Gambia, Swaziland, and Cape Verde)*   - The Food-Assisted Child Survival (FACS) Program run by Catholic Relief Services in Gambia planned to integrate with the maternal and child health programs provided by the Department of Health. However, at the time of evaluation, the integration was unsuccessful in finding other sources of funds. - Continuing school feeding programs through user fees can disproportionately affect poor households causing students to drop out (Swaziland). Additionally, in the absence of a proper mechanism, only some people would pay the fee resulting in lack of funds (Cape Verde). |
| Multiple health issues | USAID,  Global Fund | *CRAT Via Libre (2017) – Guyana*   - National budget did not include any support for civil society organizations (CSOs) to conduct prevention programs, particularly for key populations. Many CSOs struggled to meet overhead and administrative expenses as external funding decreased.   *Crye (2011) – Multiple countries (Zambia and EL Salvador)*   - Funding cuts alongside an increasing number of treatment centers placed strain on financial and human resources during donor transition in Zambia. - Dedicated funding supply via a government trust-fund enabled El Salvador school feeding program to cover basic program costs while World Food Programme focused on capacity building.   *Global Fund (2013) – Multiple countries (Jamaica, Russia, South Africa, Kenya, and Estonia)*   - Following transition from Global Fund, most countries examined took financial responsibility for provision of drugs and other pharmaceutical commodities. - Prevention services were not sustained in Jamaica and Russia, especially ones that were delivered through community services and targeting key populations.   *Global Fund OIG (2018) – Multiple countries (Cuba, Albania, and Kosovo)*   - Transitioning countries increased their financial commitment to TB, Malaria, and HIV/AIDS in line with the co-financing policy. Countries have honored 82% of their previous co-financing commitments. - However, funding gaps remain which requires governments to increase domestic resources.   *Lewis et al. (2015) – Brazil*   - Government took on financing ensuring continuity by specifically investing malaria grant funds to support malaria surveillance, prevention and treatment at the termination of the grant.   *Oberth (2016b) – Multiple countries (Belize, Botswana, Bulgaria, Costa Rica, Fiji, Iran, Jamaica, Mauritius, Romania, South Africa, Suriname, Thailand, and Ukraine)*   - It is unclear if the growth of government spending is sufficient to cover program gaps in transitioning countries.   *Open Society Foundation (2015) – Multiple countries (Serbia, Thailand, Jamaica, Macedonia, Mexico, and Ukraine)*   - In Macedonia, the Ministry of Health has initiated registering non-governmental organizations to make them eligible to receive funding for HIV prevention. A key challenge for organizations serving key populations is that the social contracting mechanism requires organizations to provide funds up front. - In Serbia, the 2015 national HIV budget allocated just 3% for HIV prevention with no funds for key population. - In Ukraine, the needs of military and internally displaced persons has forced the government to reduce funding for health.   *Teixeira et al. (2017) – Multiple countries (Nigeria, Vietnam, and Serbia)*   - Nigeria was expected to fully finance its immunization program by 2022. However, in 2016 only 29% of immunizations expenditures came from government sources. Around 80% in 2015 and 100% in 2016 of the co-financing for the Penta and Pneumococcal vaccines came from World Bank loans that heavily supported Nigeria’s co-financing obligations. - Vietnam faced a decrease in overall external funding and a change in the form of assistance. This affected service delivery for particularly diseases like TB. As per WHO, in 2016 only 9% of the TB response was funded domestically, 22% by external sources and 69% went underfunded. - Serbia did not have the capacity to continue the HIV scale up program funded by the Global Fund from 2006 to 2014.   *UNDP (2015-2016) – Multiple countries (El Salvador and Zambia)*   - Capacity development enables countries to manage grant money on their own as principal recipients and leads to successful transition.   *UNDP (2016-2017) – Zimbabwe*   - A joint UNDP / Global Fund-financed capacity development plan in Zimbabwe was implemented in 2016 that the Ministry of Finance Public Financial Management System was rolled out in the health sector. This system provides ‘real time’ budgeting, electronic payment and an accounting system at central, provincial and district level for the TB and malaria grants, where the primary recipient role has transitioned from UNDP to the Ministry of Health. |

**Table 3. Impact of transitions away from development assistance for health on the health workforce in recipient countries.**

| Health issues | Donors | Findings |
| --- | --- | --- |
| Family Planning | USAID | *Alkenbrack and Shepherd (2005) – Mexico*   - Technical capacity training was conducted at national, state, district, and non-governmental organization (NGO) levels as a part of the phase out process. At the district level, USAID trained medical coordinators and warehouse managers from 16 states—the targeted states as well as other states. - Technical assistance to the NGOs was provided to the central level and focused on training staff for data collection, forecasting and preparation of procurement budgets. - State-level decision-makers did not receive training for procurement, which left a gap. Lack of knowledge of the General Directorate of Reproductive Health (DGSR) in estimating the costs of contraceptives resulted in many states not procuring contraceptives or overspending. - Large cushion-stocks of supplies received by USAID (in the NGO sector) and UNFPA (in the public sector) led to overstocks and delayed the need for procurement leaving a technical capacity gap as full warehouses prevented trial procurement during phaseout. - High turnover of USAID key staff weakened implementation since there was no one to liaise with key government leaders during phaseout. Staff reductions have taken place at each of the public sector organizations, mainly because organizations had less to manage after phaseout and did not require the same level of staffing.   *Chaudry et al. (2012) – Multiple countries (South Korea, Panama, Sri Lanka, Tunisia, Botswana, Thailand, Costa Rica, Swaziland, Colombia, Mexico, Brazil. Ecuador, Morocco, Turkey, Indonesia, Romania, Jamaica, Dominican Republic, South Africa, El Salvador, Paraguay, Nicaragua, Honduras, Peru, Albania, Ukraine, Egypt, Bangladesh, and Zimbabwe)*   - Graduation strategies of USAID emphasized strengthening the human and institutional capacity to implement and manage activities including transferring knowledge and skills.   - For example, in Nicaragua, USAID supported establishing NicaSalud, a federation of 28 non-governmental organizations (NGOs). In El Salvador, USAID supported the NGOs ADS and PROFAMILIA; in Honduras, they supported ASHONPLAFA NGO with financial and technical assistance; and in Ecuador, they supported the NGO Asociacion Pro-bienestar de la Familia Ecuatoriana, as well as a social marketing program, Centro Medico de Orientacion y Planificacion Familiar, to provide family planning services.   - In Indonesia, USAID strengthened non-governmental and professional organizations such as the Indonesian Midwives Association, the Indonesian Medical Association, the Indonesian Association for Permanent Contraception, Nahdatul Ulama, Muhammadiyah, and the Coalition for a Healthy Indonesia who currently provide leadership and service delivery related to family planning. |
| HIV/AIDS | Bill and Melinda Gates Foundation (BMGF) | *Sgaier et al. (2013) – India*   - The Bill and Melinda Gates Foundation during transition assisted the government to develop skills in data-driven management and field supervision, community mobilization, guidelines development, effective management and costing. - Challenges related to workforce included elongated time period it took for capacity building and transferring knowledge (in this instance 8 years), staff turnover in the government especially at the state level, staff attrition, low morale in agencies and concerns from the community about the change in management. - Staff turnover challenges underscored the need for formal agreements with the government and the importance of having in country staff as it was time consuming to orient incoming national and state level leaders to Avahan programs.   *Bennett et al. (2015) – India*   - In preparation for transition and while transitioning, Avahan program built capacity of local partners (the government, communities, and NGO) strengthening the local system. - Aligning interventions with government norms and guidelines led to cutting staff and reducing salaries. Some State Lead Partners were able to negotiate and maintain the salary levels. Aligning with the government norms also meant that there are high literacy level requirements which affected staff recruitment especially from key populations. - Additionally, as norms were aligned with the government, budgets for trainings, meetings, travel allowances, outreach and office expenditure were also cut affecting incentives and operations. - In most targeted interventions performance monitoring of peer educators improved with higher and concrete performance standards. Those who did not meet standards were terminated. Additionally, in targeted interventions the trainings for peer educators were considered to have improved.   *Bennett et al. (2015b) – India*   - As Avahan program transition progressed the number of trainings for staff increased with approximately 70% of transitioning targeted interventions having two or more staff members trained in different transition modules in 2012. In 2011 transition rounds, only 55% targeted interventions had two or more staff members trained in different transition modules and none were trained in the first round of transitions in 2009. - As the Avahan program transitioned, lower salaries were paid to targeted interventions staff due to different budgetary norms of the government.   *Bennett et al. (2015c) – India*   - Before and during transition there was tension between fidelity to the government norms and efforts by the donor (BMGF), non-governmental organizations (NGOs), and State Lead Partners to negotiate greater flexibility within the government norms. These tensions affected the work force as well. For example, the State Lead Partner negotiated higher salaries for every experienced NGO staff and in another NGOs negotiated flexibility in prescribed rations of peer educators to key populations in some states.   *Rodriguez et al. (2015) – India*   - Peer educators and key populations were informed about transition well in advance of the handover in all three rounds of the Avahan transition especially in Tamil Nadu. Outside of Tamil Nadu there were communication challenges leading to front line staff not being aware of the transition. - Peer educators faced challenges due to restricting their travel allowance as the program transitioned to government which the peer educators viewed as loss of income. In Tamil Nadu this frustration was less due to negotiated agreement of peer educators and key population ratio by the state lead partners: 1:60 for urban areas and 1:35 for rural areas. Tamil Nadu also employed a ‘group approach’ where a group of peer educators were responsible for key population monitoring allowing flexibility and reducing allowance related issues. - In Maharashtra and Tamil Nadu, front line workers remained committed to targeted interventions despite the limited salaries and allowances. |
| HIV/AIDS | Global Fund | *Amaya et al. (2014) - Peru*   - The poor technical capacity of the sexually transmitted infection (STI) and HIV strategy office was compounded by a significant number of personnel within the strategy team hired with the Global Fund money. There were officially 3 people hired by the Ministry of Health and 10 more people hired with Global Fund support.   *Euroasian Harm Reduction Network (2016) – Montenegro*   - Following the departure of the Global Fund lack of funds has resulted in reduction of the workforce. This reduction has affected outreach efforts with reduced number of outreach workers and has also led to closure of drop-in centers.   *Government of Nepal (2018) – Nepal*   - As the Global Fund transitions out of Nepal the government is increasingly taking over support for human resources, commodities and services that have been supported by the Global Fund. |
| HIV/AIDS, Tuberculosis, and Malaria | Global Fund, PEPFAR, UNDP | *Cairney and Kapilashrami (2014) – Namibia*   - Human Resource transition when scaling down from the Global Fund and PEPFAR has created challenges administratively and financially. These challenges were compounded by prior directives to reduce salaries of positions by both PEPFAR and the Global Fund after many years of funding. - The Ministry of Health set up a Human Resources for Health (HRH) task force in 2011 due to having no long-term solutions for scaling down of human resource funded by the Global Fund and PEPFAR. The task force planned to oversee donor funded human resources being transitioned to government by fiscal year 2014/2015. By March 2012, only 41 medical doctors were transitioned, although the government declared to transition every critical position. However, as per officials of the ministry aware of the system, the timing of transition was not certain due to the need for government cabinet approval.   *AMFAR (2015) – Multiple countries ( Jamaica, Nigeria, South Africa, Ukraine, Vietnam, and Zambia)*   - Some PEPFAR funded non-public providers went out of business after withdrawal of PEPFAR. There are concerns withdrawal of PEPFAR could impede further progress in bringing essential services to scale.   *Presidential Commission on HIV and AIDS Republic of Guyana (2015) – Guyana*   - Staff attrition when transitioning from the donor to government was a major challenge in 2014. Staff attrition especially affected HIV treatment program management and national laboratory testing services such as viral load and DNA PCR testing. In 2014, HIV treatment program operated without program coordinator and significant attrition of overall staff. |
| HIV/AIDS | PEPFAR | *Torpey et al. (2010) – Zambia*   - Challenges related to transition included poor staff attitudes towards quality assurance and quality improvement (QA/QI) prior to training, high turrnover and perceived extra workload. Facility healthcare workers often perceived the graduation strategy as an extra duty thus expecting additional financial renumeration. However, several facility level healthcare workers, as well as Ministry of Health officials at the national and provincial level showed interest in quality improvement and graduation sustainability strategy.   *Kavanaugh (2014) – South Africa*   - During transition retaining trained staff in AIDS response has faced challenges due to slow public sector systems, hiring freezes. Many physicians and nurses have moved out of direct services into ‘mentoring’ and others have left the sector. - Both the South African government and PEPFAR in 2012, lacked a coordinated strategy to retain thousands of the community health workers who were critical to PEPFAR’s success.   *Vogus and Graff (2015) – Countries graduated from PEPFAR by 2013 in Latin America, Caribbean and countries under transition from PEPFAR in 2013 (specific examples from Bostwana and Malawi)*   - Evidence suggests that building technical capacity is important during transition. For example, evidence from Mexico revealed that donors should have attempted to institutionalize technical capacity for program areas like commodity procurement. Similar observations were made in Botswana and Malawi. - Lack of building capacity of the in-country stakeholders to finance procurements and oversee logistics led to challenges when donors left. Finance procurements and logistics were handled by USAID and countries faced challenges during transition In addition to existing challenges related to hiring and training of health professionals. Foe examples, Bostwana faced these challenges when PEPFAR funding was decreased.   *Biradavolu et al. (2017) – Multiple countries (Mozambique, South Africa, and Namibia)*   - In South Africa and Namibia, the government faced management challenges in absorbing the healthcare workers trained under PEPFAR funder project during transition. - In Mozambique, the government faced challenges during transition due to PEPFAR project undermining the skills of volunteers in community-based home care while improving human resource capacity with a focus on efficient, technologically driven clinically based scale up. This undermining devalued both the non-technical skills of CHBC and the people who were seen to embody and represent the community such as older, poorly educated women.   *Marten (2017) – Tanzania*   - Workforce challenges as identified by key informants included a) weak healthcare system with less human resources, b) brain drain of healthcare workers, and c) extensive trainings that affect availability of the healthcare workers. - Lack of human resources:   - Supporting the claims of low human resources data points out that in 2015, Tanzania had 0.467 healthcare workers (physicians, nurses, and midwives) for every 1000 people, significantly lower than the WHO’s recommended target of 2.28 healthcare workers per 1000 people.   - In the public sector, there was a generalized 65% total shortage of health workers in 2006, with the largest deficiencies experienced at training institutions (74% shortage of staff) and village dispensaries (69% shortage). While in private sector, there was an estimated 85.9% shortage of health staff. - Brain drain of healthcare workers:   - Internal brain drain occurs due to healthcare workers in primary healthcare (PHC) being recruited to work in ‘enclaved’ HIV clinics sometimes withing the same health institution reducing workforce at PHC and due to recruitment of healthcare workers to private and NGO sectors reducing the practitioners in clinics and hospitals. Brain drain to the private and NGO sector also lead to lack of trainers to pass down technical skills to new recruits. - Long-term seminars and trainings impeding availability:   - There was an extensive use of in-service trainings, which was taking clinicians out of clinics. In a survey in southern Tanzania, 46% of available staff was out of the clinic for seminars and long-term trainings on the day of the survey, stretched thin an already overburdened healthcare workforce. |
| HIV/AIDS | Multiple donors (Global Fund to fight AIDS Tuberculosis and Malaria, PEPFAR, other bilateral and multilateral donors) | *UNAIDS (2013) – Multiple countries (Cambodia, Myanmar, South Africa, Swaziland, Kenya, Malawi, Namibia, and Kazakhstan)*   - In Namibia loss of funders resulted in loss of technical assistance and networks in addition to financial losses.   *UNAIDS Program Coordinating Board (2016) – Global (Includes all major donors and their recipient countries)*   - Challenges related to transition include reduction in the scope of community programs which will lead to organizations relying more on volunteers’ time and resources. This will lead to a slow death in community response. A representative from Asia and the Pacific reported that budget cuts can also lead to reduction in service delivery and fewer coverage across different provinces or districts. |
| HIV/AIDS, TB, malaria, and nutrition | Global Fund to Fight AIDS Tuberculosis and Malaria (GFATM); Presidents Emergency Program for AIDS Relief (PEPFAR); United Nations Development Programme (UNDP); World Food Programme (WFP); | *Crye (2011) – Multiple countries (South Africa, Zambia, and El Salvador)*   - During transition from AIDSRelief to the selected local partner Churches Health Association of Zambia (CHAZ), the phased transition plan allowed CHAZ to hire local staff who worked for AIDSRelief in key areas such as supply chain management, medical expertise in antiretroviral therapy, and monitoring and evaluation. CHAZ staff was trained by AIDSRelief. - World Food Program (WFP) was able to provide capacity building for the work force at every level using their resources during transition. This was due to the coordinated effort with the government that saw the government having dedicated funds for program costs freeing up WFP resources for training.   *CRAT Via Libre (2017) – Guyana*   - As funders transition, non-governmental organizations (NGOs) and civil society organizations (CSOs) face challenges managing administrative expenses affecting salaries and programs. Additionally, CSOs and NGOs, even at the time of transition lack expertise in resource mobilization, proposal drafting and leadership training to face challenges related to transition. NGOs and CSOs need technical assistance to strengthen financial management systems, program monitoring and evaluation to sustain skills and effectiveness.   *UNDP (2016-2017) – Multiple countries (Angola, Afghanistan, Belize, Bolivia, Chad, Cuba, Dijibouti, Guinea-Bissau, Iran, Krygyztan, Mali, Panama, Sao Tome and Principe, South Sudan, Sudan, Tajikistan, Turkmekistan, Zambia, and Zimbabwe)*   - Capacity development of Provincial Medical Offices across Zambia involved the development of supporting software, strengthening financial management, and structured work-based mentoring by a joint team from UNDP and the ministry of health, to manage change and enhance skills.   *UNDP (n.d.) – Iran*   - In Iran capacity building helped make the transition of human resources smooth for example the TB program invested in recruiting qualified staff to deliver the main Service Delivery Areas. Additionally, further investments were made in the knowledge and skills of both the program staff and their government counterparts which in turn contributed to the success of the program. - The transition process included producing organograms, achievements of the program, terms of references and budgets to secure human resources, skills and knowledge. Additionally, to ensure transition of institutional memory and skills some staff working in the Global Fund program transferred to the government. |
| Immunization | Gavi, the Vaccine Alliance | *Gavi (2014) – Bosnia and Herzegovina*   - After the exit of Gavi, effectiveness of immunization operations has been affected due to limited human resources at ministry of civil affairs, health department. - Challenges to immunization sustainability is compounded by health system related challenges such as shortage of medical personnel, low pay, high turnover of health workers and the absence of an effective continuous professional development system.   *Learning Network for Countries in Transition (2017) - Ghana*   - Inequitable human resource distribution, weak supervision and monitoring at the national and sub-district level were considered to be challenges for Ghana when transitioning from Gavi   *Learning Network for Countries in Transition (2017) – Multiple countries (Armenia, Congo, Georgia, Ghana, Indonesia, Lao PDR, Moldova, Sri Lanka, Sudan, Timor-Leste, Uzbekistan, and Vietnam)*   - Across the countries Gavi transition faced challenges related to workforce such as limited availability of staff at the community level, low motivation and low accountability among healthcare workers.   *WHO (2017) – African Region (specific example from Angola)*   - Angola faced human resource related challenges during the accelerated transition phase of Gavi.   *Cernuschi et al. (2018) – Multiple countries (Angola, Armenia, Azerbaijan, Bhutan, Bolivia, Congo Republic, Georgia, Ghana, Guyana, Honduras, Moldova, Mongolia, Papua New Guinea, Sri Lanka, and Uzbekistan)*   - Two thirds of the countries transitioning from Gavi assessed in this study lack human resources to adequately provide quality health services, including immunization. Additionally, vaccine management is problematic in 60% of the countries assessed by this study. |
| Malaria | Global Fund to Fight AIDS Tuberculosis and Malaria (GFATM) | *Lewis et al. (2015) – Brazil*   - The government invested in grant funding to hire epidemiologists for malaria surveillance and prevention and provided technical assistance. At the termination of the grant the federal government financed the municipal epidemiologists ensuring continuity. |

**Table 4. Impact of transitions away from development assistance for health on medical products and technology in recipient countries.**

| **Health Issue** | **Donors** | **Findings** |
| --- | --- | --- |
| Family planning | USAID | *Coury and Lafebre (2001) – Ecuador*   - Non-governmental organizations set up new procurement systems for contraceptives when USAID-donations stopped. The USAID Mission made arrangements to provide technical assistance over the next year from the centrally managed Contraceptives Logistics Management project to two organizations.   *Alkenbrack and Shepherd (2005) – Mexico*   - The phase-out of contraceptives compelled the government and non-governmental organizations (NGOs) to discuss procurement processes. By 1995, the Health Secretariat began procuring contraceptives centrally on behalf of all participating states. UNFPA continued to provide donations during phase-out which included technical assistance for logistics but not for procurement. - NGOs were able to maintain large cushion-stocks following phaseout from USAID reserves till 2002. - Domestic contraceptives were much more expensive than imported contraceptives which had implications for the kinds of contraceptives that were being purchased and used by the population. - To streamline processes, the federal government took over procurement after phase-out. The consolidated procurement process does not include the state of Mexico, which has the highest number of potential users.   *Avila et al. (2016) – Nicaragua*   - There was an increase funding by the Ministry of Health and a decrease in use of USAID donations in contraceptive procurement. In 2010, the Government of Nicaragua provided 34.7% of all contraceptive funds; this value increased from 0.6% in 2006 to 74% in 2011, except in 2009, when UNFPA increased their donation, which resulted in the Ministry of Health reducing its budget. - Stock-outs decreased from 36% in 2007 to 0 8% in 2012 during transition process.   *Bertrand (2011) – Multiple countries (Paraguay, Honduras, Nicaragua, and Peru)*   - All 3 countries (Honduras, Nicaragua, Paraguay) developed considerable capacity in the different aspects of contraceptive security. - The Government of Honduras (GOH) procured its own contraceptives (condoms and injectables in 2002, USD 200,000 for IUDs and oral contraceptives in 2003) and committed to annually increasing its share of the purchase of contraception. But did not meet 100% of the cost until 2010.   *Chaudry et al. (2012) –Multiple countries (South Korea, Panama, Sri Lanka, Tunisia, Botswana, Thailand, Costa Rica, Swaziland, Colombia, Mexico, Brazil. Ecuador, Morocco, Turkey, Indonesia, Romania, Jamaica, Dominican Republic, South Africa, El Salvador, Paraguay, Nicaragua, Honduras, Peru, Albania, Ukraine, Egypt, Bangladesh, and Zimbabwe) Specific examples from Egypt, Honduras, Peru.*   - Including procurement as part of transition planning and USAID support to support country ownership led to positive results. For example, Peru formed a new entity for procurement services within the government; in Honduras the government gradually took over the procurement process, joint strategic plan led to government of Egypt taking complete responsibility of procurement.   *Health Policy Project (2013) – Paraguay*   - The contraceptive security committee reduced the incidence of stockouts, increased demand for and access to contraceptives, and increased use of sexual and reproductive health services. |
| Immunization | Gavi, the Vaccine Alliance | *Levin et al. (2010) – Multiple countries (Armenia, Azerbaijan, Afghanistan, Burundi, Bhutan, Albania, Cambodia, Burkina Faso, Angola, Djibouti, Cameroon, Bangladesh, Ethiopia, Central African Republic, Bolivia, Gambia, Comoros, Chad, Georgia, Democratic Republic of Congo, China, Democratic Republic of Korea, Ghana, Congo, Lao People's Democratic Republic, Indonesia, Eritrea, Senegal, Kenya, Guinea, Sierra Leone, Lesotho, Haiti, Sudan, Mali, Honduras, Uganda, Mozambique, Republic of Kyrgyz, Yemen, Myanmar, Mauritania, Zambia, Nepal, Niger, Pakistan, Sri Lanka, Rwanda, Tajikistan, Sao Tome, Turkmenistan, Somalia, Ukraine, Tanzania, Vietnam, Togo, Zimbabwe, and Uzbekistan)*   - Ukraine discontinued its use of Auto-Disable (AD) syringes and safety boxes and opted instead to ramp up its local production of standard disposable syringes. - Uzbekistan discontinued the use of AD syringes and safety boxes in three-quarters of its health facilities due to its decentralized procurement system. - 96% of countries receiving the support continued to procure and utilize AD syringes and safety boxes in the years after GAVI support came to an end.   *Gavi (2014) – Bosnia and Herzegovina*   - Intermittent vaccine and medical supply shortages were observed in the post GAVI period due to long and complicated procurement processes. However, access to new vaccines and coverage of target groups were not affected so immunization targets were met. - The Government needed to increase public funding 1) for the maintenance of ageing cold-chain equipment to ensure proper functioning of immunization programs, 2) to promote better waste management, and 3) to promote injection safety practices   *Gavi (2015) – Multiple countries (Albania and Bosnia and Herzegovina*)   - Post-transition, the Hepatitis B and pentavalent vaccines and Auto-Disable (AD) syringes are fully financed by the Government, and safeguarded in the Ministry of Health budget. - Albania did not experience a vaccine stock-out after Gavi support ended, unlike Bosnia and Herzegovina, where intermittent vaccine stock outs and shortages of medical supplies were observed. - Albania continued to introduce new vaccines – MMR, PCV and IPV in 2005, 2011 and 2014 – without donor support. Rota vaccine introduction was delayed until 2017 due to the lack of sufficient evidence on its cost-effectiveness and insufficient funds – prices were four to five times higher than Gavi prices. Introduction of new vaccines in the post-Gavi period did not happen in Bosnia and Herzegovina.   *Saxenian et al. (2015) – Multiple Countries (Bhutan, the Republic of Congo, Georgia, Moldova, and Mongolia)*   - All five countries studied in 2012 procured their GAVI-supported vaccines through the UNICEF Supply Division. Bhutan, the Republic of Congo, Georgia and Mongolia also use the Supply Division for their routine vaccines. - Georgia, Moldova and Mongolia carried-out some direct procurement of non-GAVI vaccines. - Moldova directly purchased routine vaccines at significantly higher prices through UNICEF and also experienced greater year-to-year price fluctuations. - In Mongolia, the government was unaware that some of the vaccines that it was procuring directly were offered at lower prices through UNICEF. Switching to UNICEF procurement could thus result in access to higher quality vaccines and to financial savings.   *Kallenburg and Cornejo (2015) – Countries transitioning out of Gavi support after 2015*   - The critical areas in a successful transition such as cold chain and supply chains for vaccines were sometimes neglected by governments, leaders, and partners.   *Kallenburg et al. (2016) – Multiple Countries (Angola and the Republic of Congo)*   - Angola and the Republic of the Congo have struggled to meet their co-financing requirements, which led to undersupply and stock-outs of vaccines because they introduced more vaccines, and have larger birth cohorts as well as weaknesses in their immunization program.   *Bliss and Peck (2016) – Multiple countries (Honduras and Nicaragua)*   - Honduras and Nicaragua both could not fully maintain and sustain the quality of their cold chain systems because of inadequate refrigerated storage for the newer vaccines at the regional level facilities.   *Dadzie et al. (2017) – Ghana*   - With Gavi transitioning from Ghana, the current cold chain storage capacity at national, districts and health facilities is inadequate.   *R4D (2017) – Multiple countries (Armenia, Indonesia, Sri Lanka, Vietnam, Georgia, and Timor-Leste)*   - Timor-Leste created a central medical goods procurement institution called Servico Autonomo Medicamentos e Equipamento de Saude (SAMES) with its own subaccount that can receive transferred funds from the ministry of finance more quickly to use in vaccine procurement. - Sri Lanka used the lower-priced live JE vaccine to introduce an MMR vaccine and procurement modalities such as introducing two presentations instead of one to reduce wastage, which provides other countries with extra efficient procurement choice. - Many countries expressed challenges related to procurement in the context of Gavi transition.   *WHO (2017) – African Region (specific example from Nigeria)*   - Nigeria is in the Gavi accelerated transition phase and is facing cold chain capacity challenges.   *Cernuschi et al. (2018) – Multiple countries (Angola, Armenia, Azerbaijan, Bhutan, Bolivia, Congo Republic, Georgia, Ghana, Guyana, Honduras, Moldova, Mongolia, Papua New Guinea, Sri Lanka, and Uzbekistan)*   - Countries have experienced more than one stock-out per year between 2010 and 2015 on average, with the most concerning results noted in the European region. - 73% of countries under study have identified at least one red flag in their current procurement practices.   *Gavi (n.d.) – Multiple countries (Sri Lanka and Timor-Leste)*   - Sri Lanka has successfully added the human papillomavirus (HPV) vaccine to its national immunization schedule, with Gavi support since graduating at the start of 2016. |
| HIV/AIDS and Tuberculosis | Global Fund to fight AIDS Tuberculosis and Malaria (Global Fund)  Bill and Melinda Gates Foundation (BMGF)  Presidents Emergency Plan for AIDS Relief (PEPFAR)  United Nations Development Programme (UNDP) | *Katz et al. (2013) – South Africa*   - Medication shortage and stock-outs were common in government-run clinics after transition.   *UNAIDS (2013) – Swaziland*   - Swaziland committed to increasing access to antiretroviral treatment by reducing drug costs, despite the loss of donor programs. Though the Swazi Government purchased 81% of its adult ARV drugs at international competitive prices in 2009, some contracted prices exceeded 130% of the prices listed in the Clinton Health Access Initiative (CHAI) ceiling price list, which functions as a benchmark for competitive pricing. Hence, key interventions including price benchmarking, evaluating supplier performance were used to push prices below the benchmark. In 2010, it was able to secure prices below 93% of its ARVs, and no contract price was more than 103% of the CHAI ceiling price in 2010. These price decreases reduced Swaziland’s ARV drug costs by 27%, compared to 2009 tender prices. This reduction amounted to cost savings of USD 4.91 million from January 2010 to March 2011.   *Kavanagh (2014) – South Africa*   - Clinics in many regions experienced stock-outs of ARV and tuberculosis (TB) drugs at the time of transition.   *Bennet et al. (2015) – India*   - Close to 70% of targeted intervention programs always had buffer stocks of condoms, medicines and funds to address potential gaps in supplies, such as in Karnataka and Maharashtra. - Commodity stock outs within a few months of the post-transition phase still existed for 30% of TI programs.   *Bennet et al. (2015b) – India*   - In India, there was success for targeted interventions to transition procurement channels for condoms and medicines for sexually transmitted infections to government suppliers. In some cases, these interventions built up buffer stocks, thus delaying the shift to procurement through government systems.   *Open Society Foundation (2015) – Multiple countries (Serbia, Thailand, Jamaica, Macedonia, Mexico, and Ukraine)*   - In Thailand, stockouts in needles and syringes continued for six months due to transition from one sub-recipient to another. Services via non-governmental organizations (NGOs) were suspended in 5 out of 19 provinces and a pharmacy-based voucher scheme was terminated. There were no plans of continuing coverage for hundreds of drug users. - Funding cuts increased the burden on service providers due to which all advocacy and support systems, including hepatitis C treatment, protection from arrest, collaboration with law enforcement, and operational research were eliminated. Basic funds for computers, logbooks and other training needs were also not available. Global Fund’s grant to NGOs for HIV prevention services was slashed by 50% — from USD 3 million per year to USD 1.5 million per year.   *Rodriguez et al. (2015) – India*   - Key populations complained that the transition led to more difficulty in accessing health services. - Considerable criticism regarding government commodities across targeted interventions, such as worse government condoms. Meanwhile, anti-itch ointment, pregnancy tests and painkillers became unavailable.   *Vogus and Graff (2015) – Countries graduated from PEPFAR by 2013 in Latin America, Caribbean and countries under transition from PEPFAR in 2013*   - PEPFAR’s Track 1.0 ART program helped Ministries of Health in 13 countries strengthen supply chain management and procurement of anti-retroviral therapy (ART). By 2011, the program had been successfully transitioned to country ownership and was providing ART for more than 925,000 patients.   *UNDP (2016) – Zambia*   - UNDP supported strengthening supply chain management for medical stores limited in Zambia with deployment of a new warehouse management system and piloting an inventory system.   *TB Europe Coalition (2016) – Multiple countries (Azerbaijan, Croatia, Serbia, Kyrgyzstan, Romania, and Ukraine)*   - The Global Fund was the main provider of TB drugs until 2011 in Azerbaijan. After that, the government took over the provision of first-line drugs and in 2015, 100% of second-line drugs were procured by the government budget for 500 DR-TB cases. Global Fund is now transitioning and would have left entirely by the end of 2017.   *European Harm Reduction Network (2016) – Montenegro*   - Some methadone maintenance therapy (MMT) centers (e.g. in Kotor and Bar) experienced a shortage of methadone, which resulted in 17 users at the end of 2014 in Bijelo Polje and 12 users in Podgorica continuing on MMT while serving a prison sentence.   *European Harm Reduction Network (2016) – Bosnia and Herzegovina*   - Post-transition, Naloxone/Buprenorphine therapy is funded by Health insurance funds in Sarajevo, Tuzla, and Zenica cantons up to 65% of the total costs needed for such therapy. - Naloxone/Buprenorphine is not on the Essential Medicines List in Republic of Sprska (RS) thus is not funded. Arrangements were made for Naloxone/Buprenorphine to be included in the Essential Medicine List in the RS and supported by health insurance funds in 2017. - Pharmaceuticals and health products procured within the Global Fund programs that were not likely to be used before 30 September 2016 were used for the period September 2016 – March 2017.   *European Harm Reduction Network (2016) – Romania*   - Decentralization of procurement and stock-outs – unclear if this was due to transition or compounded by transition. Non-governmental organizations and technical experts warned against a decentralized procurement system due to its lack of efficiency.   *European Harm Reduction Network (2016) – Macedonia*   - Opioid Substitution Treatment and antiretroviral therapy have already fully transitioned to government funding and national procurement procedures. - Currently, the National HIV Program includes procurement of condoms under the domestic budget but still hasn’t taken over other commodities such as needles and syringes.   *European Harm Reduction Network (2016) – Albania*   - Donor procurement systems, such as those for implementation of the Global Fund Round 5 grant, have not been integrated into national systems, resulting in a lack of assurance of reasonable price controls when purchasing equipment or commodities.   *Health Policy Project (2016) – Botswana*   - The Merck and Gates foundations invested heavily in Botswana and donated large volumes of antiretrovirals (Geertz et. Al., 2014). After it left, service delivery suffered significantly as the Government tried to fill the gaps.   *Health Policy Project (2016) – Bangladesh*   - The government was not well prepared to take on responsibility for procuring and providing ARVs through government hospitals in collaboration with civil society organizations, thus there were delays in medication procurement and delivery.   *Marten (2017) – Tanzania*   - Better equipment and infrastructure were needed in the post-transition period.   *UNDP (n.d.b) – Belarus*   - Transition of Procurement and Supply Chain Management functions are frequently the most challenging areas during transition and a staggered process is recommended to ensure there are no gaps in the supply of vital equipment and drugs. |

**Table 5. Impact of transitions away from development assistance for health on information and research systems in recipient countries.**

| Health issues | Donors | Findings |
| --- | --- | --- |
| Family Planning | USAID | *Chaudry et al. (2012) – Multiple countries (South Korea, Panama, Sri Lanka, Tunisia, Botswana, Thailand, Costa Rica, Swaziland, Colombia, Mexico, Brazil. Ecuador, Morocco, Turkey, Indonesia, Romania, Jamaica, Dominican Republic, South Africa, El Salvador, Paraguay, Nicaragua, Honduras, Peru, Albania, Ukraine, Egypt, Bangladesh, and Zimbabwe)*   - USAID’s graduation strategy included strengthening information technology and capacity to conduct program monitoring and evaluation, which also reflected broader health system reforms that supported program sustainability (e.g. Honduras, Indonesia, Paraguay, and Peru) - Additionally, USAID provided specific support as part of graduation strategy for institutionalizing Demographic and Health Surveys (DHS) or Reproductive Health Surveys (RHS) on a periodic basis, including ensuring local capacity and financing for continuation of these activities after graduation (e.g. Dominican Republic, Ecuador, El Salvador, Honduras, and Nicaragua).   *Avila et al. (2016) – Nicaragua*   - In the post-transition period, Nicaragua’s health information system continued to function well, but had to depend on a limited number of workers. |
| HIV/AIDS | Bill and Melinda Gates Foundation (BMGF) | *Sgaier et al. (2013) – India*   - Monitoring was a cross-cutting activity that were important to all sectors of transition to ensure evidence-based decisions making. - As continuous monitoring is key for smooth transition, regular reviews of program indicators and joint visit by representatives of the donor and government to intervention sites were essential to maintain gains during the post-transition period. - Building good relationships was also key for ensuring timely data sharing between government and the Avahan program.   *Ozawa et al. (2016) – India*   - Most targeted interventions that transitioned reported using data for program planning, using pictorial micro-planning tools regularly, and rigorous monitoring of outreach workers. |
| HIV/AIDS | Global Fund to fight AIDS, Tuberculosis, and Malaria (Global Fund) | *Amaya et al. (2014) - Peru*   - During transition, although there were plans to strengthen the monitoring and evaluation systems, data quality was a big concern among all stakeholders involved. Lack of data had a negative impact on planning, goal setting and accountability during transition.   *Eurasian Harm Reduction Network (2016) - Albania*   - Albania’s surveillance and program monitoring system was weak. There was no funding for necessary surveys or population size estimate, and this was identified as a challenge affecting post-transition implementation of harm reduction programs.   *Eurasian Harm Reduction Network (2016) - Romania*   - During Romania’s transition, only non-governmental organizations (NGOs) provided crucial HIV/AIDS program monitoring data. NGO’s however, focus on reporting only quantitative not qualitative data. This resulting in lack of reliable information on care quality, accessibility etc. - Additionally, lack of reliable data also affects budgeting and planning.   *Open Society Foundation (2017) – Multiple countries (Macedonia, Montenegro, and Serbia)*   - Macedonia’s transition challenges included the low quality of existing epidemiological data which continued to create a risk of future budget shortfalls due to insufficient information. Insufficient information made it challenging to make services and funding allocation as effective as possible. - In addition, insufficient information jeopardizes Macedonia’s efforts of reaching global HIV reduction targets. especially if program planning is based on old data. It may lead to additional challenges for programs focused on key populations, particularly men who have sex with men, in the future.   *Global Fund Office of Inspector General (2018) – Global*   - Transition activities are inconsistently captured in the work plan tracking measures of Global Fund’s Grant Performance Framework. Therefore, activities progress can be overlooked during annual funding decision-making processes when the grant’s performance is fully assessed by the secretariat.   *Government of Nepal (2018) – Nepal*   - The Ministry of Health implemented a budget and expenditure tracking record named Transaction Accounting and Budget Control System requiring all the spending units across the country under the ministry to upload their expenditure reports in every trimester. - Nepal at the time of the report needed to introduce surveillance systems for Hepatitis B and Hepatitis C surveillance |
| HIV/AIDS | Presidents Emergency Plan for AIDS Relief (PEPFAR) | *Torpey et al. (2010) – Zambia*   - Lack of national institutionalized quality systems undermined the continuous implementation of quality assurance and quality improvement processes in turn affecting technical sustainability.   *Crye (2011) – Multiple countries (South Africa, Zambia, and El Salvador)*   - In Zambia it was recognized that there was a need to revisit capacity assessments to inform role- and requirement-clarity for local partners during transition. The rationale was to help local partners realize capacity gaps early.   *Vogus and Graff (2015) – Multiple countries (Specific examples from Brazil, Mexico and South Africa)*   - USAID Family planning reviews indicated that successful transition plans were flexible to accommodate changing needs and contexts often identified via mid-term assessments. In Brazil mid-term assessment review recommended additional management components for 2 states and in Mexico a mid-term assessment led to an extension of phase out time frame. - South Africa’s lack of a system to track patients who are lost to follow up as services shifted from non-governmental partners to government clinics made it difficult to measure the scale of the problem and adopt mid-course corrections.   *Kazadi (2015) – Multiple countries (Uganda, Kenya, Tanzania, and South Africa)*   - In Kenya, during the post-transition period, local partners adopted and sometimes scaled-up key systems and tools developed under the AIDSRelief program such as preparing site budgets, site capacity assessments, paperless reporting and IQCare. |
| HIV/AIDS | Global Fund, PEPFAR and other bilateral donors | *UNAIDS (2013) – Multiple countries (Cambodia, Myanmar, South Africa, Swaziland, Kenya, Malawi, Namibia, and Kazakhstan)*   - In Swaziland, developing a more reliable quantification methodology and supplier performance data led to improved anti-retroviral volume estimations, and anti-retroviral drug efficiency. - In South Africa, improving quantification methods by the National Department of Health led to better estimations of anti-retroviral supply which increased confidence among suppliers optimizing production. |
| Immunization | Gavi, the Vaccine Alliance | *Gavi (2014) – Bosnia and Herzegovina*   - In the post-transition period, the information system was unable to provide vaccine coverage and dropout rates. There were problems with assessments of both the denominator (total number of children) and the numerator (children vaccinated).   *Gavi (2015) – Albania*   - Feedback received during projects helped Albania improve inconsistencies in demographic information which was a big challenge in the country. Gavi also helped the country improve its reporting to match international standards.   *Saxenian et al. (2015) – Multiple countries (Bhutan, Republic of Congo, Georgia, Moldova, and Mongolia)*   - Inter-country exchanges and information sharing between countries benefited graduating countries. Visits from high level officials from organizations such as WHO and Gavi also helped raise the profile of immunization issues and inform governments response.   *Learning Network for Countries in Transition (2017) – Ghana*   - As Gavi transitions out of Ghana, weak system of tracking children, weak monitoring and supervision at the district and sub-district level and incomplete data reporting are major challenges.   *Learning Network for Countries in Transition (2017) – Multiple countries (Armenia, Congo, Georgia, Ghana, Indonesia, Lao PDR, Moldova, Sri Lanka, Sudan, Timor-Leste, Uzbekistan, Vietnam)*   - Common challenges experienced during transition from Gavi included lack of data analysis and tools to facilitate decision making, limited capacity for data collection and analysis, lack of legal provisions for surveillance of vaccine preventable diseases (VPD), and inadequate infrastructure for VPD surveillance.   *WHO (2017) – African Region (Specific examples from Nigeria)*   - Nigeria is facing big challenges with data collection and use during Gavi’s accelerated transition phase.   *Cernuschi et al. (2018) – Multiple countries (Angola, Armenia, Azerbaijan, Bhutan, Bolivia, Congo Republic, Georgia, Ghana, Guyana, Honduras, Moldova, Mongolia, Papua New Guinea, Sri Lanka, and Uzbekistan)*   - In transitioning countries from Gavi support 75% of countries reported major data quality issues. Additionally, data indicates shortcomings in the use of evidence-based decision making that may pose more challenges as countries move towards independent decision-making in the post-transition period.   *Gavi (n.d.) – Multiple countries (Sri Lanka and Timor-Leste)*   - WHO, UNICEF with Gavi funding supported a pioneering ‘twinning’ partnership where Timor-Leste’s health professionals learn directly from their peers in Sri Lanka, forging a lasting mentoring relationship between the two countries as both countries graduate from Gavi support. |
| TB | UNDP and Global Fund | *UNDP (n.d.) – Iran*   - The monitoring and evaluation program for TB during Global Fund grant helped to understand the value of data in improving the TB response. The transition plan aimed to integrate monitoring and evaluation to national TB control programs and universities of medical sciences. |
| Eye Care | Swiss Red Cross | *Blanchet and James (2014) – Ghana*   - In the post transition period between December 2006 and July 2008 one of the activities maintained in the 11 hospitals assessed in the study was the compilation of the quarterly statistics. |

**Table 6. Impact of transitions away from development assistance for health on recipient countries healthcare service delivery systems.**

| **Health Issue** | **Donors** | **Findings** |
| --- | --- | --- |
| Family planning | USAID | *Coury and Lafebre (2001) – Ecuador*   - The two largest non-governmental organizations that provide family planning services—the Association for the Well-being of the Ecuadorian Family (APROFE) and the Medical Center for Family Planning and Counseling (CEMOPLAF)—diversified their services to expand into other reproductive health services beyond family planning to ensure income-generation. - These two non-governmental organizations established systems of cross-subsidies, meaning that services that were profitable would help subsidize the services which were not (e.g., contraceptives for rural poor). In 2000, the APROFE sonogram services were 146% self-sustaining and laboratory services were 110%. CEMOPLAF enjoyed a 174% cost recovery from its sonogram services, and 135% from laboratory services.   *Alkenbrack and Shepherd (2005) – Mexico*   - Special programs (i.e., those for adolescent, rural, and indigent populations) were introduced too late into the phase-out and therefore unable to be institutionalized. While these programs made important advancements during the final phase of USAID support, many have since been discontinued. - Non-governmental organizations expanded their clinic reach (i.e., the number of clinics in operation) after phaseout (for example, FEMAP expanded from 30 clinics pre-phaseout to 47 clinics post-phaseout, and MEXFAM expanded from from 2 clinics pre-phaseout to 26 clinics post-phaseout). - Mexico’s contraceptive prevalence rate continued to increase after phaseout, by 0.7% annually.   *Bertrand (2011) – Multiple countries (Honduras, Nicaragua, Paraguay and Peru)*   - The Honduras service provider ASHONPLAFA developed and offered new services to its low middle-to-middle class clientele to subsidize its family planning services and reached 97% self-sufficiency on a very tight budget. However, the number of community-based delivery (CBD) posts dropped from 2,500 to just over 1,500 for lack of an alternative funding source. - PROFAMILIA in Nicaragua attempted to become sustainable by diversifying its services but its role as a supplier of contraception has continued to decrease in recent years, down to approximately 5% of the market. - Withdrawal of USAID funding from the non-governmental organization PROMESA in Paraguay rendered its social marketing program (i.e., a program that sells contraceptives at subsidized prices) unsustainable.   *Chaudry et al. (2012) – Multiple countries (South Korea, Panama, Sri Lanka, Tunisia, Botswana, Thailand, Costa Rica, Swaziland, Colombia, Mexico, Brazil. Ecuador, Morocco, Turkey, Indonesia, Romania, Jamaica, Dominican Republic, South Africa, El Salvador, Paraguay, Nicaragua, Honduras, Peru, Albania, Ukraine, Egypt, Bangladesh, and Zimbabwe). Specific examples from Egypt.*   - Engaging civil society organizations and private sector to provide services and increase sustainability (e.g. Egypt private sector provides majority of family planning services; in Honduras and Ecuador, non-governmental organizations provide family planning services with initial support from USAID).   *Health Policy Project (2013) - Paraguay*   - The contraceptive security committee, established prior to USAID’s phaseout to facilitate a sustainable country ownership, has greatly diminished the incidence of stockouts and increased demand for and access to contraceptives and SRH services.   *Avila et al. (2016) – Nicaragua*   - The country has developed technical, administrative and programmatic capacity to maintain family planning service delivery and adapt to changes as appropriate. - The Ministry of Health has increased the “couple years of protection” (CYP) produced by 63%, from 304,900 in 2007 to 484,467 in 2012. (*Note: CYP is the estimated protection provided by contraceptive methods during a one-year period, based upon the volume of all contraceptives sold or distributed to clients during that period. For each contraceptive method, the CYP is calculated by taking the number of units distributed to family planning clients during a specified period and dividing this number by a constant representing the number of contraceptive units needed or estimated to protect one couple from pregnancy for one year. The values of these constants are method-specific and are derived from empirical evidence on the amount of each method used by a couple during a year*.) |
| HIV/AIDS | Bill and Melinda Gates Foundation (BMGF) | *Sgaier et al. 2013 – India*   - As part of the transition process the Avahan program adjusted its clinic model for managing sexually transmitted infections. It reduced the number of clinics by almost a quarter in 2009–10, from 1,820 to 1,401, as these had higher overhead costs, and it increased the number of referral clinics (community-based) since they had lower costs but no negative impact on clinical performance. - By 2009, the Avahan program covered a quarter of the most-at-risk people in India. The government took over 10% of the interventions in year 1 (2009), an additional 20% in year 3 (2011), and the remaining 70% in year 4 (2012). The interventions dealing with condom distribution were transitioned 75% in years 1 and 2 and 25% in year 3. - The phased nature of transition allowed for proper planning to address and avoid challenges in maintaining service delivery. Outreach and clinic indicators remained stable; condom distribution declined slightly as a result of improved planning to avoid wastage; and HIV testing increased because of the government program’s greater emphasis on testing. - Between 2008 and 2011, there were very large increases in numbers of outreach contacts (818%), condoms sold (2,147%), truckers visiting clinics (1,139%), and truckers treated for sexually transmitted infections (731%) through the technical support offered to the Governments outreach programs. By 2011 the government had developed sufficient capacity and the technical support unit was discontinued. The post-transition performance in HIV outreach to truckers remained strong under the National AIDS Control Organisation’s leadership.   *Bennett et al. 2015 – India (Karnataka, Andhra Pradesh, Tamil Nadu, Maharashtra, Manipur and*  *Nagaland)*   - As part of the transition process…   - Avahan targeted intervention programs had previously delivered most clinical services for general health and sexually transmitted infections through TI-run centers or alternatively had contracts with private clinics for services. During transition, the delivery of clinical services shifted away from TIs to government facilities. Typically this also implied a shift from broad services for sexually transmitted infections, and indeed general health services, toward a narrower focus on integrated counseling and testing for HIV and syndromic treatment for STIs.   - In preparation for transition, Avahan targeted intervention programs achieved high levels of clinical alignment with government facilities and very high referral rates to government integrated counseling and treatment centers.   - In a number of states, as part of the transition process, government sought to sensitize employees at government clinics so as to improve stigma and discrimination previously felt by clients at these facilities. These efforts appeared successful for female sex workers, but less so for men who have sex with men and transgendered populations, and also varied from one state to another. - By 9-12 months after the transition….   - Close to 70% of targeted intervention programs always had sufficient stocks of condoms and medicines, but the remaining 30% had experienced commodity stock outs within a few months post transition largely due to changes in the supply source and schedule.   - Shifts in the source of clinical services resulted in mixed feelings: while many community members appreciated the possibility of being main-streamed into government clinics, and recognized improvements in government provider sensitivity to their needs, they also mourned the loss of general health services. Many resented the focus on testing to the exclusion of other activities. Men who have sex with men and transgender communities appeared particularly adversely affected by the shift. To respond to these issues, BMGF provided additional post-transition support for three years, although the long-term future of community mobilization is unclear.   - Core services (condom distribution and peer counseling) provided by targeted intervention programs continued at similar coverage levels post transition.   *Bennett et al. (2015b) – India*   - In preparation for transition, significant effort was invested in aligning both clinical and non-clinical elements of the program with government norms. For example, most clinical services had to be shifted from direct delivery by the targeted intervention programs (e.g. through clinics at the drop-in-center) to referrals to government health facilities. By 2012, the final year of transition, alignment of Avahan programs with government programs ranged from 74% to 96% (with one exception of procurement practices which could be explained by the use of stockpiling.)   *Rodriguez et al. (2015) – India*   - The number of enrolled members of a target population for a particular intervention was significantly reduced after transition from Avahan to the National AIDS Control Organization. This reduction was due to maximum participant limitations and separation of each individual high-risk group, which had previously been grouped together under Avahan. - Accessing clinical services within government facilities after the transition was primarily viewed as positive by key populations. Key populations expressed greater confidence attending facilities used by the general public and experienced improved interpersonal care at government facilities. Specific benefits identified by key populations included greater availability of clinicians and medicines, improved linkages to other services, and more streamlined and efficient care (e.g., a single blood test for HIV and syphilis). However, although clinical services improved, the range of services provided, including for their children, narrowed after transition. - Some key populations expressed downsides to accessing care in the government facilities, such as a fear of disclosure of STI or HIV status, an inability to “blend in” at public health facilities, and concerns with confidentiality (for female sex workers specifically.)   *Ozawa et al. 2016 - India (Karnataka, Andhra Pradesh, Tamil Nadu, Maharashtra, Manipur and*  *Nagaland)*   - Between 12 months pre-transition and 6 months post transition, stable outcomes were measured for two key service delivery indicators: average percentage of key populations contacted by peer educators per month and average number of condoms distributed per key populations per month. - Transition readiness can explain sustained program delivery in an HIV/AIDS prevention program in India handed over from a donor to the local government. Specifically, targeted intervention programs with greater preparedness prior to the transition were more likely to achieve better service delivery outcomes post-transition, and this was also true for dimensions of the transition readiness scale, including alignment and communication. The relationship between transition readiness and sustained outcomes did not significantly differ by state. |
| HIV/AIDS | Global Fund | *Amaya et al. 2014 – Peru*   - The process of applying for and implementing Global Fund grants emerged as important preparation for setting long-term sustainable policies. For example, by 2006, the provision of free ART was fully funded by the government, which had a positive effect on coverage and was seen as a first step towards sustaining the results of the Global Fund programs (which ended in 2012.)   *Eurasian Harm Reduction Network – Serbia*   - Some but not all harm reduction programs have been sustained following Global Fund’s withdrawal. Opiate substitution treatment (OST) program was sustained and the government also took over HIV prevention programs in 12 prisons. However, needle exchange programs were entirely funded by Global Fund (four programs between 2007-2014 with annual budgets between 30-40 thousand Euros) and so organizations could not continue the coverage after the funding was withdrawn, except in Novi Sad and Vojvodina where local governments stepped in to support HIV prevention programs for key populations. - Non-governmental organizations (NGOs) have tried to continue outreach and needle exchange on a volunteer basis. With Global Fund support, the Belgrade-based NGO Veza provided services to approximately 1,200 injecting drug users annually. However, in December 2014, the Ministry of Health reclaimed the “mobile unit” Veza used for its outreach, and in June 2015 Veza ran out of money to pay rent. - One activist estimates the Global Fund withdrawal will mean that more than 50 organizations working on HIV will lose as much as 90% of their funding, forcing many (like Veza) to close. This will endanger the health and wellbeing of Serbia’s most marginalized, and may lead to spikes in HIV, hepatitis C infections, and AIDS.   *Open Society Foundation (2015) – Multiple countries (Serbia, Thailand, Jamaica, Macedonia, Mexico, and Ukraine)*   - Macedonia: At particular risk are services for people who inject drugs, sex workers and men who have sex with men (MSM), most of which are delivered by non-governmental organizations (NGOs). Until now, the contributions of the Ministry of Health and Ministry of Social Policies to running of these programs have remained below 1%. The Ministry of Health has started a registration process for NGOs to make them eligible to receive funding for HIV prevention. The current social contracting mechanism, however, requires organizations to provide the funds up front and be reimbursed only upon project completion—a requirement most organizations delivering services for key populations cannot meet. - Serbia: Programs serving 3,000 people who inject drugs in seven cities, including in the three largest cities of Novi Sad, Belgrade, and Nis, have drastically cut or stopped services. An NGO that had been reaching more than 3,000 MSM estimates that this year they will reach 500—a drop of almost 85%. - Thailand: Transition from one sub-recipient to another led to stockouts in needles and syringes for as long as six months. Needle and syringe services have been cut; one NGO reports that it was forced to suspend services in 5 out of 19 provinces, with no discussion and no plans to ensure continuity of services for hundreds of people who use drugs. A pharmacy-based voucher scheme, established with previous Global Fund support, has been terminated. - Mexico: The withdrawal of Global Fund led to a severe disruption in harm reduction programs. There was no plan for transition to continue service provision. NGOs report that the distribution of needles and syringes per injecting drug user fell by between 60 to 90% following cessation of Global Fund support.   *Eurasian Harm Reduction Network (2016) – Montenegro*   - One year after the end of Global Fund support, harm reduction programs from two of the major services providers (CAZAS and Juventas, both non-governmental organizations) have been significantly reduced. Due to lack of funds three needle and syringe exchange programs have been reduced to only one (led by Juventas). The other two needle and syringe exchange programs (led by CAZAS) were forcedly closed due to funding issues. CAZAS is still conducting outreach work and cooperating with Juvenats. Juventas still runs drop-in services but their services are poorly funded and with a highly reduced number of outreach workers and drop-in centers. - Methadone Maintenance Therapy (MMT) centers have continued to operate after Global Fund support ended. However, coverage of MMT has not increased.   *Eurasian Harm Reduction Network (2016) – Romania*   - The number of harm reduction non-governmental organizations dropped from 6 to 2 due to the end of external support (by both the Global Fund and UN Office on Drugs and Crime). - Due to the lack of funding, the number of HIV cases among people who inject drugs rose from 3% in 2010 to 18% in 2011.   *Eurasian Harm Reduction Network (2016) – Albania*   - Lack of Government willingness to support the HIV sector after the Global Fund transitioned was seen in the collapse of HIV service provision and harm reduction program in Albania. - The number of non-governmental organizations (NGOs) implementing services to people who inject drugs fell from four to two and two harm reduction NGOs were forced to close as a result of the end of Global Fund support after March 2012. - At the height of Global Fund Round 5 grant assistance, the number of sterile needles/syringes distributed per person who injects drugs rose from 90 per year in 2011 to 104 per year in 2014. In 2016, only a small number of sterile needles/syringes were being distributed by two NGOs. Although only 80-150 people who inject drugs are receiving such sterile/needle syringe program services compared with over 4,100 at the end of Global Fund Round 5 grant implementation in 2012. - Opioid substitution therapy services were also badly affected by the end of the Round 5 Global Fund grant. The number of people who could access methadone had to be considerably reduced from a high of 813 clients in the first quarter of 2012 (the final year of Global Fund support) to 457 in the first quarter of 2014 (a reduction of 356 clients, or nearly 44%). Methadone is only available at one government-run hospital in Tirane.   *ICASO (2016) – Multiple countries (Serbia, Thailand, South Africa) Specific examples from Serbia.*   - Serbia (post-transition): Opiate substitution treatment is available at 26 centers nationwide, largely as a result of Global Fund investment. Since the transition, three have closed while the rest remains sustainable thus far. The government has also assumed responsibility for HIV prevention in 12 prisons previously supported by the Global Fund. For needle exchange programs, the government has not stepped in to fill the gap left by the Global Fund, which had previously supported access to safe injecting equipment to more than 4,000 clients in four major cities. - Serbia (post-transition): key informants from civil society expressed that a key issue during transition, especially with regard to key populations is that how to maintain programming and not just finding resources such as funding.   *UNAIDS Program Coordinating Board Meeting (2016) – For countries receiving funding from Global Fund*   - It was also reported that funding streams can also abruptly disappear causing a loss of vital services for the communities. This is because certain services are deemed to be incongruent with the political priorities of the donors or national governments. But communities know that they are, in fact, critical services and when they are cut, there are grave consequences. People also lose access to prevention services for HIVs and are more susceptible to illness and death. Participants reported that community centers suffered most when funding from Global Fund were cut and there was disappearance of activities like advocacy and of organizations.   *Open Society Foundation (2017) – Multiple countries (Macedonia, Montenegro,and Serbia)*   - Macedonia: There has been closure on needle-exchange program and service interruptions were expected due to lack of pay for trained staff. The (NGO) non-governmental organizations responsible for implementation of the community-based testing and counseling services has managed to sustain that work in the short term with small bridge funds from a pharmaceutical company. EGAL, an NGO providing services for men who have sex with men, closed two drop-in centers, including one that served a Roma-majority area. In 2016, disruption in services resulted in unchecked growth in transmission, and enrollment for treatment was not keeping pace. While HIV transmission continues to grow unchecked, enrollment on treatment is not keeping pace: by mid-2017, only 75% of the 255 people diagnosed with HIV were receiving treatment. - Montenegro: In the year following the termination of Global Fund support, NGO-led prevention services in Montenegro nearly collapsed. The last of the grant funds went to a one-year stock of prevention commodities like needles and condoms. Cazas and Juventas (two primary service delivery NGOs in the country) continued providing some services on a limited basis and in fewer locations, but with reduced scope and impact. Government-provided services fared better than NGO services: Montenegro’s eight testing centers reported only 960 people tested in 2016—a 26% decrease from peak implementation under the Global Fund—with only 15% identifying as key populations. - Serbia: Due to funding reallocation to support Serbia in the aftermath of the flooding, NGO services among key populations collapsed during the more than two-year break in international support that followed Global Fund withdrawal from Serbia. A drop-in center for sex workers run by an NGO received a two-year grant of €100,000 from a Roma-focused EU project, as most of their clients are Roma. Organizations that still operate are operating from limited funding from local governments. Services were non-existent or very limited in the highest populated cities such as Belgrade or Novi Sad.   *Government of Nepal(2018) – Nepal*   - For 2017-18, the government has, for the first time, committed significant resources (USD 1.4 million) for the procurement of anti-retorival therapy and has pledged to fund 100% of ARV procurement in 2018-2019 and 2020-2021. As of 2018-2019, the government will also assume full financial responsibility for the elimination of vertical transmission of HIV program, TB-HIV interventions, and prevention programs for migrants and in prisons and other closed settings. In addition, the government is increasingly taking over support for human resources, commodities and services that have hitherto been supported by the Global Fund. |
| HIV/AIDS | Multiple | *Health Policy Project (2013) – Multiple countries, with specific reference to Indonesia, Mexico, Morocco, and Peru*   - With expected transitions/shifts to country ownership, civil society organizations have raised concerns about the likely decrease in support services to key populations, especially those stigmatized by risk behaviors that are considered illegal in a given country. - The graduation of Mexico and Morocco from USAID support for family planning have gone smoothly. - In Indonesia, the availability of family planning services declined after graduation, with only 20% of districts having a full complement of facilities, and that family planning programs may be reverting to a previous medical model (e.g., with trained midwives not allowed to insert implants). - In anticipation of the scheduled graduation of family planning programs in Peru (which was later postponed), a 2010 analysis revealed high inequalities in access among rural, indigenous, and poor populations, which were masked by national averages that otherwise met USAID graduation criteria. The analysis highlighted that an ambitious decentralization of health care services would pose new challenges to the sustainability of family planning programs, especially for vulnerable groups.   *Cairney and Kapilashrami (2014)*   - Salary decreases had resulted in a disruption of services as employees neglected their responsibilities to seek more secure employment.   *AMFAR 2015 – Multiple countries (Jamaica, Nigeria, South Africa, Ukraine, Vietnam, and Zambia)*   - Nigeria: frequency of stockouts for HIV test kits results in the slow uptake of HIV treatment. - Ukraine: lack of a strong government response to HIV related needs of key populations results in low service coverage for key populations. - South Africa: informants cite well-documented service disruptions associated with the transfer of programmatic oversight from PEPFAR to the national government. Some nonpublic sector PEPFAR-funded providers went out of business after the antiretroviral therapy program was transferred to government control, forcing many patients to search for new healthcare providers in the public sector. One study of 4,000 patients transferred from PEPFAR-operated to government clinics showed that almost 20% were not successfully re-linked to care and may have experienced an interruption in treatment.   *Burrows et al. 2016 – Multiple countries with specific examples for Romania and Mexico*   - Transitions in Eastern Europe and other countries have increased HIV/AIDS emergencies amongst people who inject drugs. - Romania: there has been a 20-fold increase in HIV infections after the withdrawal of Global Fund support and 30% of new cases in 2013 were linked to injection drug as compared to 3% in 2010. - Mexico: The distribution of needles and syringes (per injecting drug user) fell by 60-90% after the Global Fund transition in Mexico where injecting drug is a common risk factor for HIV. This HIV outbreak is linked to the decline in harm reduction services after the Global Fund transition.   *Rodriguez et al. 2017 – Multiple countries with specific examples from Bangladesh, Botswana, China, India, and Romania*   - Romania: when Global Fund support for HIV services ended in 2010, the prevalence of HIV infection among people who inject drugs rose from less than 2% in 2006 to 53% in 2012 - Botswana: sex workers are faced with threats after donor transitions - Bangladesh: donor transitions have led to drops in HIV care and testing services - China: donor transitions have led to declines in the quality and coverage of services for key populations - India: Key populations supported by the BMGF Avahan program weren’t well informed about the transition and didn’t understand the organizational changes. However, key populations were very aware of the service delivery changes as well as the occurrence of community mobilization.   *STOPAIDS n.d. – Multiple countries with specific examples from Thailand and Estonia*   - Thailand: formally transitioned at the end of 2017 but received a significant drop in funds in the preceding years (90% of previous levels). During the transition process, needle and syringe services were cut in five different provinces. Services for prisoners are no longer allowed. A cost-effective pharmacy voucher funded by Global Fund was also terminated due to lack of funding and prioritization. - Estonia: treatment coverage for HIV/AIDS has increased since Global Fund transition, although there remain gaps for case detection and improving access to services for key populations. Overall, the transition path was not smooth, but Estonia has continued to fund services providers, primarily non-governmental organizations, post-transition. |
| HIV/AIDS | Other | *Walsh et al. (2012) – Zambia*   - All Community Based Organizations except for one report reductions in service provision mainly because of the lack of transport to bring patients to the hospital/clinics as well as having no food to bring to houses. - Difficult decisions regarding which orphans and vulnerable children (OVCs) to support arose from reduction of OVC support like from 100 to 70 orphans and from 300 to 110 care givers. - 11 out of 12 organizations which provided HBC reported scaling-down in services when CRAIDS funding ceased, unable to sustain the capacity to deliver and scale up services. |
| HIV/AIDS | PEPFAR and USAID | *Katz et al. 2013 – South Africa*   - As part of the transition process, many of the specialized HIV-treatment centers created by PEPFAR begun closing, so patients were moved into government-run, community-based health care centers. One study at Mc-Cord Hospital in Durban showed that 70-90% of the 4000 patients successfully transitioned to a new clinic. However, many expressed concerns about confidentiality and stigmatization in the new clinic while others expressed frustration about the lack of access to physicians and attentive care.   *Kavanagh 2014 – South Africa*   - As part of the move away from direct PEPFAR support, patients were transferred out of the PEPFAR funded facilities. During the transfer process, some patients reported dauntingly long waits, stigma, and poorly prepared staff. Patients were turned away from overwhelmed public sector clinics that didn’t have enough staff to deal with the influx of patients. - Some clinics have seen rising levels of “lost-to-follow-up” rates and disrupted patient care. Patients in pre-antiretroviral care in particular were not systematically tracked to see if they remained connected to care. One estimate shows that 19% patients on antiretroviral care that were transferred from a PEPFAR facility did not make a first visit to a public sector facility.   *Kazadi 2015 – Multiple countries, with specific examples from Uganda, Kenya, Tanzania*   - Uganda: In the post-transition period, each local partner expanded coverage (number of patients served, geographical catchment areas etc.) with proportionally less funding and largely continue to deliver quality services. In April 2014, Uganda Catholic Medical Bureau (UCMB) managed 19 facilities (up from 12 under AIDSRelief) and doubled the number of patients enrolled on ART at UCMB managed sites to more than 41000. As of May 2014, UCMB managed 13 sites (up from two under AIDSRelief) and had nearly 8,000 patients on treatment. The Children’s Aid Fund (CAF) Uganda managed eight sites (up from four under AIDSRelief) and received nearly twice the funding than it did under AIDSRelief; the number of patients in care doubled (from 12,000 at transition to more than 26,000 patients in 2014) and thus found it necessary to drop complementary program components and seek additional funding to cover programming not included in its post-AIDSRelief award. - Kenya: The two major implementing partners for AIDSRelief, the Christian Health Association of Kenya (CHAK) and the Kenya Conference of Catholic Bishops (KCCB), have substantially increased the number of sites they manage and the number of patients at those facilities. Quality indicators remained good overall and CHAK and KCCB have seen a 218% increase in CDC funding. Nationally, CHAK and KCCB are more involved in technical working groups, further positioning them as technical leaders in Kenya’s HIV care and treatment. As of April 2014, KCCB serves nearly 47,000 patients through 18 facilities (up from 13 facilities under AIDSRelief). Almost 41,000 of these patients are on ART. Since transition from AIDSRelief KCCB reports that they have become even more involved with the government. As of March 2014, CHAK, supported 20 facilities (up from 15 under AIDSRelief) and 37 satellite sites with more than 34,000 patients on antiretroviral therapy and more than 38,000 in care. CHAK expanded services to include cervical cancer screening and community prevention activities, but recent funding reductions have forced the organization to drop those services, to postpone renovations and equipment purchases, and to conduct fewer trainings than under AIDSRelief. - Tanzania: Building upon the 2011 transition under AIDSRelief, Local Partners Excel in Comprehensive HIV and AIDS Service Delivery (LEAD) transitioned all care and treatment centers in Mwanza region to Christian Social Services Commission (CSSC) in January 2013. By March 2014, CSSC had expanded coverage by working with district health management teams to open an additional 25 care and treatment centers, were providing antiretroviral therapy to more than 34,000 clients (current) and reported a 73% retention rate. As of September 2014, more than 1,100 clients returned to care as a result of the “back to care” campaign. - South Africa: Transition from PEPFAR to the Department of Health has typically followed one of two models: 1) Patients are transferred from stand-alone local partner facilities to a government-run, integrated primary health care setting that can provide comparable HIV services. or 2) If a public facility or comparable public HIV services are not available, the Department of Health will provide a partner serving that area (e.g., a faith-based hospital) with funds for HIV-related medicines and laboratory services or some salaries. Through AIDSRelief, IYDSA, a local non-governmental organization based in the Eastern Cape Province of South Africa developed five antiretroviral therapy sites and 10 clinical outreach centers in underserved communities. Since the closure of AIDSRelief, St. Mary’s a Catholic hospital serves patients in close collaboration with (and with subsidies from) the Department of Health. - Building on its existing HIV care and support services South African Catholic Bishops Conference (SACBC) maintained service delivery throughout South Africa, Botswana, and Swaziland through 22 sites under AIDSRelief and maintained good “lost-to-follow-up” (22 to 23%) and mortality rates (3 to 4%) for two years after the first transition. Since treatment is now almost entirely within the government’s purview, as of May 2014, SACBC had transitioned 20 of its 22 sites to the government and reassessed what services it could provide in pursuit of its efforts to mitigate HIV’s impact in South Africa. - Based on Catholic Relief Services comparison data between AIDSRelief and three local partner patient enrollment on antiretroviral therapy for years 2012-2013 and patient enrollment in Kenya, Tanzania and Uganda at local partners in 2014, the range of increase in patient enrollment on antiretroviral therapy was between 24% and 40% (with an average of 33%). Uganda had the highest increase in patient enrollment (40%). Local partners-initiated care and treatment services at 57 additional facilities and the number of patients enrolled on ART increased by 53%.   *Vogus and Graff 2015 – Countries graduated from PEPFAR by 2013 in Latin America, Caribbean and countries under transition from PEPFAR in 2013 (specific examples from South Africa, Brazil, and Mexico)*   - Monitoring and evaluation, especially mid-transition, can help facilitate smooth transition to accommodate changing needs. In South Africa, a critical failure of its transition process was the lack of system to track patients who were lost to follow-up after services shifted from NGOs to government clinics. In Brazil, however, the family planning transition incorporated findings from a midterm assessment, which validated the strategy and recommended additional management components for 2 states. In Mexico, a midterm assessment led to an extension of the phase-out time frame.   *Health Policy Project (2016) – Multiple countries, with specific examples from Bangladesh and China*   - Bangladesh: After PEPFAR funds were withdrawn in 2014, there was a decrease in HIV testing and counseling coverage and the quality of care. Government clinics were able to absorb some of the programming previously funded by PEPFAR, but these programs were not always friendly towards key populations. Government clinics were also unable to effectively contract civil society organiations (CSOs) for HIV services. - China: China primarily funds its HIV response and has sustained service levels after transition through new domestic contracting mechanisms that enables some CSOs to provide key population targeted services. However, some stakeholders share that the quality and coverage of services is inadequate, as it only covers basic testing and treatment without allowing CSOs the flexibility or autonomy to innovate or respond to communities’ changing needs. Men who has sex with men remain have an increasing HIV prevalence and concerns remain on how to best reach this population.   *Health Policy Project (2016) – Bangladesh*   - The PEPFAR funded Modhumita program at one point funded approximately 75% of HIV testing in the country through its Modhumita Health Centers (MHCs). After initial funding cuts, nine MHCs closed. After further reductions, only 16 of the 41 MHCs were able to remain open, primarily due to another donor (UNICEF) stepping in with emergency funds. However, the government did open 20 additional clinics to make up for the loss, 12 of which were government operated and 8 were run by civil society organizations. However, stakeholders report reduced coverage and quality of care in these remaining health clinics, with widespread stigma and discrimination for key populations. Additionally, when the government took over procurement of antiretrovirals in 2012, there were delays in medication procurement and delivery. - The short transition timeframe (one year) resulted in gaps in HIV testing and treatment coverage.   *Health Policy Project 2016 – China*   - Key informants estimate that the number of HIV civil society organizations (CSOs) in China dropped by more than two-thirds (from 1,500 CSOs in 2011 to 476 CSOs in 2014) after donors withdrew funding. Many of those that did survive were forced to downsize. However, there is conflicting evidence on whether or not closures/downsizing directly reduced intervention coverage, depending on the source (either key populations and the government.) - Multiple civil society stakeholders stated that the domestic funding mechanism is too quota-focused, pays insufficient attention to quality, and may provide a perverse disincentive to appropriate targeting. For example, informants relayed accounts of professional “testees” who go from one testing site to another, collecting payments and in-kind incentives. So while data may show that HIV testing has gone up, the number of individuals tested may not have increased. - Key informants felt that at local China CDC offices, where most HIV services are delivered, little attention is paid to the capacity of the peer workers to deliver interventions effectively and to follow-up with services for people testing positive, both of which affect program quality.   *Marten (2017) – Tanzania*   - During PEPFAR’s transition to country-ownership, an estimated 36% of people living with HIV were lost to follow-up after three years of treatment. This is believed to be due to PEPFAR’s emphasis on quantitative targets rather than the quality of services provided. - Reduced funding from PEPFAR led to a reduction in services, particularly funding for services such as food and transport, which increase access to services and support antiretroviral therapy adherence. |
| Immunization | Gavi | *Gavi (2015) – Albania*   - Stable and high immunization coverage rates (> 95%) for all antigens included in the national immunization schedule have been sustained post-transition and only three districts out of 36 districts have relatively low coverage rates (92%-94%). - Post transition, auto-disable syringes are used throughout Albania and safety boxes are available in all facilities. - Other non-vaccine immunization related activities like operational expenses for travel for vaccine collection and distribution from central to district levels are fully funded by the government post-transition, however the related expenses were not always reimbursed to health care providers.   *Results for Development (2017) – Multiple countries (Armenia, Congo, Georgia, Ghana, Indonesia, Lao PDR, Moldova, Sri Lanka, Sudan, Timor-Leste, Uzbekistan, and Vietnam)*   - Countries articulated challenges related to achieving better immunization coverage and equity in the Gavi transition. |
| Immunization | Multiple donors | Kerr (2017) – *Multiple countries, specific examples from the Republic of Congo and Sri Lanka*   - Republic of Congo: immunization rates (which is an implication of interruption of service delivery due to loss of funding as well as government’s budget decreasing due to domestic financial challenges such as the oil price collapse) have fallen from 90% to 80% in just one year and polio and measles coverage is only 80% currently. - Sri Lanka has taken full country ownership of its immunization program and has ensured exceptionally high immunization rates, achieving Global Vaccine Action Plan coverage and equity targets in advance. DTP3 coverage has been above 97% since 1990 and over 95% consistently in all regions since 2000. |
| Multiple health issues | Global Fund | *Lewis et al. (2015) – Brazil*   - Both the malaria and TB programs are being sustained post-transition. However, bed net procurement did face some delays after transition.   *TB Europe Coalition (2016) – Multiple countries, specific examples from Azerbaijan, Croatia, and Serbia*   - Croatia: Ten years after the withdrawal of Global Fund, Croatia has succeeded in sustaining the status quo of the national HIV response achieved through the additional inputs of the Global Fund and in expanding many of its components. For example, the average number of people in HIV testing and counselling centers almost doubled in the period 2009-2014 compared to the 2003-2008 period, indicating a scaling up of services after the transition to domestic funding. - Azerbaijan: The withdrawal of the Global Fund has caused a negative impact on community-based services offered by the NGOs. In particular, psychosocial support to TB patients decreased, causing concerns regarding treatment adherence, particularly among key populations such as ex-prisoners, and drug resistance. - Serbia: The Global Fund support for HIV prevention and harm reduction projects ended in 2014. It allowed Serbia to scale up harm reduction services between 2006 and 2014, including needle exchange programs, Opiate Substitution Treatment, and outreach activities. The Serbian government pledged to maintain these services after the Global Fund’s departure. However, as of July 2015, the government was supporting only a small proportion of these programs, failing to fill the funding gap. In Novi Sad and Vojvodina only, local governments provided small amounts of funding to NGOs working with people who use drugs. Programs that served 3000 people who inject drugs in seven cities have been drastically cut. The 2015 national budget did not include any funding for prevention for key populations and only 3% of the HIV budget is allocated to prevention.   *TB Europe Coalition (2017) – Bulgaria*   - Non-governmental organizations (NGOs) who historically provided services linked to HIV outreach and prevention have lost their funding since Global Fund support ceased and are either working voluntarily or have had to let go of their staff. Only those that receive TB funding can continue the provision of services until the Global Fund TB grant comes to an end in September 2018. As a temporary measure, Regional Health Inspectorates, who have previously been engaged in the Global Fund grant implementation, are to provide outreach and prevention services, financed by domestic sources, until another contracting solution for NGOs is found. Regional Health Inspectorates have been instructed to hire outreach workers who have experience under former Global Fund grants. However, the current coverage of service is limited and includes only HIV testing. |
| Multiple health issues | Multiple donors (specifically focuses on Global Fund and US) | *CRAT Via Libre 2017 – Guyana*   - Many prevention programs have been discontinued due to decreases in funding for civil society organizations. At present, the national budget does not include support for civil society organizations to conduct any kind of prevention programs, particularly with key populations. - Civil society organizations have always played a pivotal role in the provision of care and support to key populations, as well as orphans and vulnerable children (OVC). Organizations such as CPIC (Monique’s Caring Hands) and Lifeline have been engaged in the provision of care and support to key populations as well as to OVC. Their care and support programs have been possible through external funding; however, as funding decreases, these organization have had to reduce or close their programs. This is a key area of concern, as care and support are an important component of the continuum of care, especially for HIV and TB.   *Teixeira et al. 2017 - Vietnam*   - To provide services for some diseases such as TB, Vietnam relies primarily on external funding. The WHO’s Vietnam country profile indicates that in 2016, only 9% of the estimated TB requirements were funded domestically, 22% were funded by external sources, and 69%of the TB programs went unfunded. |
| Multiple health issues | Other | *UNDP 2016-2017 – Multiple countries, specific example from Sudan*   - UNDP provided support in Sudan to establish the first national principal recipient Project Implementation Unit and a participatory capacity development planning process was jointly facilitated with UNFPA to strengthen civil society organizations working on HIV prevention with key populations. |
| Multiple health issues | USAID | *Martin et al. 1999 – Multiple countries (Morocco, Indonesia, and Tunisia)*   - USAID transition in Tunisia is an example of the value of assessing government capacity. USAID ended its population, health, and nutrition work in Tunisia in the 1990s faced capacity gaps due to overestimating the implementation capacity of the government and the private sector did not take on the service delivery role as expected. |
| Nutrition | Other | *Rogers and Macías 2004 – Multiple countries, specific examples from Paraguay, Cape Verde, and Jamaica*   - In Paraguay, the Ministry of Education expressed its commitment to taking over school feeding after World Food Program exit, but because of the poor economy, lack of resources and possibly the lack of political commitment within the Ministry, no domestic food procurement was implemented. - In Cape Verde, the national government similarly committed itself to taking over the World Food Program school feeding program, but the commitment was based on its intention to seek an alternative source of external funds, not on commitment of its own resources. As a result, at the time of program evaluation 75% of students had moved to a program managed by the government, but that program was in disarray, providing only a snack instead of a meal, and was eventually terminated in 2001. - In contrast, the Jamaican school feeding program was able to continue, fully funded by the national government, after World Food Program withdrew its food, due in part to the Jamaican government food production facilities that supplied the school feeding program. |
| Nutrition | USAID | *Rogers and Coates 2015 – Multiple countries, with specific examples from Honduras, Kenya, and Bolivia*   - In India, social health activists who were paid a goal specific salary (immunization, hospital deliveries) performed better than child health workers (CHWs) who were paid by the Government. - Provision of resources (such as free supplementary food in maternal and child health and nutrition projects or free marketing services in agriculture projects) created expectations in many projects that could not be sustained once resources were withdrawn. In Kenya and Honduras, participation in growth monitoring fell significantly after food supplements were withdrawn; in Bolivia, government provision of conditional cash transfers (which were implemented independently of the USAID Office of Food for Peace projects) replaced food supplements as an incentive, and growth monitoring participation was better sustained. However, Government health services were not always linked to a CHW so these communities may not have benefited from the home visits and other services that CHWs used to provide. |
| Other | Global Fund | *Wang et al. 2017 – China*   - Based on annual data reported to WHO, MDR-TB patient enrolment in treatment sites previously supported by the MDR-TB grant declined nationwide by 70% in the two quarters following the exit of the Global Fund. - Changes to health system delivery and financing will potentially impact TB and MDR-TB control. TB diagnostic and treatment services are shifting from services delivered by community organizations to specialized public hospitals. As such, public health hospitals will be at the core of the TB continuum of care and control. |
| Other | Other | *Blanchet and James 2014 – Ghana*   - Only 2 activities were maintained in all 11 hospitals: outpatient consultations and compilation of quarterly statistics. Cataract surgeries continued in 58% of the hospitals (i.e., 7 hospitals), Outreach activities were maintained in 33% of hospitals (i.e., 4 hospitals) and only 1 hospital continued school health between December 2006 and July 2008. School health screening was the least sustained activity since it was the most incompatible and most complex activities, as well as the least triable and observable activities while facility-based consultations were more likely to be routinised because they were perceived as very compatible, and not complex.   *UNDP n.d. – Iran*   - Transition was smooth and testing levels post-transition indicate sustainability of activities. - All TB laboratories were transitioned to national management and the number of Drug Susceptibility testing done and treatment success rates in the country during 2015 showed that this activity has been sustainable. - The Global Fund TB Program had supported the establishment, equipping and/or upgrading of 40 culture laboratories, 66 Direct Smear Microscopy laboratories (DSM), and 8 Drug Susceptibility Test (DST) laboratories in the provinces to provide TB diagnosis services to the entire country. The established labs improved the TB detection and notification rate with a significant contribution to diagnosis of Multi Drug Resistant TB (MDR-TB) in the country. This was securely transferred over to the Government as all requirements were met and a Human Resource Plan was developed to enable a sustainable and effective use of the facilities on transition, with enough budget allocations at national and provincial level. |

**Table 7. Impact of transitions away from development assistance for health on health outcomes in recipient countries.**

| Health issues | Donors | Findings |
| --- | --- | --- |
| HIV/AIDS | Global Fund to fight AIDS, Tuberculosis, and Malaria (Global Fund) | *Eurasian Harm Reduction Network (2016) – Montenegro*   - Despite the lack of government support and challenges in service delivery, Montenegro was able to maintain a low prevalence of HIV among key populations. Yet, there are concerns about maintaining low prevalence in long-term, especially among persons who inject drugs.   *Eurasian Harm Reduction Network (2016) – Romania*   - Post transition there was a direct correlation between dramatic decrease in funding for harm reduction services, especially needle/syringe program and a substantial increase in HIV/hepatitis transmission among persons who inject drugs   *Open Society Foundation (2017) – Multiple countries (Macedonia, Montenegro, and Serbia)*   - In Montenegro data from 2014 indicates that infections among men who have sex with men were already rising during the withdrawal of Global Fund that resulted in cessation of services from non-governmental organizations. - In Serbia, Global Fund support ended in 2014 and HIV rates increased from 2014 to 2015 with men who have sex with men accounting for 73% of new infections. |
| HIV/AIDS | Presidents Emergency Plan for AIDS Relief (PEPFAR) | *Kazadi (2015) – South Africa*   - In South Africa, during the post transition period, the Sothern African Catholic Bishops Conference (a transitions partner) built on its existing HIV care and support services and delivered anti-retro viral therapy through 22 sites under AIDSRelief. They also maintained relatively low loss to follow up rates (22 to 23%) and mortality rates (3 to 4%) for two years after the first transition. |
| Nutrition | USAID | *Rogers and Coates (2017) – Multiple countries (Bolivia, Honduras, India, and Kenya)*   - In Kenya childhood stunting prevalence were maintained at the same level or improved in areas managed by CARE but increased in areas managed by Food for the Hungry (FH). In FH managed areas stunting prevalence increased from 29% at end-line to 40% at follow up. This was partly due to the severe food emergency that afflicted FH areas at the time of follow up in 2011. - In Bolivia and Honduras, stunting that declined during project period were sustained or declined further by the time of follow-up. - In India, although areas managed by Catholic Relief Services showed overall improvement in stunting prevalence during the project period results at follow-up were inconsistent by state. This was also the case for areas managed by CARE. For example, stunting prevalence increased in Andhra Pradesh, remained the same Chhattisgarh and Orissa, and decreased significantly in Uttar Pradesh. |
